# Fetal exposure to bisphenols and phthalates and childhood bone mass: a population-based prospective cohort study

**DOI:** 10.1016/j.envres.2020.109602

**Published:** 2020-05-01

**Authors:** Charissa van Zwol - Janssens, Leonardo Trasande, Alexandros G. Asimakopoulos, Maria-Pilar Martinez-Moral, Kurunthachalam Kannan, Elise M. Philips, Fernando Rivadeneira, Vincent W.V. Jaddoe, Susana Santos

**Affiliations:** a The Generation R Study Group, Erasmus MC, University Medical Center Rotterdam, the Netherlands; b Department of Pediatrics, Erasmus MC - Sophia Children’s Hospital, University Medical Center Rotterdam, the Netherlands; c Department of Pediatrics, New York University School of Medicine, New York City, NY, 10016, USA; d Department of Environmental Medicine, New York University School of Medicine, New York City, NY, 10016, USA; e Department of Population Health, New York University School of Medicine, New York City, NY, USA; f New York Wagner School of Public Service, New York City, NY, 10016, USA; g New York University Global Institute of Public Health, New York City, NY, 10016, USA; h Wadsworth Center, New York State Department of Health, Department of Environmental Health Sciences, School of Public Health, State University of New York at Albany, Albany, NY12201, United States; i Department of Chemistry, The Norwegian University of Science and Technology (NTNU), 7491, Trondheim, Norway; j Biochemistry Department, Faculty of Science, King Abdulaziz University, Jeddah, Saudi Arabia; k Department of Internal Medicine, Erasmus MC, University Medical Center Rotterdam, the Netherlands

**Keywords:** Endocrine disruptor, Bisphenol, Phthalate, Bone mineral density, Bone mineral content

## Abstract

**Background:**

Exposure to bisphenols and phthalates might influence bone health. We hypothesized that exposure to bisphenols and phthalates during fetal life has persistent effects on bone development.

**Objectives:**

To analyze the associations of fetal exposure to bisphenols and phthalates with bone health in school-aged children.

**Methods:**

Among 1,362 mother-child pairs participating in a population-based cohort study, we measured maternal urinary concentrations of bisphenols and phthalates at first, second and third trimester with high performance liquid chromatography electrospray ionization-tandem mass spectrometry. Total body bone mineral density (BMD) and bone area (BA) were measured using dual-energy X-ray absorptiometry (DXA) at 6 and 10 years, and were both used to calculate bone mineral content (BMC) and area-adjusted BMC (aBMC, a measure of volumetric BMD).

**Results:**

Maternal bisphenol concentrations were not associated with childhood bone measures at 6 years. After adjustment for covariates and multiple testing correction, an interquartile range increase in maternal first trimester bisphenol S (BPS) concentrations was associated with lower BMD and aBMC at 10 years (−6.08 (95% confidence interval (CI), −9.97 to −2.19) mg/cm^2^ and −0.12 (95% CI, −0.20 to −0.04) g). Maternal third trimester low molecular weight (LMW) phthalate concentrations were associated with higher aBMC at 6 years whereas, maternal third trimester di-n-octylphthalate (DNOP) concentrations were associated with lower aBMC at 10 years. However, these associations did not remain statistically significant after multiple testing correction.

**Discussion:**

Maternal first trimester BPS concentrations are associated with lower BMD and aBMC in school-aged children. These findings should be considered as hypothesis generating and need further replication and exploration of potential underlying mechanisms.

## 1. Introduction

Early-life exposure to environmental factors may lead to adaptations that permanently affect skeletal development and subsequently predispose individuals to impaired bone health and osteoporosis during the life course (Cooper et al., 2006). Endocrine disruptor chemicals (EDCs), such as bisphenols and phthalates, are adverse environmental factors which can alter the systemic hormonal regulation of the bone remodeling process and the skeletal formation (Agas et al., 2013). Bisphenols and phthalates are able to cross the placenta-blood barrier and may affect the developing fetus (Schonfelder et al., 2002; Silva et al., 2004). They act as selected modulators of estrogen, androgen, thyroid, and other receptors by activating several signaling cascades (Schantz and Widholm, 2001). These hormones play a major role in bone formation and remodeling, so disruption might affect bone strength, architecture, and density parameters such as the bone mineral density (BMD) and content (BMC), the bending force, the hardness, and the plasticity of bones (Miettinen et al., 2005; Hermsen et al., 2008; Lind et al., 2009; Finnila et al., 2010; Rowas et al., 2012).

Thus far, studies on the associations of bisphenol and phthalates with skeletal health in humans are scarce. Small studies among post-menopausal women suggested that bisphenol A (BPA) and phthalate exposure may unfavorably affect bone homeostasis, which might lead to osteoporosis (Vitku et al., 2018; Min and Min, 2014). Fetal life seems to be a critical period for EDCs exposure because organs and systems are changing more rapidly during this period (Golub et al., 2004; Anway et al., 2005). To the best of our knowledge, no previous studies have been performed focused on the early-life exposure to bisphenols and phthalates in relation to bone health in humans. In addition, experimental animal studies reported sex-dependent associations. After exposure to BPA and phthalates in early-life, an increased BMC was observed among male rodents, whereas either a decreased BMC or an unchanged BMC was observed among female rodents (Lejonklou et al., 2016; Pelch et al., 2012; Chin et al., 2018). Based on these previous studies, we hypothesized that fetal exposure to bisphenols and phthalates has adverse sex-specific effects on childhood bone development.

Therefore, in a population-based cohort of 1,362 mothers and their children, we examined the associations of fetal bisphenol and phthalate exposure with bone health outcomes in school age children. We explored whether any association differed for boys and girls.

## 2. Methods

### 2.1. Study design

This study was embedded in the Generation R Study, a population-based prospective cohort study among 9,778 mothers and their children followed from early fetal life onwards in Rotterdam, the Netherlands (Kooijman et al., 2016). Bisphenol and phthalate concentrations were measured among a subgroup of 1,405 mothers with three urine samples available in pregnancy and whose singleton children also participated in postnatal studies (Philips et al., 2018). We excluded mothers without information on bisphenol and phthalate urinary concentrations for at least one trimester during pregnancy and whose children had no information about bone health at both 6 and 10 years. The population for analysis comprises 1,362 mother-child pairs (1,335 at 6 years and 982 at 10 years; flow chart is given in Supplemental Fig. S1). The study protocol underwent human subjects review at Erasmus Medical Center, Rotterdam, The Netherlands (MEC 198.782.2001.31, MEC-2007–413). Mothers provided written informed consent for themselves and their children (World Medical Association, 2013).

### 2.2. Maternal bisphenol and phthalate concentrations

Bisphenol and phthalate concentrations were measured in a spot urine sample obtained from each participant at early (median 12.9 weeks of gestation, 25th,75th percentiles 12.1, 14.5), mid (median 20.4 weeks of gestation, 25th,75th percentiles 20.4, 20.9) and late (median 30.21 weeks of gestation, 25th,75th percentiles 29.9, 30.8) pregnancy. These periods were considered as first, second and third trimester. All urine samples were collected between February 2004 and July 2005, and stored at −20 °C in polypropylene vials. Quantitative detection of phthalate metabolites was achieved utilizing a solid-phase extraction method and detection of bisphenols was achieved utilizing a liquid-liquid extraction (HPLC-ESI-MS/MS), both performed by a high performance liquid chromatography electrospray ionization-tandem mass spectrometry. Further details on collection, transportation, and analysis methodology have been described previously (Philips et al., 2018). We grouped bisphenols together and grouped phthalate metabolites according to their molecular weight categories and parent compounds. Individual bisphenol and phthalate metabolites were only included in groups and assessed individually if, less than 80% of the sample concentrations were below the limit of detection (LOD). We calculated the weighted molar sums for groups representing total bisphenols, low-molecular-weight (LMW) phthalates, high-molecular-weight (HMW) phthalates, and for two subgroups within HMW phthalates, namely di-2-ethylhexylphthalate (DEHP) and di-n-octylphthalate (DNOP) metabolites. Phthalic acid (PA) was analyzed separately as a proxy for total phthalate exposure. Bisphenol and phthalate concentrations below LOD were substituted by LOD/√2 (Philips et al., 2018). To account for urinary dilution, urinary concentrations of phthalates and bisphenols were converted to μg/g creatinine (for the separate metabolites) or μmol/g creatinine (for the metabolite groups). To reduce the potential for exposure misclassification due to temporal variability, we calculated the overall mean exposure during pregnancy by summing the first, second and third trimester bisphenol and phthalate concentrations and dividing by three.

### 2.3. Bone mineral density measurements

At both 6 and 10 years, total body bone mineral density was measured using a dual-energy X-ray absorptiometry (DXA) scan (iDXA, General Electrics— Lunar, 2008, Madison, WI, USA), as previously described (Heppe et al., 2013). Scans were analyzed using enCORE version 13.6. In a sample of non-obese adults, the coefficient of variation of total body BMC using the GE Lunar iDXA system was 0.5%, showing high precision for assessing bone composition (Rothney et al., 2012). The official positions of the International Society for Clinical Densitometry (ISCD) on DXA evaluation in children and adolescents (males and females 5–19 years) indicates that when technically feasible, the lumbar spine and whole body BMD and BMC are the most accurate and reproducible skeletal sites for performing BMD and BMC (Bianchi et al., 2010). In our analyses, we used areal total body less head BMC, bone area (BA) and BMD as recommended by the ISCD (Lewiecki et al., 2008). BMD measured by DXA was expressed as BMC (in g) per projected BA (in cm2). BMC was calculated from BMD using the projected bone area. Area-adjusted BMC (aBMC) was derived as a measure of volumetric BMD by using linear regression to adjust BMC for bone area and adding the residuals to the mean BMC (Macdonald-Wallis et al., 2010).

### 2.4. Covariates

Information on maternal age at enrolment, parity, educational level, ethnicity, pre-pregnancy weight, folic acid supplementation, smoking habits and alcohol consumption was obtained from questionnaires during pregnancy. Maternal height was measured at enrolment and pre-pregnancy body mass index (BMI, kg/m^2^) was calculated. Maternal 25(OH)D concentrations in second trimester were measured in plasma using isotope dilution liquid chromatography-tandem mass spectrometry. Vitamin D status was categorized, according to previously used cut-offs and recommendations, into severely deficient (< 25.0 nmol/L), deficient (25.0 to 49.9 nmol/L), sufficient (50.0 to 74.9 nmol/L) and optimal (≥75.0 nmol/L) (Miliku et al., 2016). We obtained information about child’s sex from midwife and hospital registries. At both 6- and 10-year visits, child height was measured without shoes. Measures of total body fat and lean mass were derived from the DXA scan.

### 2.5. Statistical analysis

For all analyses, we natural log-transformed phthalate and bisphenol concentrations to reduce variability and account for right skewedness of the distribution and standardized by the interquartile range to ease the interpretation of effect sizes. We performed linear regression models to assess the associations of exposure to bisphenol and phthalate (at first, second and third trimester and overall mean during pregnancy) with childhood bone measures. Non-linearity of the relationship was visually assessed and ruled out. Basic models include child’s sex, age and height at visit. Potential confounders were identified based on the graphical criteria for confounding by visualizing a directed acyclic graph (DAG) presented in Supplemental Fig. S2) and were included in the models those that changed the effect estimates > 10% for at least one of the outcomes (Santos et al., 2019). To account for the influence of body composition on bone measures, we also adjusted for the sum of child’s lean and fat mass. Analyses were performed for the total group and by sex. Since folic acid is known to influence the metabolism of bisphenols and phthalates (Crider et al., 2012), we tested for statistical interaction between bisphenols and phthalates with folic acid supplementation in these associations. We found statistically significant interactions and thus stratified analyses by folic acid supplementation were performed. To correct for multiple hypothesis testing, each p-value was compared with a threshold defined as 0.05 divided by the effective number of independent tests estimated based on the correlation between the exposures (p-value threshold of 0.0098) (Li et al., 2012).

To examine the independent associations of maternal first, second and third trimester phthalate and bisphenol concentrations, we performed a sensitivity analysis in which the exposures at all three trimesters were simultaneously included in one model, creating a mutually adjusted model. Also, as sensitivity analyses, instead of using creatinine-adjusted first, second and third trimester phthalate and bisphenol concentrations, we refitted the models by adding creatinine concentration as a separate covariate. Due to the large proportion of concentrations below LOD for some bisphenols, we additionally performed the models by categorizing the bisphenols as detected (above LOD) and undetected (below LOD). To maintain statistical power and reduce bias related to missing data on covariates, we performed multiple imputation according to the Markov Chain Monte Carlo method. The percentage of missing values for covariates ranged from 0.6 to 20.2%. Ten imputed datasets were created and no substantial differences were found between the original and imputed datasets. We present results based on pooled imputed datasets. All statistical analyses were performed using the Statistical Package of Social Sciences version 25.0 for Windows (SPSS Inc., Chicago, IL, USA).

## 3. Results

### 3.1. Participant characteristics

Table 1 shows the characteristics of the included mothers and their children. Non-response analyses at age 10 showed that mothers of children without follow-up data available were more likely to have higher urinary phthalate concentrations, be younger, of non-European ethnicity, less educated, not take folic acid supplementation, more severely vitamin D deficient and not have consumed alcohol during pregnancy (Supplemental Tables S1 and S2). Table 2 and Supplemental Table S3 show the concentrations and detection rates of all bisphenols and phthalate metabolites for the total group and by sex, respectively. The intraclass correlation coefficient for phthalates and bisphenols across pregnancy varied between 0.01 and 0.40.

### 3.2. Maternal bisphenol urinary concentrations and childhood bone health

We did not observe associations of maternal total and specific bisphenol concentrations with childhood bone measures at age 6 (Table 3 and Supplemental Tables S4 and S5). Also, no associations were observed for maternal BPA concentrations with childhood bone measures at both ages. In the fully adjusted model, an interquartile range increase in maternal first trimester BPS concentration was associated with lower childhood BMD and aBMC at 10 years (−6.08 (95% confidence interval (CI), −9.97 to −2.19) mg/cm^2^ and −0.12 (95% CI, −0.20 to −0.04) g, respectively). These associations remained significant after adjustment for multiple testing correction (Table 3). Results of the basic models are given in Supplemental Tables S6 and S7. We did not observe different associations between child’s sex or maternal folic acid supplement use groups (Supplemental Tables S8 and S9).

### 3.3. Maternal phthalate urinary concentrations and childhood bone health

In the fully adjusted model, an interquartile range increase in maternal third trimester LMW phthalate concentrations was associated with higher childhood aBMC at 6 years (0.08 (95% CI 0.004 to 0.15) g). Also, an interquartile range increase in maternal third trimester DNOP concentration was associated with lower aBMC at 10 years (−0.07 (95% CI −0.14 to −0.003) g). However, none of these associations remained statistically significant after multiple testing correction (Table 4 and Supplemental Tables S10 and S11). No associations were observed for the other maternal phthalate metabolite concentrations with bone measures at both ages. Results from the basic models are given in Supplemental Tables S12 and S13. We observed no consistent sex-specific differences for the associations of maternal phthalate metabolite concentrations with bone health outcomes (Supplemental Table S14). When we stratified for folic acid use, we observed that maternal phthalate metabolite concentrations tended to be inversely associated with bone measures in women who took folic acid supplementation but positively associated in those that did not take folic acid supplementation (Supplemental Table S15).

### 3.4. Sensitivity analysis

As compared to the trimester specific results as described above, similar results for the associations of maternal bisphenol and phthalate concentrations with childhood bone health at both ages were observed in the mutually adjusted models (Supplemental Tables S16, S17, S18 and S19). Results of the models using the uncorrected maternal urinary concentrations of phthalates and bisphenols (in nmol/L) and adding creatinine as a separate covariate in the model were similar to the main analyses with creatinine-adjusted bisphenol and phthalates concentrations (Supplemental Tables S20 and S21). Similar results were also observed when we categorized the bisphenols as detected (above LOD) and undetected (below LOD) (Supplemental Table S22).

## 4. Discussion

We observed, in a population-based prospective cohort study, that higher maternal first trimester BPS concentrations were associated with lower childhood BMD and aBMC at 10 years, but not at 6 years. Furthermore, maternal LMW phthalate and DNOP concentrations may affect childhood bone health outcomes. We did not find any consistent sex-specific associations.

### 4.1. Interpretation of main findings

Results from previous studies suggest that fetal life is a critical period for EDCs exposure because organs and systems are changing more rapidly during this period (Golub et al., 2004; Anway et al., 2005). We examined whether fetal exposure to bisphenols and phthalates has adverse, and sex-specific effects, on childhood bone development. Urinary concentrations and detection rates of bisphenols and phthalate metabolites found in our study population were generally lower compared to other Western studies performed in the same time period (Casas et al., 2013; Harley et al., 2017; Philippat et al., 2014; Valvi et al., 2013; Woodruff et al., 2011). Three cross-sectional studies have been performed to examine the relationship between BPA level and bone mineral density in humans but they yielded no associations (Kim et al., 2012; DeFlorio-Barker and Turyk, 2016; Zhao et al., 2012). To the best of our knowledge, no previous study has examined relationships between other BPA analogues and bone mineral density in humans. We observed that higher maternal first trimester BPS concentrations were associated with lower childhood BMD and aBMC at 10 years. These results are in line with previous in vitro studies, which found that long-term exposure to BPS and bisphenol AF, but not BPA, induces changes in the genome-wide gene expression assay, resulting in developmental changes of embryonic skeletal system, enhanced osteoclast differentiation and hedgehog signaling pathway (Fic et al., 2015). The differential effects of BPA analogues on skeletal process might be related to their affinity towards cell receptors. However, no associations were observed with bone health in children at age 6. This age dependent associations may be due to a lower variability of the bone mass outcomes at age 6 compared to age 10, precluding the detection of associations. The effect of bisphenols and phthalates on bone development might also only become more apparent at later ages.

In animal studies, phthalate metabolites may cause dose-dependent fetal toxicity reflected in severe skeletal malformations and imbalance of bone homeostasis (Agas et al., 2013). In line with these studies, in postmenopausal women phthalate exposure was associated with lower BMD and higher osteoporosis risk (Min and Min, 2014). In the present study, maternal LMW phthalate concentrations were associated with higher aBMC at 6 years and maternal DNOP concentrations were associated with lower aBMC at 10 years. However, these results should be carefully interpreted since they did not survive multiple testing correction.

Animal studies suggest that timing of EDC exposure could be an explanation for different findings in similar studies (Lejonklou et al., 2016; Lind et al., 2017). We explored if there is a critical exposure period during fetal development. We identified the first trimester to be the critical period for the association of BPS with BMD, BMC and aBMC. The fetal skeleton is being developed from first trimester onwards (Timor-Tritsch et al., 1992; van Zalen-Sprock et al., 1997). These findings suggest that exposure to BPS in the beginning of skeletal development may have persistent effects on bone development. Nevertheless, these results should be carefully interpreted since lower detection rates for BPS in second and third trimester might have precluded the detection of associations.

Results from previous studies on sex-specific associations of EDCs with bone health were not consistent. Fetal and neonatal exposure to BPA increased femoral BMC among male mice but decreased femoral mechanical strength among female mice (Pelch et al., 2012). Another study observed that in utero and lactational exposure to low-dose BPA elongated the femur among female rat offspring and increased cortical thickness among male rat offspring (Lejonklou et al., 2016). In the present study, we did not observe consistent differences in the associations of maternal bisphenol and phthalate metabolite concentrations with childhood bone health outcomes between boys and girls. This might be explained by the hypothesis that differences between boys and girls depend on the timing and nature of the EDC exposure.

Maternal dietary supplementation with methyl donors, such as folic acid, has been shown to limit the DNA hypomethylating effect of EDCs in early stem cell development (Dolinoy et al., 2007). Also, a higher folate intake is associated with better bone health (Tobias et al., 2005). Therefore, we hypothesized that folic acid supplementation may limit the adverse effects of fetal bisphenol and phthalate exposure on bone development. We observed that maternal phthalate metabolite concentrations tended to be inversely associated with bone measures in mothers who took folic acid supplementation and positively associated in those who did not take folic acid supplementation. These results do not suggest a protective effect of folic acid supplement use to prevent the adverse effects of bisphenols and phthalates. This might suggest that DNA hypomethylation is not among the main underlying mechanisms for the adverse effects of bisphenols and phthalates in bone development and thus no protective effect was observed when taking folic acid supplementation. The results observed for mothers who did not take folic acid supplementation should be carefully interpreted taking into account the small sample size and lack of statistical power.

The mechanisms by which EDC exposure affect bone health are not known yet. There is some evidence regarding the capacity of EDCs to interfere with the bone remodeling and homeostasis through the modulation of the signaling pathways involved (Agas et al., 2013). The EDCs activating or antagonizing these receptors can lead to an imbalance in hormonal production, which could affect bone formation (Agas et al., 2013). It is well established that young children and adolescents show a greater susceptibility to chemical toxicants than adults (Goldman, 1998). Further research addressing hormonal changes in response to specific EDCs in critical periods of life may help to disentangle underlying pathways.

Altogether, our results suggest that BPS, DNOP and LMW phthalate exposure during pregnancy may have persistent effects on bone health in childhood. Also, the first trimester of pregnancy might be a critical period. The observed effect estimates might be small on an individual level, but can be important on a population-based level. Small changes in bone quality during childhood may be related to bone related diseases such as osteoporosis in later life. Due to the observational design of this study, we cannot draw conclusions about causality. Further studies are needed to replicate these findings and investigate potential mechanisms.

### 4.2. Methodological considerations

An important strength of this study is the population-based cohort design from early life onwards, with measurements of maternal bisphenol and phthalate concentrations and bone mass in a large number of children. Of mothers with information on maternal bisphenol and phthalate concentrations during pregnancy, information on bone health was available for 97% of children at age 6 and 71% of children at age 10. Selection bias due to selective loss to follow-up is of concern if the associations between maternal bisphenol and phthalate concentrations and bone health differ between those included and not included in the study. As shown in the non-response analyses, mothers of children with and without follow-up data were different regarding the sociodemographic background, lifestyle characteristics and phthalate urinary concentrations. We cannot exclude the possibility of selection bias but we believe selection bias has little influence on our findings since we adjusted for most of these factors (Nohr and Liew, 2018). The lower urinary concentrations of phthalate metabolites observed in included mothers might have complicated the detection of associations due to low variability. We assessed associations of BPA analogues such as BPS, and of PA, a proxy for total phthalate exposure, with childhood bone health. Since our exposure measures were based on single spot urine sample in all three trimesters of pregnancy it may not accurately reflect preconceptional levels. Additionally, we had no information about neonatal exposure to bisphenols and phthalates, which is another important time period in early-life exposure to these chemicals and in bone development. Further research is needed to explore the associations of neonatal exposure to bisphenols and phthalates with childhood bone mass. Both bisphenols and phthalates are reported to have short biological half-lives (< 24 h) (Mattison et al., 2014; Braun et al., 2013). However, it has been suggested that a single urine sample for phthalate concentrations reasonably reflects exposure for up to 3 months (Hauser et al., 2004). Variability has been reported to be biomarker specific, with reasonable correlations for BPA and DEHP metabolites and stronger correlations for LMW phthalate metabolites and monobenzylphthalate (Braun et al., 2012; Mahalingaiah et al., 2008). In our study, we observed high variability for bisphenols and moderate variability for phthalates across pregnancy. A previous study has reported that between two and four weekly pools of 20 urines would be needed to properly classify women in terms of phthalate metabolites and bisphenol levels (Casas et al., 2018). Thus, by relying on a single-spot urinary measurement of phthalate metabolites and bisphenols as an estimate of exposure per trimester, our analyses may be affected by measurement error, which may have led to underestimation of the effect estimates, especially for bisphenols. Finally, we collected information on many potential confounding variables. However, as in any observational study, residual confounding due to unmeasured lifestyle variables might still be an issue.

## 5. Conclusion

Our study suggests that bone health in school-aged children may be influenced by fetal endocrine disruptor exposure, specifically BPS, in first trimester. However, due to the observational design of our study, these findings should be carefully interpreted, considered as hypothesis generating and need further replication and exploration of underlying mechanisms.

## Supplementary Material

Supplementary Material

## Figures and Tables

**Table 1:** Characteristics of mothers and their children (n = 1,362).

Characteristics	Total group (n = 1,362)	Boys (n = 688)	Girls (n = 674)	p- value
Maternal characteristics				
Age, mean (SD) (years)	30.6 (4.8)	30.6 (4.8)	30.5 (4.8)	0.61
Pre-pregnancy BMI, median (95% range) (kg/m^2^)	22.7 (18.5, 34.9)	22.7 (18.4, 35.2)	22.6 (18.5, 34.9)	0.49
Parity, *n* (%)				0.42
Nulliparous	829 (61.2)	426 (62.2)	403 (60.1)	
Multiparous	525 (38.8)	258 (37.8)	267 (39.9)	
Ethnicity, *n* (%)				0.64
European	839 (62.2)	429 (62.8)	410 (61.6)	
Non-European	510 (37.8)	254 (37.2)	256 (38.4)	
Education, *n* (%)				0.35
Lower	99 (7.6)	44 (6.7)	55 (8.5)	
Middle	549 (42.1)	273 (41.6)	276 (42.7)	
Higher	655 (50.3)	340 (51.8)	315 (48.8)	
Smoking during pregnancy, *n* (%)				0.92
Yes	300 (24.4)	150 (24.5)	150 (24.3)	
No	930 (75.6)	462 (75.5)	468 (75.7)	
Alcohol consumption during pregnancy, *n* (%)				0.06
Yes	706 (57.7)	368 (60.3)	338 (55.0)	
No	518 (42.3)	242 (39.7)	276 (45.0)	
Folic acid supplementation, *n* (%)				0.52
Yes	879 (80.9)	453 (81.6)	426 (80.1)	
No	208 (19.1)	102 (18.4)	106 (19.9)	
Vitamin D, *n* (%)				0.58
Severely deficient	267 (20.6)	126 (19.2)	141 (22.0)	
Deficient	327 (25.2)	163 (24.8)	164 (25.5)	
Sufficient	318 (24.5)	165 (25.2)	153 (23.8)	
Optimal	386 (29.7)	202 (30.8)	184 (28.7)	
Child characteristics at age 6				
Age, mean (SD) (years)	5.9 (0.2)	5.9 (0.2)	5.9 (0.2)	0.75
Height, mean (SD) (cm)	117.8 (4.9)	118.1 (5.0)	117.4 (4.8)	0.02
Bone-free mass, mean (SD) (kg)	18.3 (3.2)	18.3 (3.2)	18.3 (3.2)	0.97
Bone mineral density, mean (SD) (mg/cm^2^)	536.4 (43.5)	537.4 (42.5)	535.5 (44.5)	0.49
Bone mineral content, mean (SD) (g)	506.4 (82.3)	508.0 (82.3)	504.7 (82.4)	0.53
Area-adjusted bone mineral content, mean (SD) (g)	506.6 (1.0)	506.6 (1.0)	506.6 (1.0)	0.73
Bone area, mean (SD) (cm^2^)	939.7 (95.9)	941.3 (98.5)	938.1 (93.3)	0.60
Child characteristics at age 10				
Age, mean (SD) (years)	9.7 (0.2)	9.7 (0.3)	9.7 (0.2)	0.63
Height, mean (SD) (cm)	141.0 (6.3)	141.0 (6.3)	141.0 (6.4)	0.98
Bone-free mass, mean (SD) (kg)	29.9 (6.7)	29.5 (6.4)	30.3 (7.0)	0.04
Bone mineral density, mean (SD) (mg/cm^2^)	677.9 (64.0)	679.2 (62.6)	676.7 (65.3)	0.53
Bone mineral content, mean (SD) (g)	921.7 (160.7)	925.1 (155.9)	918.3 (165.4)	0.51
Area-adjusted bone mineral content, mean (SD) (g)	921.7 (1.0)	921.7 (1.0)	921.7 (1.0)	0.89
Bone area, mean (SD) (cm^2^)	1351.7 (128.8)	1355.0 (126.9)	1348.5 (130.8)	0.43

Values are observed data and represent means (SD), medians (95% range), or number of subjects (valid %).

Differences in participant characteristics between boys and girls were evaluated using t tests for normally distributed variables, Mann-Whitney U tests for non-normally distributed variables, and χ^2^ tests for categorical variables.

SD standard deviation; BMI, body mass index.

**Table 2:** Urinary bisphenol and phthalate concentrations in three trimesters during pregnancy (n = 1,362).

Phthalates and bisphenols	LOD	First trimester		Second trimester		Third trimester		
		Median (25th,75th percentile)	% below LOD	Median (25th,75th percentile)	% below LOD	Median (25th,75th percentile)	% below LOD	ICC
Low molecular weight phthalate		1087.27 (434.60, 2933.62)		593.31 (243.08, 1504.98)		1038.76 (411.97, 2624.78)		0.36
Monomethylphthalate	0.33	30.18 (15.23, 54.84)	0.1	19.33 (10.05, 34.93)	0.1	22.68 (11.15, 44.42)	0.5	0.30
Monoethylphthalate	0.31	713.67 (212.12, 2507.13)	0.1	378.90 (126.63, 1178.93)	0.0	684.15 (229.64, 2157.97)	0.0	0.40
Mono-isobutylphthalate	0.40	96.25 (43.05, 204.17)	0.1	40.75 (20.89, 81.69)	0.0	81.14 (41.56, 170.36)	0.3	0.27
Mono-n-butylphthalate	0.63	72.45 (30.95, 140.09)	0.7	43.65 (24.84, 86.86)	0.0	54.46 (27.65, 112.99)	0.1	0.23
High molecular weight phthalate		221.21 (112.89, 406.43)		134.21 (74.32, 250.43)		172.76 (94.83, 299.17)		0.21
Monobenzylphthalate	0.59	22.51 (8.97, 47.52)	8.2	20.99 (8.72, 44.43)	1.6	12.21 (4.47, 24.44)	3.5	0.25
Mono-hexylphthalate	0.24	0.88 (0.29, 1.98)	23.6	NA	> 80%	NA	> 80%	
Mono-2-heptylphthalate	1.14	2.12 (0.80, 5.63)	35.5	NA	> 80%	NA	> 80%	
DNOP								
Mono(3-carboxypropyl)phthalate	0.03	5.79 (3.09, 10.97)	0.2	3.54 (2.05, 6.80)	0.0	7.10 (3.79, 12.57)	0.1	0.23
DEHP		173.83 (89.47, 323.73)		98.66 (52.95, 188.07)		143.55 (77.49, 255.40)		0.19
Mono-(2-ethyl-5-carboxypentyl)phthalate	0.94	52.88 (26.69, 102.51)	0.1	34.29 (18.60, 66.64)	0.1	59.03 (30.69, 111.37)	0.0	0.26
Mono-[(2-carboxymethyl)hexyl]phthalate	0.13	45.59 (24.57, 86.22)	0.1	13.29 (7.26, 23.96)	0.2	11.30 (5.97, 21.14)	1.1	0.14
Mono-(2-ethyl-5-hydroxyhexyl)phthalate	0.27	40.99 (19.94, 78.97)	0.1	19.10 (10.24, 37.31)	0.1	35.23 (17.94, 68.78)	0.1	0.12
Mono-(2-ethyl-5oxohexyl)phthalate	0.14	26.74 (12.08, 52.73)	0.1	25.83 (12.49, 56.88)	0.0	25.09 (13.14, 48.60)	0.1	0.10
Phthalic Acid	6.68	343.12 (184.35, 725.15)	0.3	938.58 (372.72, 1477.13)	0.1	419.37 (204.51, 806.59)	0.4	0.20
Bisphenols		9.23 (3.53, 20.31)		6.34 (3.06, 13.98)		10.12 (4.67, 19.99)		0.06
Bisphenol A	0.66	4.92 (1.10, 12.31)	21.0	5.82 (2.69, 12.90)	7.0	6.57 (2.70, 13.29)	9.9	0.08
Bisphenol S	0.20	0.68 (0.13, 2.43)	32.2	0.13 (0.13, 0.40)	70.5	0.13 (0.13, 0.13)	80.7	0.01
Bisphenol F	0.90	0.62 (0.62, 2.08)	59.7	NA	> 80%	0.62 (0.62, 2.56)	70.9	

Absolute urinary concentrations of individual bisphenols and phthalates (in nmol/L urine) with concentrations below limit of detection imputed as limit of detection/square root of 2. Absolute urinary concentrations of grouped bisphenols and phthalates (in nmol/L urine). The limit of detection is expressed in nmol/L urine. Intraclass correlation coefficients between natural log-transformed phthalates or bisphenols across pregnancy were obtained using a single measurement, absolute agreement and two-way mixed effects model.

LOD, limit of detection; ICC, Intraclass correlation coefficients; NA, not applicable due to > 80% concentrations below limit of detection; DNOP, di-n-octylphthalate; DEHP, di-2-ethylhexylphthalate.

**Table 3:** Covariate-adjusted associations of maternal bisphenol concentrations with childhood bone mass.

Bisphenols	Age 6				Age 10			
	BMD (mg/cm^2^)		aBMC (g)		BMD (mg/cm^2^)		aBMC (g)	
	β (95% CI)	p-value	β (95% CI)	p-value	β (95% CI)	p-value	β (95% CI)	p-value
Total bisphenol								
First trimester	−1.12 (−3.38, 1.15)	0.33	−0.02 (−0.09, 0.04)	0.47	−2.90 (−6.41, 0.61)	0.11	−0.07 (−0.14, 0.01)	0.07
Second trimester	0.26 (−1.87, 2.38)	0.81	0.01 (−0.05, 0.07)	0.78	0.87 (−2.50, 4.24)	0.61	0.02 (−0.05, 0.09)	0.50
Third trimester	0.72 (−1.43, 2.87)	0.51	0.03 (−0.04, 0.09)	0.42	−0.86 (−4.21, 2.50)	0.62	−0.02 (−0.09, 0.05)	0.54
BPA								
First trimester	−0.54 (−2.83, 1.76)	0.65	−0.01 (−0.07, 0.06)	0.84	−1.31 (−4.86, 2.25)	0.47	−0.03 (−0.10, 0.05)	0.45
Second trimester	0.32 (−1.78, 2.41)	0.77	0.01 (−0.05, 0.07)	0.74	0.83 (−2.52, 4.17)	0.63	0.02 (−0.05, 0.09)	0.54
Third trimester	1.14 (−0.95, 3.23)	0.29	0.05 (−0.01, 0.11)	0.11	−1.20 (−4.46, 2.07)	0.47	−0.03 (−0.10, 0.04)	0.41
BPS								
First trimester	−2.04 (−4.55, 0.46)	0.11	−0.07 (−0.14, 0.00)	0.06	−6.08 (−9.97–−2.19)^†^	< 0.01	−0.12 (−0.20–−0.04)^†^	<0.01
Second trimester	−1.04 (−3.15, 1.07)	0.33	−0.01 (−0.07, 0.05)	0.81	−0.27 (−3.54, 3.00)	0.87	0.02 (−0.05, 0.08)	0.63
Third trimester	1.19 (−0.74, 3.11)	0.23	0.03 (−0.02, 0.09)	0.25	1.19 (−1.93, 4.32)	0.45	0.04 (−0.03, 0.10)	0.28
BPF								
First trimester	−0.55 (−2.95, 1.84)	0.65	−0.02 (−0.09, 0.05)	0.67	−1.56 (−5.37, 2.26)	0.42	−0.06 (−0.14, 0.02)	0.13
Second trimester	NA		NA		NA		NA	
Third trimester	−0.01 (−2.39, 2.37)	0.99	−0.01 (−0.08, 0.06)	0.81	1.23 (−2.54, 5.00)	0.52	0.05 (−0.03, 0.13)	0.23

Values are linear regression coefficients (β, 95% Confidence Interval) that reflect the differences in bone health for an interquartile range increase in each natural log-transformed bisphenol urinary concentrations in μmol/g. Confounder models include child’s age, sex, height and bonefree mass, maternal age, pre-pregnancy BMI, ethnicity and education level, parity, folic acid supplement use during pregnancy, alcohol and smoking habits (specifically during each trimester or during pregnancy) and vitamin D blood concentrations.

* p-value < 0.05

† significant after correction for multiple testing (p- value threshold of 0.0098).

BMD (mg/cm^2^), bone mineral density (milligram per square centimeter); aBMC (g), area adjusted bone mineral content (grams); CI, confidence interval; BPA, bisphenol A; BPS, bisphenol S, BPF, bisphenol F; NA, not applicable due to low detection rates.

**Table 4:** Covariate-adjusted associations of maternal phthalate concentrations with childhood bone mass.

Phthalates	BMD (mg/cm^2^)	Age 6		p-value	BMD (mg/cm^2^)	Age 10		p-value
		p-value	aBMC (g)			p-value	aBMC (g)	
	β (95% CI)		β (95% CI)		β (95% CI)		β (95% CI)	
Phthalic acid								
First trimester	−0.23 (−2.25, 1.80)	0.83	−0.01 (−0.07, 0.05)	0.70	−0.25 (−3.43, 2.93)	0.88	0.01 (−0.06, 0.07)	0.83
Second trimester	0.77 (−1.71, 3.24)	0.55	0.02 (−0.05, 0.09)	0.56	−0.62 (−4.48, 3.24)	0.75	−0.02 (−0.10, 0.07)	0.71
Third trimester	−0.95 (−3.19, 1.28)	0.40	−0.01 (−0.07, 0.06)	0.84	−2.76 (−6.23, 0.70)	0.12	−0.03 (−0.10, 0.04)	0.38
LMW phthalate metabolites								
First trimester	−0.61 (−2.89, 1.67)	0.60	−0.01 (−0.08, 0.06)	0.79	−0.92 (−4.55, 2.72)	0.62	0.01 (−0.07, 0.09)	0.81
Second trimester	0.92 (−1.44, 3.29)	0.44	0.04 (−0.03, 0.11)	0.28	−0.71 (−4.37, 2.95)	0.71	0.00 (−0.08, 0.08)	0.98
Third trimester	1.81 (−0.69, 4.30)	0.16	0.08 (0.00, 0.15)*	0.04	−0.50 (−4.36, 3.37)	0.80	0.02 (−0.06, 0.10)	0.69
HMW phthalate metabolites								
First trimester	−0.67 (−2.74, 1.41)	0.53	−0.03 (−0.09, 0.03)	0.27	−0.63 (−4.00, 2.75)	0.72	0.00 (−0.07, 0.07)	0.94
Second trimester	0.56 (−1.46, 2.58)	0.59	0.02 (−0.04, 0.08)	0.48	−0.02 (−3.23, 3.20)	0.99	−0.01 (−0.08, 0.05)	0.69
Third trimester	0.15 (−1.98, 2.28)	0.89	0.01 (−0.05, 0.07)	0.75	−0.04 (−3.37, 3.29)	0.98	−0.01 (−0.08, 0.06)	0.82
DEHP metabolites								
First trimester	−0.41 (−2.45, 1.63)	0.69	−0.03 (−0.09, 0.03)	0.33	−0.22 (−3.53, 3.09)	0.90	0.01 (−0.06, 0.08)	0.83
Second trimester	0.13 (−1.90, 2.17)	0.90	0.01 (−0.05, 0.07)	0.71	−0.52 (−3.75, 2.70)	0.75	−0.02 (−0.09, 0.05)	0.55
Third trimester	0.30 (−1.82, 2.41)	0.78	0.01 (−0.05, 0.07)	0.74	0.61 (−2.70, 3.91)	0.72	0.00 (−0.07, 0.07)	0.94
DNOP metabolites								
First trimester	−1.48 (−3.43, 0.47)	0.14	−0.06 (−0.11, 0.00)	0.05	−0.97 (−4.07, 2.14)	0.54	0.00 (−0.07, 0.06)	0.96
Second trimester	1.19 (−0.95, 3.34)	0.28	0.04 (−0.03, 0.10)	0.25	0.26 (−3.09, 3.61)	0.88	0.00 (−0.07, 0.07)	0.99
Third trimester	−1.39 (−3.45, 0.68)	0.19	−0.04 (−0.10, 0.03)	0.25	−3.02 (−6.24, 0.20)	0.07	−0.07 (−0.14, 0.00)*	0.04

Values are linear regression coefficients (β, 95% Confidence Interval) that reflect the differences in bone health for an interquartile range increase in each natural log-transformed phthalate urinary concentrations in μmol/g. Confounder models include child’s age, sex, height and bonefree mass, maternal age, pre-pregnancy BMI, ethnicity and education level, parity, folic acid supplement use during pregnancy, alcohol and smoking habits (specifically during each trimester or during pregnancy) and vitamin D blood concentrations.

* p-value < 0.05.

BMD (mg/cm^2^), bone mineral density (milligram per square centimeter); aBMC (g), area adjusted bone mineral content (grams); CI, confidence interval; LMW phthalate, low molecular weight phthalate; HMW phthalate, high molecular weight phthalate; DEHP, di-2-ethylhexylphthalate; DNOP, di-n-octylphthalate.
