# Supplementary material for: Fetal exposure to bisphenols and phthalates and childhood bone mass: a population-based prospective cohort study
Source: Environ Res. Author manuscript; Available in PMC 2021 Aug 4. (PMC8336628; doi:10.1016/j.envres.2020.109602)
Supplement: Supplementary Material [file NIHMS1718257-supplement-Supplementary_Material.docx]

**Supplemental Material**

**Fetal exposure to bisphenols and phthalates and childhood bone mass: a population-based prospective cohort study.**

Charissa van Zwol - Janssens, MD, Leonardo Trasande, MD, MPP, Alexandros G Asimakopoulos, PhD, Maria-Pilar Martinez-Moral, PhD, Kurunthachalam Kannan, PhD, Elise M Philips, MD, Fernando Rivadeneira, MD, PhD, Vincent WV Jaddoe, MD, PhD, Susana Santos, PhD

**Corresponding author:** Vincent WV Jaddoe MD PhD, The Generation R Study Group (Na 29-08), Erasmus MC, University Medical Center Rotterdam, PO Box 2040, 3000 CA, Rotterdam, The Netherlands. Phone: +31 10 7043405, fax: +31 10 70 44645, e-mail address: [v.jaddoe@erasmusmc.nl](mailto:v.jaddoe@erasmusmc.nl).

**CONTENTS**

**Figure S1.** Selection of study participants.

**Figure S2.** Directed acyclic graph depicting the relationship between exposure to bisphenols and phthalates, bone health and covariates

**Table S1.** Characteristics of mothers and their children, non-response analysis at age 10

**Table S2**. Urinary bisphenol and phthalate concentrations at three trimesters during pregnancy, non-response analysis at age 10

**Table S3.** Urinary bisphenol and phthalate concentrations at three trimesters during pregnancy, by sex.

**Table S4**. Covariate-adjusted associations of maternal bisphenol concentrations with childhood bone mass, overall mean values

**Table S5.** Covariate-adjusted associations of maternal bisphenol concentrations with childhood bone mass.

**Table S6.** Associations of maternal bisphenol concentrations with childhood bone mass at age 6.

**Table S7.** Associations of maternal bisphenol concentrations with childhood bone mass at age 10.

**Table S8.** Sex-specific associations of maternal bisphenol concentrations with childhood bone mass.

**Table S9.** Folic acid-dependent associations of maternal bisphenol concentrations with childhood bone mass.

**Table S10**. Covariate-adjusted associations of maternal phthalate concentrations with childhood bone mass, overall mean values

**Table S11.** Covariate-adjusted associations of maternal phthalate concentrations with childhood bone mass.

**Table S12.** Associations of maternal phthalate concentrations with childhood bone mass at age 6.

**Table S13.** Associations of maternal phthalate concentrations with childhood bone mass at age 10.

**Table S14.** Sex-specific associations of maternal phthalate concentrations with childhood bone mass.

**Table S15.** Folic acid-dependent associations of maternal phthalate concentrations with childhood bone mass.

**Table S16.** Associations of maternal bisphenol concentrations during first, second and third trimester with childhood bone mass at age 6 (sensitivity analysis, mutually adjusted model).

**Table S17.** Associations of maternal bisphenol concentrations during first, second and third trimester with childhood bone mass at age 10 (sensitivity analysis, mutually adjusted model).

**Table S18.** Associations of maternal phthalate concentrations during first, second and third trimester with childhood bone mass at age 6 (sensitivity analysis, mutually adjusted model).

**Table S19.** Associations of maternal phthalate concentrations during first, second and third trimester with childhood bone mass at age 10 (sensitivity analysis, mutually adjusted model).

**Table S20**. Associations of maternal bisphenol concentrations with childhood bone mass (sensitivity analysis by adding creatinine as a covariate in the model).

**Table S21**. Associations of maternal phthalate concentrations with childhood bone mass (sensitivity analysis by adding creatinine as a covariate in the model).

**Table S22**. Associations of maternal bisphenol concentrations with childhood bone mass (sensitivity analysis by using bisphenol concentrations categorized as detected and undetected).

Mothers with information on exposure to

bisphenol and phthalate during pregnancy with

singleton live-born children

**N = 1,405**

N = 26 excluded, due to missing information on

bisphenol and phthalate for at least one trimester in pregnancy

First trimester: N = 9

Second trimester: N = 14

Third trimester: N= 13

Mothers with information on exposure to

bisphenol and phthalate at all trimesters in

pregnancy with singleton live-born children

**N= 1,379**

N = 17 excluded, due to no information on bone health at age 6 and 10. or major movement artifacts, scan errors or bone development disorder

Mothers with information on exposure to

bisphenol and phthalate in all trimesters

in pregnancy and their singleton live-born

children with information on bone health at

age 6 or 10

**N = 1,362**

Aged 6: N= 1,335

Aged 10: N = 982

**Figure S1**. Selection of study participants.

Potential confounders:

- Maternal ethnicity
- Maternal age
- Maternal pre-pregnancy BMI
- Parity
- Maternal education
- Alcohol and smoking habits during pregnancy
- Vitamin D blood concentrations

Outcome: Bone health

Exposure: Bisphenols and phthalate

Potential effect modifiers:

- Folic acid supplement use

- Child’s sex

- Child’s age

- Child’s height

- Child’s bonefree mass

**Figure S2.** Directed acyclic graph depicting the relationship between exposure to bisphenols and phthalates, bone health and covariates

**Table S1**. Characteristics of mothers and their children, non-response analysis at age 10

| Characteristics | Included age 10  (n= 982) | Lost to follow-up  (n=380) | p- value |
| --- | --- | --- | --- |
| Maternal characteristics |  |  |  |
| Age, mean (SD) (years) | 30.9 (4.6) | 29.5 (5.2) | <0.001 |
| Pre-pregnancy BMI, median (95% range) (kg/m^2^) | 22.6 (18.5, 34.6) | 22.8 (18.4, 36.6) | 0.56 |
| Parity, *n* (%) |  |  | 0.20 |
| Nulliparous | 608 (62.3) | 221 (58.5) |  |
| Multiparous | 368 (37.7) | 157 (41.5) |  |
| Ethnicity, *n* (%) |  |  | <0.001 |
| European | 639 (65.4) | 200 (53.8) |  |
| Non-European | 338 (34.6) | 172 (46.2) |  |
| Education, *n* (%) |  |  | <0.001 |
| Lower | 61 (6.4) | 38 (10.8) |  |
| Middle | 377 (39.7) | 172 (48.7) |  |
| Higher | 512 (53.9) | 143 (40.5) |  |
| Smoking during pregnancy, *n* (%) |  |  | 0.11 |
| Yes | 207 (23.2) | 93 (27.6) |  |
| No | 686 (76.8) | 244 (72.4) |  |
| Alcohol consumption during pregnancy, *n* (%) |  |  | 0.003 |
| Yes | 535 (60.2) | 171 (50.9) |  |
| No | 353 (39.8) | 165 (49.1) |  |
| Folic acid supplementation, *n* (%) |  |  | 0.001 |
| Yes | 655 (83.2) | 224 (74.7) |  |
| No | 132 (16.8) | 76 (25.3) |  |
| Vitamin D, *n* (%) |  |  | 0.02 |
| Severely deficient | 173 (18.4) | 94 (26.1) |  |
| Deficient | 241 (25.7) | 86 (23.9) |  |
| Sufficient | 233 (24.8) | 85 (23.6) |  |
| Optimal | 291 (31.0) | 95 (26.4) |  |
| Child characteristics at age 6 |  |  |  |
| Age, mean (SD) (years) | 5.9 (0.2) | 6.0 (0.3) | <0.001 |
| Height, mean (SD) (cm) | 117.6 (5.0) | 118.2 (4.9) | 0.06 |

Values are observed data and represent means (SD), medians (95% range), or number of subjects (valid %)

Differences in participant characteristics between boys and girls were evaluated using t tests for normally distributed variables, Mann-Whitney U tests for non-normally distributed variables, and χ2 tests for categorical variables.

SD standard deviation; BMI, body mass index.

**Table S2**. Urinary bisphenol and phthalate concentrations at three trimesters during pregnancy, non-response analysis at age 10

|  | First trimester | | | Second trimester | | | Third trimester | | |
| --- | --- | --- | --- | --- | --- | --- | --- | --- | --- |
| Phthalates and bisphenols | Included  (n = 982) | Lost to follow-up (n = 380) | p-value | Included  (n = 982) | Lost to follow-up (n = 380) | p-value | Included  (n = 982) | Lost to follow-up (n = 380) | p-value |
| Low molecular weight phthalate | 1086.28 (424.73, 2875.21) | 1090.95 (478.68, 3161.07) | 0.27 | 533.55 (216.33, 1440.68) | 721.96 (310.14, 1748.43) | <0.001 | 952.84 (389.01, 2421.27) | 1299.64 (474.61, 3152.54) | 0.01 |
| Monomethylphthalate | 29.50 (15.03, 55.31) | 31.44 (16.14, 52.72) | 0.62 | 18.77 (9.70, 34.53) | 20.81 (11.39, 35.74) | 0.12 | 21.46 (10.70, 45.14) | 24.67 (11.66, 41.98) | 0.19 |
| Monoethylphthalate | 700.57 (203.84 –2448.66) | 768.68 (249.14, 2644.54) | 0.17 | 336.55 (118.91, 1106.29) | 479.65 (159.33, 1398.44) | 0.002 | 635.22 (218.05, 2015.28) | 884.69 (261.05, 2495.50) | 0.02 |
| Mono-isobutylphthalate | 95.57 (42.46, 199.29) | 98.31 (44.86, 239.98) | 0.20 | 38.92 (19.67, 75.18) | 46.64 (23.99, 111.89) | <0.001 | 74.61 (40.15, 152.64) | 103.98 (47.61, 196.74) | <0.001 |
| Mono-n-butylphthalate | 70.90 (30.25, 137.19) | 75.72 (33.19, 165.14) | 0.15 | 41.24 (23.00, 77.63) | 60.54 (30.00, 109.86) | <0.001 | 53.14 (27.51, 106.64) | 58.35 (28.28, 122.91) | 0.08 |
| High molecular weight phthalate | 209.88 (112.89, 385.70) | 237.72 (112.13, 462.05) | 0.04 | 126.07 (69.53, 230.91) | 159.99 (88.06, 312.53) | <0.001 | 165.44 (96.04, 291.70) | 189.37 (92.60, 334.14) | 0.11 |
| Monobenzylphthalate | 21.26 (8.62, 45.55) | 25.55 (10.57, 53.72) | 0.03 | 19.45 (8.10, 39.30) | 26.89 (11.08, 60.17) | <0.001 | 12.04 (3.98, 24.59) | 12.84 (5.24, 28.37) | 0.07 |
| Mono-hexylphthalate | 0.89 (0.29, 1.90) | 0.88 (0.28, 2.14) | 0.64 | 0.16 (0.16, 0.16) | 0.16 (0.16, 0.16) | 0.39 | 0.16 (0.16, 0.16) | 0.16 (0.16, 0.16) | 0.32 |
| Mono-2-heptylphthalate | 2.06 (0.80, 5.31) | 2.27 (0.80, 6.53) | 0.19 | 0.80 (0.80, 0.80) | 0.80 (0.80, 0.80) | 0.61 | 0.80 (0.80, 0.80) | 0.80 (0.80, 0.80) | 0.44 |
| DNOP |  |  |  |  |  |  |  |  |  |
| Mono(3-carboxypropyl)phthalate | 5.64 (3.09, 10.41) | 6.42 (3.07, 12.61) | 0.07 | 3.45 (1.98, 6.38) | 4.10 (2.30, 7.89) | 0.001 | 7.04 (3.75, 12.24) | 7.38 (3.81, 13.87) | 0.23 |
| DEHP | 167.58 (87.65, 310.08) | 191.18 (96.06, 378.02) | 0.02 | 94.84 (50.12, 176.29) | 116.76 (63.27, 225.71) | <0.001 | 136.96 (77.69, 250.63) | 158.25 (72.92, 279.12) | 0.12 |
| Mono-(2-ethyl-5-carboxypentyl)phthalate | 51.25 (25.72, 98.76) | 56.50 (28.97, 122.27) | 0.01 | 32.81 (17.78, 60.80) | 40.99 (21.59, 80.14) | <0.001 | 57.78 (30.35, 108.31) | 64.35 (32.37, 117.46) | 0.05 |
| Mono-[(2-carboxymethyl)hexyl]phthalate | 44.02 (24.43, 80.42) | 49.66 (26.18, 97.08) | 0.02 | 12.38 (6.84, 23.08) | 16.35 (8.56, 27.38) | <0.001 | 10.82 (5.68, 20.58) | 12.20 (6.61, 23.30) | 0.02 |
| Mono-(2-ethyl-5-hydroxyhexyl)phthalate | 39.60 (19.40, 75.22) | 43.75 (20.61, 85.87) | 0.05 | 18.03 (9.59, 35.62) | 21.96 (11.80, 45.50) | <0.001 | 35.08 (17.98, 67.08) | 35.97 (17.76, 75.45) | 0.34 |
| Mono-(2-ethyl-5oxohexyl)phthalate | 25.98 (11.80, 50.68) | 29.43 (12.36, 61.34) | 0.07 | 23.85 (11.57, 53.87) | 30.00 (14.54, 64.15) | 0.001 | 24.64 (13.29, 47.40) | 26.82 (12.71, 51.97) | 0.26 |
| Phthalic Acid | 333.59 (179.07, 709.31) | 358.08 (194.41, 843.00) | 0.11 | 876.74 (354.39, 1631.14) | 1092.92 (410.01, 1944.48) | 0.002 | 398.12 (198.13, 794.55) | 461.68 (215.82, 855.52) | 0.12 |
| Bisphenols | 9.05 (3.32, 20.27) | 9.82 (3.78, 20.55) | 0.42 | 6.16 (2.99, 13.41) | 7.00 (3.25, 14.84) | 0.24 | 10.05 (4.65, 19.87) | 10.54 (4.77, 21.07) | 0.63 |
| Bisphenol A | 4.78 (1.00, 11.98) | 5.27 (1.38, 12.97) | 0.39 | 5.70 (2.64, 12.25) | 6.32 (2.92, 14.32) | 0.17 | 6.39 (2.72, 12.79) | 6.74 (2.67, 14.40) | 0.69 |
| Bisphenol S | 0.70 (0.13, 2.40) | 0.66 (0.13, 2.58) | 0.67 | 0.13 (0.13, 0.42) | 0.13 (0.13, 0.37) | 0.55 | 0.13 (0.13, 0.13) | 0.13 (0.13, 0.13) | 0.26 |
| Bisphenol F | 0.62 (0.62, 2.08) | 0.62 (0.62, 2.06) | 0.72 | 0.62 (0.62, 0.62) | 0.62 (0.62, 0.62) | 0.29 | 0.62 (0.62, 2.35) | 0.62 (0.62, 3.11) | 0.22 |

Absolute urinary concentrations of individual bisphenols and phthalates in medians and 25th,75th percentile (in nmol/L urine) with concentrations below limit of detection imputed as limit of detection/square root of 2. Absolute urinary concentrations of grouped bisphenols and phthalates (in nmol/L urine). Differences between included and excluded/ lost in follow-up were tested using Mann-Whitney U tests.

DNOP, di-n-octylphthalate; DEHP, di-2-ethylhexylphthalate.

**Table S3**. Urinary bisphenol and phthalate concentrations at three trimesters during pregnancy, by sex (n = 1,362)

|  | First trimester | |  | Second trimester | |  | Third trimester | |  |
| --- | --- | --- | --- | --- | --- | --- | --- | --- | --- |
| Phthalates and bisphenols | Boys (n = 688) | Girls (n = 674) | p-value | Boys (n = 688) | Girls (n = 674) | p-value | Boys (n = 688) | Girls (n = 674) | p-value |
| Low molecular weight phthalate | 1080.13 (470.11, 2764.64) | 1096.94 (410.52, 3074.36) | 0.68 | 590.74 (252.23, 1422.33) | 595.87 (228.51, 1612.26) | 0.92 | 969.57 (408.25, 2460.63) | 1154.76 (417.21, 2795.22) | 0.31 |
| Monomethylphthalate | 29.10 (15.09, 53.87) | 31.70 (15.31, 56.27) | 0.36 | 19.21 (10.83, 35.83) | 19.45 (9.55, 34.56) | 0.30 | 21.80 (10.77, 40.09) | 23.07 (11.31, 47.05) | 0.11 |
| Monoethylphthalate | 689.11 (210.83, 2390.58) | 742.40 (212.12, 2588.66) | 0.37 | 382.37 (129.01, 1082.63) | 372.07 (126.34, 1332.63) | 0.38 | 627.50 (217.87, 2034.37) | 766.24 (238.91, 2362.89) | 0.13 |
| Mono-isobutylphthalate | 99.77 (45.88, 213.47) | 92.77 (40.19, 194.07) | 0.19 | 40.86 (21.46, 88.37) | 40.28 (20.39, 79.14) | 0.34 | 78.20 (41.49, 168.80) | 84.73 (41.87, 174.84) | 0.33 |
| Mono-n-butylphthalate | 75.21 (31.07, 142.08) | 70.68 (30.84, 138.38) | 0.43 | 46.25 (26.19, 88.47) | 42.37 (22.88, 85.59) | 0.16 | 54.53 (27.75, 112.10) | 54.34 (27.59, 113.31) | 0.83 |
| High molecular weight phthalate | 224.19 (110.65, 428.72) | 215.66 (114.72, 385.02) | 0.77 | 138.45 (79.19, 253.21) | 131.23 (70.26, 244.44) | 0.35 | 170.62 (94.27, 287.63) | 178.31 (95.13, 311.00) | 0.25 |
| Monobenzylphthalate | 22.78 (8.82, 48.48) | 22.35 (9.16, 46.01) | 0.69 | 21.90 (8.62, 43.30) | 19.79 (8.81, 47.83) | 0.94 | 11.88 (4.61, 25.13) | 12.92 (4.14, 26.08) | 0.70 |
| Mono-hexylphthalate | 0.86 (0.27, 2.14) | 0.91 (0.32, 1.89) | 0.70 | 0.16 (0.16, 0.16) | 0.16 (0.16, 0.16) | 0.34 | 0.16 (0.16, 0.16) | 0.16 (0.16, 0.16) | 0.96 |
| Mono-2-heptylphthalate | 2.12 (0.80, 5.80) | 2.14 (0.80, 5.50) | 0.78 | 0.80 (0.80, 0.80) | 0.80 (0.80, 0.80) | 0.26 | 0.80 (0.80, 0.80) | 0.80 (0.80, 0.80) | 0.63 |
| DNOP |  |  |  |  |  |  |  |  |  |
| Mono(3-carboxypropyl)phthalate | 5.66 (3.11, 11.24) | 5.95 (3.03, 10.78) | 0.95 | 3.61 (2.17, 6.96) | 3.44 (1.97, 6.52) | 0.09 | 7.19 (3.78, 12.90) | 7.06 (3.79, 12.32) | 0.90 |
| DEHP | 176.64 (87.58, 340.80) | 171.52 (90.46, 308.83) | 0.72 | 100.11 (56.01, 193.07) | 96.23 (50.60, 183.59) | 0.34 | 140.48 (75.49, 242.37) | 146.54 (79.99, 267.72) | 0.19 |
| Mono-(2-ethyl-5-carboxypentyl)phthalate | 52.12 (26.17, 103.25) | 53.80 (26.83, 101.53) | 0.94 | 36.28 (18.79, 68.85) | 33.03 (18.15, 64.06) | 0.24 | 57.06 (29.58, 111.60) | 60.24 (31.81, 111.09) | 0.30 |
| Mono-[(2-carboxymethyl)hexyl]phthalate | 45.18 (24.66, 86.44) | 46.11 (24.52, 85.72) | 0.75 | 13.01 (7.48, 24.12) | 13.37 (7.07, 23.83) | 0.95 | 10.68 (5.77, 20.54) | 11.85 (6.13, 21.36) | 0.25 |
| Mono-(2-ethyl-5-hydroxyhexyl)phthalate | 41.80 (19.89, 82.80) | 40.67 (19.99, 73.44) | 0.37 | 20.45 (11.05, 39.19) | 18.21 (9.71, 36.43) | 0.15 | 33.86 (16.91, 66.29) | 39.10 (18.24, 72.93) | 0.10 |
| Mono-(2-ethyl-5oxohexyl)phthalate | 27.31 (11.94, 55.77) | 26.39 (12.17, 48.97) | 0.38 | 27.24 (12.84, 59.66) | 23.45 (12.24, 54.77) | 0.15 | 24.43 (13.02, 46.31) | 26.15 (13.19, 50.80) | 0.15 |
| Phthalic Acid | 345.23 (181.30, 717.33) | 335.19 (186.69, 738.81) | 0.57 | 994.63 (389.99, 1841.84) | 899.72 (356.26, 1657.48) | 0.23 | 407.51 (199.48, 818.39) | 429.60 (214.59, 805.62) | 0.53 |
| Bisphenols | 9.56 (3.51, 20.82) | 8.93 (3.59, 19.90) | 0.86 | 6.75 (3.16, 14.41) | 6.12 (2.96, 13.32) | 0.13 | 9.60 (4.30, 19.47) | 10.67 (4.86, 20.54) | 0.17 |
| Bisphenol A | 5.25 (1.26, 12.36) | 4.56 (1.05, 12.17) | 0.42 | 6.11 (2.73, 13.76) | 5.52 (2.66, 11.82) | 0.16 | 6.08 (2.63, 12.01) | 7.16 (2.78, 14.71) | 0.04 |
| Bisphenol S | 0.70 (0.13, 2.43) | 0.68 (0.13, 2.43) | 0.74 | 0.13 (0.13, 0.45) | 0.13 (0.13, 0.32) | 0.31 | 0.13 (0.13, 0.13) | 0.13 (0.13, 0.13) | 0.61 |
| Bisphenol F | 0.62 (0.62, 1.85) | 0.62 (0.62, 2.23) | 0.32 | 0.62 (0.62, 0.62) | 0.62 (0.62, 0.62) | 0.15 | 0.62 (0.62, 2.95) | 0.62 (0.62, 1.95) | 0.19 |
| Absolute urinary concentrations of individual bisphenols and phthalates in medians and 25th,75th percentile (in nmol/L urine) with concentrations below limit of detection imputed as limit of detection/square root of 2. Absolute urinary concentrations of grouped bisphenols and phthalates (in nmol/L urine). Differences between boys and girls were tested using Mann-Whitney U tests. DNOP, di-n-octylphthalate; DEHP, di-2-ethylhexylphthalate. | | | | | | | | | |

**Table S4**. Covariate-adjusted associations of maternal bisphenol concentrations with childhood bone mass, overall mean values

|  | Age 6 | | | | Age 10 | | | |
| --- | --- | --- | --- | --- | --- | --- | --- | --- |
| Bisphenols | BMD (mg/cm^2^) | | aBMC (g) | | BMD (mg/cm^2^) | | aBMC (g) | |
|  | β (95% CI) | p-value | β (95% CI) | p-value | β (95% CI) | p-value | β (95% CI) | p-value |
|  |  |  |  |  |  |  |  |  |
| Overall mean Total bisphenol | -0.87 (-2.98, 1.23) | 0.42 | -0.01 (-0.07, 0.06) | 0.84 | -1.93 (-5.18, 1.32) | 0.24 | -0.03 (-0.10, 0.04) | 0.39 |
| Overall mean BPA | -0.15 (-2.19, 1.90) | 0.89 | 0.01 (-0.05, 0.07) | 0.64 | -1.00 (-4.17, 2.16) | 0.53 | -0.01 (-0.08, 0.05) | 0.69 |
| Overall mean BPS | -0.68 (-3.03, 1.67) | 0.57 | -0.02 (-0.08, 0.05) | 0.66 | -3.30 (-6.98, 0.38) | 0.08 | -0.05 (-0.13, 0.03) | 0.21 |
| Overall mean BPF | -0.31 (-2.74, 2.12) | 0.80 | -0.01 (-0.08, 0.06) | 0.75 | -0.41 (-4.27, 3.45) | 0.84 | -0.01 (-0.09, 0.07) | 0.74 |

Values are linear regression coefficients (β, 95% Confidence Interval) that reflect the differences in bone health for an interquartile range increase in each natural log-transformed bisphenol urinary concentrations in µmol/g. Confounder models include child’s age, sex, height and bonefree mass, maternal age, pre-pregnancy BMI, ethnicity and education level, parity, folic acid supplement use during pregnancy, alcohol and smoking habits during each trimester and vitamin D blood concentrations.

BMD (mg/cm^2^), bone mineral density (milligram per square centimeter); aBMC (g), area adjusted bone mineral content (grams); CI, Confidence Interval; BPA, bisphenol A; BPS, bisphenol S, BPF, bisphenol F.

**Table S5**. Covariate-adjusted associations of maternal bisphenol concentrations with childhood bone mass

|  | Age 6 | | | | Age 10 | | | | |
| --- | --- | --- | --- | --- | --- | --- | --- | --- | --- |
| Bisphenols | BMC (g) | | BA (cm^2^) | | BMC (g) | | BA (cm^2^) | | |
|  | β (95% CI) | p-value | β (95% CI) | p-value | β (95% CI) | p-value | β (95% CI) | | p-value |
| Total bisphenol |  |  |  |  |  |  |  | |  |
| First trimester | -1.07 (-3.66, 1.52) | 0.42 | -0.28 (-3.25, 2.69) | 0.86 | -1.57 (-7.31, 4.17) | 0.59 | 2.64 (-1.39, 6.67) | | 0.20 |
| Second trimester | 0.67 (-1.76, 3.10) | 0.59 | 0.47 (-2.31, 3.26) | 0.74 | 0.35 (-5.15, 5.85) | 0.90 | -1.15 (-5.01, 2.71) | | 0.56 |
| Third trimester | 0.42 (-2.04, 2.89) | 0.74 | -0.62 (-3.44, 2.21) | 0.67 | -0.43 (-5.91, 5.04) | 0.88 | 0.93 (-2.91, 4.77) | | 0.64 |
| Overall mean | -1.64 (-4.05, 0.77) | 0.18 | -1.82 (-4.59, 0.94) | 0.20 | -2.23 (-7.54, 3.08) | 0.41 | -0.19 (-3.93, 3.55) | | 0.92 |
| BPA |  |  |  |  |  |  |  | |  |
| First trimester | -0.59 (-3.21, 2.04) | 0.66 | -0.44 (-3.45, 2.57) | 0.77 | 0.33 (-5.48, 6.14) | 0.91 | 2.02 (-2.07, 6.10) | | 0.33 |
| Second trimester | 0.80 (-1.61, 3.21) | 0.51 | 0.56 (-2.19, 3.32) | 0.69 | 0.56 (-4.90, 6.03) | 0.84 | -0.80 (-4.64, 3.03) | | 0.68 |
| Third trimester | 0.30 (-2.10, 2.69) | 0.81 | -1.82 (-4.56, 0.92) | 0.19 | -0.99 (-6.32, 4.34) | 0.72 | 0.86 (-2.88, 4.60) | | 0.65 |
| Overall mean | -0.83 (-3.17, 1.51) | 0.49 | -1.70 (-4.38, 0.98) | 0.21 | -0.84 (-6.01, 4.33) | 0.75 | 0.08 (-3.56, 3.83) | | 0.97 |
| BPS |  |  |  |  |  |  |  | |  |
| First trimester | -0.69 (-3.57, 2.18) | 0.64 | 2.18 (-1.10, 5.47) | 0.19 | -7.61 (-13.98, -1.23)* | 0.02 | 0.62 (-3.86, 5.11) | | 0.79 |
| Second trimester | -1.99 (-4.41, 0.42) | 0.11 | -2.22 (-4.98, 0.55) | 0.12 | -3.24 (-8.57, 2.09) | 0.23 | -3.89 (-7.62, -0.16)* | | 0.04 |
| Third trimester | 1.01 (-1.19, 3.22) | 0.37 | -0.17 (-2.69, 2.36) | 0.90 | 1.22 (-3.88, 6.31) | 0.64 | -1.08 (-4.65, 2.50) | | 0.56 |
| Overall mean | -1.06 (-3.75, 1.64) | 0.44 | -0.66 (-3.75, 2.43) | 0.68 | -5.58 (-11.60, 0.43) | 0.07 | -1.99 (-6.23, 2.26) | | 0.36 |
| BPF |  |  |  |  |  |  |  | |  |
| First trimester | 0.13 (-2.61, 2.88) | 0.92 | 0.85 (-2.29, 3.99) | 0.60 | 1.45 (-4.78, 7.68) | 0.65 | 5.03 (0.67, 9.39)* | | 0.02 |
| Second trimester | NA |  | NA |  | NA |  | NA |  |  |
| Third trimester | 0.78 (-1.95, 3.51) | 0.57 | 1.38 (-1.75, 4.50) | 0.39 | 0.86 (-5.29, 7.01) | 0.79 | -2.14 (-6.45, 2.18) | | 0.33 |
| Overall mean | 0.34 (-2.45, 3.12) | 0.81 | 0.94 (-2.25, 4.14) | 0.56 | 1.19 (-5.13, 7.50) | 0.71 | 1.88 (-2.57, 6.32) | | 0.41 |

Values are linear regression coefficients (β, 95% Confidence Interval) that reflect the differences in bone health for an interquartile range increase in each natural log-transformed bisphenol urinary concentrations in µmol/g. Confounder models include child’s age, sex, height and bonefree mass, maternal age, pre-pregnancy BMI, ethnicity and education level, parity, folic acid supplement use during pregnancy, alcohol and smoking habits during each trimester and vitamin D blood concentrations.

*p-value <0.05

BMC (g), bone mineral content (grams); BA (cm^2^), bone area (square centimeter); CI: Confidence Interval; BPA, bisphenol A; BPS, bisphenol S, BPF, bisphenol F; NA, not applicable due to low detection rates.

**Table S6**. Associations of maternal bisphenol concentrations with childhood bone mass at age 6

| Bisphenols | BMD (mg/cm^2^) | | BMC (g) | | aBMC (g) | | BA (cm^2^) | |
| --- | --- | --- | --- | --- | --- | --- | --- | --- |
|  | β (95% CI) | p-value | β (95% CI) | p-value | β (95% CI) | p-value | β (95% CI) | p-value |
| Total bisphenol |  |  |  |  |  |  |  |  |
| First trimester | -2.15 (-4.69, 0.40) | 0.10 | -2.79 (-6.06, 0.47) | 0.09 | -0.04 (-0.11, 0.03) | 0.22 | -1.61 (-4.89, 1.68) | 0.34 |
| Second trimester | -0.57 (-2.96, 1.81) | 0.64 | -0.61 (-3.68, 2.45) | 0.70 | -0.01 (-0.07, 0.06) | 0.80 | -0.40 (-3.49, 2.68) | 0.80 |
| Third trimester | -0.81 (-3.23, 1.62) | 0.51 | -1.97 (-5.08, 1.14) | 0.21 | -0.01 (-0.07, 0.06) | 0.82 | -2.18 (-5.30, 0.95) | 0.17 |
| Overall mean | -2.76 (-5.13, -0.39)* | 0.02 | -4.62 (-7.66, -1.58)† | <0.01 | -0.05 (-0.11, 0.02) | 0.17 | -3.87 (-6.93, -0.81)* | 0.01 |
| BPA |  |  |  |  |  |  |  |  |
| First trimester | -1.06 (-3.63, 1.52) | 0.42 | -1.55 (-4.85, 1.76) | 0.36 | -0.01 (-0.08, 0.06) | 0.70 | -1.36 (-4.69, 1.96) | 0.42 |
| Second trimester | -0.35 (-2.71, 2.01) | 0.77 | -0.22 (-3.25, 2.81) | 0.89 | 0.00 (-0.07, 0.06) | 0.92 | -0.13 (-3.18, 2.92) | 0.93 |
| Third trimester | 0.66 (-1.71, 3.02) | 0.59 | -0.35 (-3.38, 2.69) | 0.82 | 0.04 (-0.03, 0.10) | 0.25 | -2.14 (-5.19, 0.91) | 0.17 |
| Overall mean | -1.09 (-3.39, 1.22) | 0.36 | -2.33 (-5.29, 0.62) | 0.12 | 0.00 (-0.07, 0.06) | 0.90 | -2.80 (-5.78, 0.17) | 0.06 |
| BPS |  |  |  |  |  |  |  |  |
| First trimester | -3.07 (-5.87, -0.26)* | 0.03 | -2.27 (-5.88, 1.34) | 0.22 | -0.09 (-0.17, -0.02)* | 0.02 | 1.18 (-2.45, 4.82) | 0.52 |
| Second trimester | -2.45 (-4.83, -0.07)* | 0.04 | -4.16 (-7.21, -1.11)† | <0.01 | -0.04 (-0.10, 0.03) | 0.24 | -3.59 (-6.66, -0.51)* | 0.02 |
| Third trimester | -0.73 (-2.88, 1.42) | 0.50 | -2.05 (-4.80, 0.71) | 0.15 | -0.01 (-0.07, 0.05) | 0.74 | -2.18 (-4.96, 0.59) | 0.12 |
| Overall mean | -2.67 (-5.32, -0.03)* | 0.05 | -4.09 (-7.49, -0.70)* | 0.02 | -0.06 (-0.13, 0.01) | 0.11 | -2.57 (-5.99, 0.86) | 0.14 |
| BPF |  |  |  |  |  |  |  |  |
| First trimester | -1.84 (-4.51, 0.84) | 0.18 | -1.84 (-5.27, 1.58) | 0.29 | -0.05 (-0.12, 0.03) | 0.22 | -0.33 (-3.79, 3.12) | 0.85 |
| Second trimester | NA |  | NA |  | NA |  | NA |  |
| Third trimester | -2.42 (-5.08, 0.23) | 0.07 | -3.09 (-6.50, 0.31) | 0.08 | -0.06 (-0.14, 0.01) | 0.09 | -1.17 (-4.60, 2.26) | 0.50 |
| Overall mean | -3.03 (-5.72, -0.34)* | 0.03 | -3.97 (-7.43, -0.52)* | 0.02 | -0.07 (-0.15, 0.00) | 0.05 | -1.79 (-5.27, 1.70) | 0.31 |

Values are linear regression coefficients (β, 95% Confidence Interval) that reflect the change in bone health for an interquartile range increase in each natural log-transformed bisphenol urinary concentrations in µmol/g. Basic models include child’s age, sex and height.

*p-value <0.05 †significant after correction for multiple testing (p- value threshold of 0.0098)

BMD (mg/cm^2^), bone mineral density (milligram per square centimeter); BMC (g), bone mineral content (grams); aBMC (g), area adjusted bone mineral content (grams); BA (cm^2^), bone area (square centimeter); CI, Confidence Interval; BPA, bisphenol A; BPS, bisphenol S, BPF, bisphenol F; NA, not applicable due to low detection rates.

**Table S7**. Associations of maternal bisphenol concentrations with childhood bone mass at age 10

| Bisphenols | BMD (mg/cm^2^) | | BMC (g) | | aBMC (g) | | BA (cm^2^) | |
| --- | --- | --- | --- | --- | --- | --- | --- | --- |
|  | β (95% CI) | p-value | β (95% CI) | p-value | β (95% CI) | p-value | β (95% CI) | p-value |
| Total bisphenol |  |  |  |  |  |  |  |  |
| First trimester | -2.77 (-6.99, 1.46) | 0.20 | -1.44 (-9.08, 6.20) | 0.71 | -0.06 (-0.14, 0.02) | 0.13 | 2.47 (-2.14, 7.07) | 0.29 |
| Second trimester | 0.76 (-3.30, 4.81) | 0.71 | 0.42 (-6.92, 7.75) | 0.91 | 0.02 (-0.06, 0.10) | 0.58 | -0.96 (-5.38, 3.47) | 0.67 |
| Third trimester | -3.83 (-7.86, 0.19) | 0.06 | -6.72 (-13.99, 0.56) | 0.07 | -0.07 (-0.14, 0.01) | 0.08 | -1.85 (-6.25, 2.55) | 0.41 |
| Overall mean | -3.97 (-7.90, -0.05)* | 0.05 | -6.50 (-13.59, 0.60) | 0.07 | -0.06 (-0.14, 0.02) | 0.12 | -2.13 (-6.42, 2.15) | 0.33 |
| BPA |  |  |  |  |  |  |  |  |
| First trimester | -0.08 (-4.32, 4.17) | 0.97 | 2.58 (-5.10, 10.26) | 0.51 | -0.01 (-0.09, 0.08) | 0.91 | 2.58 (-2.06, 7.21) | 0.28 |
| Second trimester | 1.27 (-2.76, 5.30) | 0.54 | 1.79 (-5.50, 9.08) | 0.63 | 0.03 (-0.05, 0.11) | 0.48 | -0.11 (-4.51, 4.29) | 0.96 |
| Third trimester | -2.72 (-6.66, 1.21) | 0.18 | -4.05 (-11.17, 3.07) | 0.27 | -0.05 (-0.13, 0.02) | 0.18 | -0.45 (-4.75, 3.85) | 0.84 |
| Overall mean | -1.65 (-5.46, 2.17) | 0.40 | -2.21 (-9.11, 4.69) | 0.53 | -0.02 (-0.09, 0.05) | 0.58 | -0.69 (-4.86, 3.47) | 0.74 |
| BPS |  |  |  |  |  |  |  |  |
| First trimester | -6.75 (-11.42, -2.08)† | <0.01 | -8.95 (-17.42, -0.47)* | 0.04 | -0.13 (-0.22, -0.04)† | <0.01 | 0.14 (-4.98, 5.26) | 0.96 |
| Second trimester | -3.12 (-7.04, 0.80) | 0.12 | -8.96 (-16.05, -1.88)* | 0.01 | -0.03 (-0.11, 0.05) | 0.44 | -6.15 (-10.42, -1.88)† | <0.01 |
| Third trimester | -1.81 (-5.51, 1.89) | 0.34 | -5.17 (-11.87, 1.530 | 0.13 | -0.01 (-0.08, 0.06) | 0.71 | -3.76 (-7.80, 0.28) | 0.07 |
| Overall mean | -6.17 (-10.59, -1.75)† | <0.01 | -11.41 (-19.41, -3.42)† | <0.01 | -0.10 (-0.18, -0.01)* | 0.03 | -4.30 (-9.14, 0.53) | 0.08 |
| BPF |  |  |  |  |  |  |  |  |
| First trimester | -3.46 (-8.02, 1.11) | 0.14 | -2.51 (-10.77, 5.75) | 0.55 | -0.09 (-0.18, - 0.01)* | 0.04 | 3.45 (-1.53, 8.43) | 0.17 |
| Second trimester | NA |  | NA |  | NA |  | NA |  |
| Third trimester | -3.25 (-7.72, 1.22) | 0.15 | -8.70 (-16.77, -0.63)* | 0.03 | -0.03 (-0.11, 0.06) | 0.56 | -6.17 (-11.03, 1.30)* | 0.01 |
| Overall mean | -5.33 (-9.90, -0.76)* | 0.02 | -9.39 (-17.65, -1.12)* | 0.03 | -0.09 (-0.18, -0.01)* | 0.04 | -2.69 (-7.68, 2.31) | 0.29 |

Values are linear regression coefficients (β, 95% Confidence Interval) that reflect the differences in bone health for an interquartile range increase in each natural log-transformed bisphenol urinary concentrations in µmol/g. Basic models include child’s age, sex and height.

*p-value <0.05 †significant after correction for multiple testing (p- value threshold of 0.0098)

BMD (mg/cm^2^), bone mineral density (milligram per square centimeter); BMC (g), bone mineral content (grams); aBMC (g), area adjusted bone mineral content (grams); BA (cm^2^), bone area (square centimeter); CI, Confidence Interval; BPA, bisphenol A; BPS, bisphenol S, BPF, bisphenol F; NA, not applicable due to low detection rates.

**Table S8**. Sex-specific associations of maternal bisphenol concentrations with childhood bone mass

|  | |  | | Age 6 | | | | |  | Age 10 | | |  |
| --- | --- | --- | --- | --- | --- | --- | --- | --- | --- | --- | --- | --- | --- |
| Bisphenols | |  | | Boys (n = 673) | | | Girls (n = 662) | | P-value for interaction | Boys (n = 489) | | Girls (n = 493) | P-value for interaction |
| Bone mineral density (mg/cm^2^) | | | | |  | |  | |  |  | |  |  |
|  | Total bisphenol | |  | | | |  | |  |  | |  |  |
|  | First trimester | | -0.41 (-3.74, 2.91) | | | | -1.45 (-4.58, 1.69) | | 0.69 | -3.62 (-9.13, 1.88) | | -1.90 (-6.47, 2.67) | 0.57 |
|  | Second trimester | | -0.39 (-3.34, 2.56) | | | | 0.86 (-2.21, 3.93) | | 0.67 | 0.86 (-4.08, 5.80) | | 0.74 (-3.89, 5.36) | 0.86 |
|  | Third trimester | | 2.20 (-0.78, 5.17) | | | | -0.79 (-3.94, 2.36) | | 0.19 | 0.32 (-4.48, 5.11) | | -1.88 (-6.63, 2.88) | 0.57 |
|  | Overall mean | | 0.66 (-2.29, 3.62) | | | | -2.26 (-5.30, 0.78) | | 0.17 | -1.41 (-6.28, 3.46) | | -2.24 (-6.63, 2.16) | 0.94 |
|  | BPA | |  | | | |  | |  |  | |  |  |
|  | First trimester | | -0.32 (-3.72, 3.09) | | | | -0.53 (-3.67, 2.61) | | 0.92 | -2.44 (-8.07, 3.19) | | -0.26 (-4.84, 4.33) | 0.60 |
|  | Second trimester | | -0.12 (-3.04, 2.79) | | | | 0.74 (-2.30, 3.78) | | 0.78 | 1.04 (-3.86, 5.94) | | 0.55 (-4.07, 5.16) | 0.94 |
|  | Third trimester | | 2.53 (-0.39, 5.44) | | | | -0.37 (-3.41, 2.66) | | 0.20 | 0.11 (-4.56, 4.78) | | -2.35 (-7.00, 2.30) | 0.48 |
|  | Overall mean | | 0.90 (-1.99, 3.78) | | | | -1.05 (-3.97, 1.87) | | 0.33 | -0.22 (-4.96, 4.52) | | -1.68 (-5.94, 2.58) | 0.70 |
|  | BPS | |  | | | |  | |  |  | |  |  |
|  | First trimester | | -0.11 (-3.73, 3.51) | | | | -3.39 (-6.91, 0.13) | | 0.25 | -6.54 (-12.47, -0.62)* | | -4.81 (-10.00, 0.38) | 0.53 |
|  | Second trimester | | -2.00 (-4.96, 0.97) | | | | -0.39 (-3.42, 2.64) | | 0.56 | 0.16 (-4.60, 4.92) | | -0.93 (-5.49, 3.62) | 0.77 |
|  | Third trimester | | 2.10 (-0.59, 4.80) | | | | 0.51 (-2.28, 3.29) | | 0.41 | 2.12 (-2.61, 6.84) | | 0.35 (-3.87, 4.58) | 0.75 |
|  | Overall mean | | 1.15 (-2.20, 4.51) | | | | -2.26 (-5.60, 1.07) | | 0.17 | -3.72 (-9.31, 1.88) | | -2.78 (-7.68, 2.12) | 0.64 |
|  | BPF | |  | | | |  | |  |  | |  |  |
|  | First trimester | | -0.64 (-4.11, 2.83) | | | | -0.28 (-3.60, 3.04) | | 0.65 | -2.18 (-7.97, 3.61) | | -0.10 (-5.13, 4.93) | 0.40 |
|  | Second trimester | | NA | | | | NA | | NA | NA | | NA | NA |
|  | Third trimester | | 1.62 (-1.72, 4.95) | | | | -1.29 (-4.78, 2.20) | | 0.23 | 4.21 (-1.30, 9.73) | | -1.24 (-6.52, 4.05) | 0.19 |
|  | Overall mean | | 0.81 (-2.69, 4.30) | | | | -1.11 (-4.53, 2.32) | | 0.55 | 0.67 (-5.15, 6.50) | | -0.69 (-5.84, 4.45) | 0.91 |
| Bone mineral content (g) | | |  | | | |  | |  |  | |  |  |
|  | Total bisphenol | |  | | | |  | |  |  | |  |  |
|  | First trimester | | -1.02 (-4.79, 2.76) | | | | -0.50 (-4.11, 3.11) | | 0.85 | -3.56 (-12.51, 5.39) | | 0.88 (-6.66, 8.42) | 0.35 |
|  | Second trimester | | 0.65 (-2.70, 4.01) | | | | 0.53 (-3.00, 4.06) | | 0.78 | 0.93 (-7.07, 8.92) | | -0.36 (-7.99, 7.27) | 0.91 |
|  | Third trimester | | 1.88 (-1.57, 5.19) | | | | -0.90 (-4.53, 2.73) | | 0.25 | 2.74 (-5.00, 10.48) | | -3.10 (-10.94, 4.75) | 0.31 |
|  | Overall mean | | -0.52 (-3.88, 2.85) | | | | -2.34 (-5.84, 1.16) | | 0.40 | -0.46 (-8.36, 7.43) | | -3.09 (-10.33, 4.15) | 0.77 |
|  | BPA | |  | | | |  | |  |  | |  |  |
|  | First trimester | | -0.71 (-4.58, 3.15) | | | | -0.07 (-3.68, 3.55) | | 0.86 | -1.71 (-10.85, 7.44) | | 2.28 (-5.27, 9.83) | 0.45 |
|  | Second trimester | | 1.16 (-2.15, 4.47) | | | | 0.36 (-3.15, 3.86) | | 0.59 | 1.91 (-6.02, 9.83) | | -0.69 (-8.31, 6.92) | 0.74 |
|  | Third trimester | | 1.13 (-2.18, 4.45) | | | | -0.36 (-3.85, 3.13) | | 0.51 | 2.15 (-5.39, 9.69) | | -3.44 (-11.11, 4.23) | 0.31 |
|  | Overall mean | | 0.03 (-3.25, 3.31) | | | | -1.32 (-4.68, 2.05) | | 0.47 | 1.79 (-5.89, 9.47) | | -2.70 (-9.72, 4.33) | 0.47 |
|  | BPS | |  | | | |  | |  |  | |  |  |
|  | First trimester | | 0.08 (-4.04, 4.19) | | | | -0.62 (-4.69, 3.45) | | 0.93 | -9.47 (-19.09, 0.15) | | -4.35 (-12.93, 4.22) | 0.31 |
|  | Second trimester | | -3.60 (-6.96, -0.24)* | | | | -0.97 (-4.46, 2.52) | | 0.36 | -3.60 (-11.29, 4.09) | | -3.85 (-11.34, 3.63) | 0.93 |
|  | Third trimester | | 2.00 (-1.06, 5.05) | | | | -0.05 (-3.25, 3.16) | | 0.38 | 4.19 (-3.44, 11.81) | | -1.44 (-8.42, 5.53) | 0.36 |
|  | Overall mean | | -0.38 (-4.20, 3.43) | | | | -1.50 (-5.34, 2.35) | | 0.74 | -7.39 (-16.44, 1.67) | | -3.76 (-11.85, 4.32) | 0.42 |
|  | BPF | |  | | | |  | |  |  | |  |  |
|  | First trimester | | 1.05 (-2.89, 4.99) | | | | -0.37 (-4.20, 3.45) | | 0.83 | 2.46 (-6.93, 11.86) | | 2.14 (-6.15, 10.42) | 0.77 |
|  | Second trimester | | NA | | | | NA | | NA | NA | | NA | NA |
|  | Third trimester | | 3.51 (-0.27, 7.28) | | | | -2.01 (-6.02, 2.00) | | 0.06 | 7.49 (-1.40, 16.38) | | -5.20 (-13.90, 3.51) | 0.05 |
|  | Overall mean | | 2.73 (-1.24, 6.69) | | | | -1.67 (-5.61, 2.27) | | 0.19 | 4.88 (-4.55, 14.31) | | -1.08 (-9.56, 7.40) | 0.48 |
| Area adjusted bone mineral content (g) | | | | | |  |  | |  |  | |  |  |
|  | Total bisphenol | |  | | | |  |  | |  | |  |  |
|  | First trimester | | 0.00 (-0.09, 0.10) | | | | -0.05 (-0.14, 0.05) | | 0.55 | -0.08 (-0.19, 0.03) | | -0.05 (-0.15, 0.04) | 0.68 |
|  | Second trimester | | -0.01 (-0.10, 0.08) | | | | 0.03 (-0.06, 0.12) | | 0.58 | 0.03 (-0.07, 0.14) | | 0.01 (-0.09, 0.11) | 0.99 |
|  | Third trimester | | 0.07 (-0.02, 0.16) | | | | -0.02 (-0.11, 0.07) | | 0.19 | 0.00 (-0.10, 0.10) | | -0.04 (-0.14, 0.06) | 0.62 |
|  | Overall mean | | 0.04 (-0.05, 0.13) | | | | -0.05 (-0.14, 0.04) | | 0.16 | -0.03 (-0.13, 0.08) | | -0.04 (-0.13, 0.06) | 0.94 |
|  | BPA | |  | | | |  | |  |  | |  |  |
|  | First trimester | | 0.01 (-0.09, 0.10) | | | | -0.01 (-0.11, 0.08) | | 0.84 | -0.05 (-0.16, 0.07) | | -0.01 (-0.11, 0.08) | 0.71 |
|  | Second trimester | | -0.01 (-0.09, 0.08) | | | | 0.03 (-0.06, 0.11) | | 0.63 | 0.03 (-0.07, 0.13) | | 0.01 (-0.09, 0.11) | 0.96 |
|  | Third trimester | | 0.10 (0.01, 0.18)* | | | | 0.00 (-0.09, 0.08) | | 0.14 | -0.01 (-0.10, 0.09) | | -0.05 (-0.15, 0.05) | 0.56 |
|  | Overall mean | | 0.04 (-0.04, 0.13) | | | | -0.02 (-0.10, 0.07) | | 0.36 | -0.01 (-0.11, 0.09) | | -0.02 (-0.11, 0.07) | 0.94 |
|  | BPS | |  | | | |  | |  |  | |  |  |
|  | First trimester | | 0.00 (-0.11, 0.11) | | | | -0.12 (-0.23, -0.02)* | | 0.13 | -0.13 (-0.25, 0.00)* | | -0.11 (-0.22, 0.00)* | 0.73 |
|  | Second trimester | | -0.02 (-0.11, 0.07) | | | | 0.00 (-0.09, 0.09) | | 0.85 | 0.04 (-0.06, 0.14) | | -0.01 (-0.10, 0.09) | 0.53 |
|  | Third trimester | | 0.06 (-0.02, 0.14) | | | | 0.02 (-0.06, 0.10) | | 0.48 | 0.05 (-0.05, 0.14) | | 0.02 (-0.07, 0.11) | 0.86 |
|  | Overall mean | | 0.05 (-0.05, 0.15) | | | | -0.08 (-0.17, 0.02) | | 0.08 | -0.04 (-0.16, 0.07) | | -0.06 (-0.16, 0.05) | 0.99 |
|  | BPF | |  | | | |  | |  |  | |  |  |
|  | First trimester | | -0.03 (-0.14, 0.07) | | | | 0.01 (-0.09, 0.10) | | 0.39 | -0.11 (-0.23, 0.01) | | -0.01 (-0.11, 0.10) | 0.16 |
|  | Second trimester | | NA | | | | NA | | NA | NA | | NA | NA |
|  | Third trimester | | 0.03 (-0.07, 0.13) | | | | -0.03 (-0.13, 0.07) | | 0.36 | 0.09 (-0.03, 0.20) | | 0.02 (-0.09, 0.13) | 0.44 |
|  | Overall mean | | 0.01 (-0.09, 0.11) | | | | -0.02 (-0.12, 0.08) | | 0.78 | -0.02 (-0.14, 0.10) | | 0.00 (-0.11, 0.11) | 0.71 |
| Bone area (cm^2^) | | |  | | | |  | |  |  | |  |  |
|  | Total bisphenol | |  | | | |  | |  |  | |  |  |
|  | First trimester | | -1.45 (-5.79, 2.89) | | | | 1.37 (-2.75, 5.48) | | 0.42 | 1.69 (-4.55, 7.92) | | 3.96 (-1.41, 9.32) | 0.47 |
|  | Second trimester | | 1.29 (-2.58, 5.16) | | | | -0.54 (-4.55, 3.47) | | 0.39 | -1.24 (-6.81, 4.33) | | -0.89 (-6.31, 4.52) | 0.88 |
|  | Third trimester | | -0.76 (-4.65, 3.13) | | | | -0.26 (-4.37, 3.86) | | 0.95 | 2.55 (-2.83, 7.92) | | -0.12 (-5.70, 5.46) | 0.46 |
|  | Overall mean | | -2.43 (-6.30, 1.45) | | | | -0.66 (-4.65, 3.32) | | 0.66 | 1.21 (-4.32, 6.74) | | -0.59 (-5.74, 4.57) | 0.65 |
|  | BPA | |  | | | |  | |  |  | |  |  |
|  | First trimester | | -1.16 (-5.61, 3.30) | | | | 0.53 (-3.60, 4.65) | | 0.68 | 1.34 (-5.04, 7.71) | | 2.80 (-2.58, 8.17) | 0.59 |
|  | Second trimester | | 1.72 (-2.10, 5.55) | | | | -0.72 (-4.70, 3.26) | | 0.28 | -0.16 (-5.68, 5.37) | | -1.16 (-6.56, 4.24) | 0.63 |
|  | Third trimester | | -2.79 (-6.61, 1.02) | | | | -0.29 (-4.26, 3.68) | | 0.47 | 2.31 (-2.93, 7.55) | | 0.06 (-5.40, 5.52) | 0.52 |
|  | Overall mean | | -1.90 (-5.69, 1.88) | | | | -0.98 (-4.81, 2.85) | | 0.92 | 2.13 (-3.25, 7.51) | | -1.11 (-6.11, 3.90) | 0.40 |
|  | BPS | |  | | | |  | |  |  | |  |  |
|  | First trimester | | 0.10 (-4.63, 4.82) | | | | 4.73 (0.12, 9.34)* | | 0.16 | -0.71 (-7.44, 6.03) | | 2.71 (-3.41, 8.82) | 0.36 |
|  | Second trimester | | -3.67 (-7.55, 0.21) | | | | -1.24 (-5.20, 2.73) | | 0.40 | -5.71 (-11.04, -0.38)* | | -2.88 (-8.19, 2.44) | 0.55 |
|  | Third trimester | | -0.02 (-3.55, 3.510 | | | | -0.89 (-4.52, 2.75) | | 0.78 | 0.93 (-4.37, 6.24) | | -2.55 (-7.50, 2.40) | 0.33 |
|  | Overall mean | | -2.80 (-7.20, 1.59) | | | | 1.47 (-2.90, 5.85) | | 0.17 | -3.91 (-10.27, 2.45) | | 0.03 (-5.73, 5.78) | 0.32 |
|  | BPF | |  | | | |  | |  |  |  |  |  |
|  | First trimester | | 2.89 (-1.65, 7.42) | | | | -0.75 (-5.10, 3.60) | | 0.28 | 8.68 (2.17, 15.19)† | | 2.29 (-3.61, 8.20) | 0.24 |
|  | Second trimester | | NA | | | | NA | | NA | NA | | NA | NA |
|  | Third trimester | | 3.16 (-1.20, 7.51) | | | | -1.17 (-5.71, 3.38) | | 0.22 | 1.43 (-4.77, 7.62) | | -5.63 (-11.81, 0.54) | 0.11 |
|  | Overall mean | | 3.16 (-1.42, 7.73) | | | | -1.16 (-5.64, 3.32) | | 0.23 | 5.46 (-1.12, 12.04) | | -1.04 (-7.08, 5.00) | 0.20 |
| Values are linear regression coefficients ( β, 95% Confidence Interval) that reflect the differences in bone health for an interquartile range increase in each natural log-transformed bisphenol urinary concentrations in µmol/g. Models include child’s age, height and bonefree mass, maternal age, pre-pregnancy BMI, ethnicity and education level, parity, folic acid supplement use during pregnancy, alcohol and smoking habits during each trimester and vitamin D blood concentrations.  *p-value <0.05 †significant after correction for multiple testing (p- value threshold of 0.0098)  mg/cm^2^, milligram per square centimeter; BPA, bisphenol A; BPS, bisphenol S, BPF, bisphenol F; NA, not applicable due to low detection rates; g, grams; cm^2^, square centimeter. | | | | | | | | | | | | | |

**Table S9.** Folic acid-dependent associations of maternal bisphenol concentrations with childhood bone mass.

|  | |  | | Age 6 | | | |  | Age 10 | | |  |
| --- | --- | --- | --- | --- | --- | --- | --- | --- | --- | --- | --- | --- |
| Bisphenols | |  | | Folic acid supplementation  (n = 860) | | | No folic acid supplementation  (n = 206) | P-value for interaction | Folic acid supplementation  (n = 655) | No folic acid supplementation  (n = 132) | | P-value for interaction |
| Bone mineral density (mg/cm^2^) | | | | |  | |  |  |  |  | |  |
|  | Total bisphenol | |  | | | |  |  |  |  | |  |
|  | First trimester | | -1.56 (-4.06, 0.94) | | | | 0.50 (-5.53, 6.52) | 0.51 | -3.16 (-7.11, 0.79) | -2.47 (-13.15, 8.21) | | 0.88 |
|  | Second trimester | | 0.61 (-1.70, 2.91) | | | | -1.00 (-6.74, 4.75) | 0.55 | 0.66 (-3.00, 4.32) | 1.31 (-8.29, 10.91) | | 0.75 |
|  | Third trimester | | 1.55 (-0.85, 3.95) | | | | -1.93 (-7.38, 3.52) | 0.18 | -1.00 (-4.74, 2.75) | 0.65 (-9.34, 10.63) | | 0.84 |
|  | Overall mean | | -0.51 (-2.81, 1.78) | | | | -2.20 (-7.66, 3.26) | 0.48 | -1.93 (-5.52, 1.65) | -2.11 (-11.80, 7.58) | | 0.93 |
|  | BPA | |  | | | |  |  |  |  | |  |
|  | First trimester | | -0.42 (-2.92, 2.07) | | | | -1.45 (-7.49, 4.59) | 0.83 | -0.75 (-4.73, 3.22) | -4.48 (-14.91, 5.95) | | 0.51 |
|  | Second trimester | | 0.61 (-1.67, 2.89) | | | | -0.70 (-6.36, 4.96) | 0.62 | 0.50 (-3.15, 4.14) | 1.75 (-7.73, 11.24) | | 0.65 |
|  | Third trimester | | 1.93 (-0.42, 4.29) | | | | -1.59 (-7.10, 3.93) | 0.20 | -1.35 (-5.01, 2.30) | 0.56 (-9.29, 10.41) | | 0.80 |
|  | Overall mean | | 0.37 (-1.84, 2.57) | | | | -2.57 (-8.14, 3.01) | 0.30 | -0.77 (-4.23, 2.69) | -2.36 (-11.87, 7.15) | | 0.70 |
|  | BPS | |  | | | |  |  |  |  | |  |
|  | First trimester | | -2.60 (-5.49, 0.30) | | | | -0.29 (-6.72, 6.15) | 0.54 | -6.70 (-11.10, -2.29)† | -4.28 (-15.68, 7.12) | | 0.59 |
|  | Second trimester | | -0.37 (-2.71, 1.97) | | | | -3.73 (-9.50, 2.03) | 0.22 | 0.83 (-2.83, 4.50) | -4.99 (-15.46, 5.49) | | 0.27 |
|  | Third trimester | | 1.40 (-0.76, 3.56) | | | | 0.53 (-4.50, 5.55) | 0.61 | 0.56 (-2.94, 4.06) | 4.40 (-4.85, 13.64) | | 0.46 |
|  | Overall mean | | -0.77 (-3.51, 1.98) | | | | -0.21 (-6.01, 5.65) | 0.99 | -3.59 (-7.78, 0.61) | -2.25 (-12.21, 7.72) | | 0.79 |
|  | BPF | |  | | | |  |  |  |  | |  |
|  | First trimester | | -0.55 (-3.15, 2.05) | | | | -0.89 (-7.76, 5.99) | 0.97 | -2.65 (-6.86, 1.56) | 2.74 (-9.83, 15.31) | | 0.32 |
|  | Second trimester | | NA | | | | NA | NA | NA | NA | | NA |
|  | Third trimester | | 0.47 (-2.17, 3.12) | | | | -1.72 (-8.23, 4.79) | 0.44 | 1.07 (-3.14, 5.27) | 3.33 (-9.30, 15.96) | | 0.77 |
|  | Overall mean | | -0.29 (-2.97, 2.38) | | | | -0.57 (-7.06, 5.93) | 0.90 | -1.45 (-5.69, 2.79) | 3.61 (-8.36, 15.57) | | 0.30 |
| Bone mineral content (g) | | |  | | | |  |  |  |  | |  |
|  | Total bisphenol | |  | | | |  |  |  |  | |  |
|  | First trimester | | -1.00 (-3.93, 1.93) | | | | -0.88 (-7.59, 5.83) | 0.97 | -0.86 (-7.27, 5.56) | -5.53 (-23.38, 12.31) | | 0.66 |
|  | Second trimester | | 1.65 (-1.07, 4.36) | | | | -3.73 (-10.13, 2.67) | 0.14 | 0.09 (-5.83, 6.01) | -0.34 (-16.88, 16.20) | | 0.84 |
|  | Third trimester | | 1.40 (-1.41, 4.20) | | | | -2.59 (-8.67, 3.48) | 0.23 | -0.41 (-6.35, 5.54) | 1.43 (-15.18, 18.05) | | 0.80 |
|  | Overall mean | | -0.78 (-3.51, 1.95) | | | | -4.64 (-10.83, 1.56) | 0.25 | -1.50 (-7.27, 4.26) | -5.79 (-21.94, 10.35) | | 0.68 |
|  | BPA | |  | | | |  |  |  |  | |  |
|  | First trimester | | -0.23 (-3.17, 2.72) | | | | -2.36 (-9.18, 4.46) | 0.68 | 1.89 (-4.58, 8.35) | -8.21 (-25.79, 9.38) | | 0.31 |
|  | Second trimester | | -1.80 (-0.88, 4.48) | | | | -3.62 (-9.89, 2.65) | 0.13 | 0.35 (-5.54, 6.24) | -0.25 (-16.59, 16.09) | | 0.89 |
|  | Third trimester | | 1.14 (-1.61, 3.88) | | | | -2.44 (-8.47, 3.59) | 0.24 | -1.06 (-6.85, 4.74) | 1.13 (-15.24, 17.50) | | 0.83 |
|  | Overall mean | | 0.08 (-2.54, 2.71) | | | | -4.96 (-11.27, 1.34) | 0.16 | -0.20 (-5.73, 5.34) | -4.83 (-20.69, 11.02) | | 0.60 |
|  | BPS | |  | | | |  |  |  |  | |  |
|  | First trimester | | -0.03 (-3.37, 3.31) | | | | -2.06 (-8.87, 4.75) | 0.59 | -6.63 (-13.80, 0.54) | -10.80 (-29.82, 8.23) | | 0.72 |
|  | Second trimester | | -1.32 (-4.07, 1.43) | | | | -4.87 (-11.28, 1.55) | 0.30 | -1.74 (-7.53, 4.06) | -9.86 (-26.52, 6.80) | | 0.38 |
|  | Third trimester | | 1.54 (-0.97, 4.04) | | | | -0.69 (-6.26, 4.88) | 0.51 | 0.19 (-5.39, 5.78) | 6.59 (-8.82, 22.01) | | 0.35 |
|  | Overall mean | | -0.21 (-3.41, 2.99) | | | | -3.03 (-9.45, 3.39) | 0.42 | -4.46 (-11.22, 2.31) | -8.00 (-24.47, 8.48) | | 0.71 |
|  | BPF | |  | | | |  |  |  |  | |  |
|  | First trimester | | 0.90 (-2.12, 3.93) | | | | -2.13 (-9.79, 5.53) | 0.42 | 1.02 (-5.77, 7.80) | 2.50 (-18.84, 23.83) | | 0.74 |
|  | Second trimester | | NA | | | | NA | NA | NA | NA | | NA |
|  | Third trimester | | 1.90 (-1.17, 4.98) | | | | -2.99 (-9.87, 3.88) | 0.21 | 0.98 (-5.69, 7.65) | 3.85 (-16.34, 24.04) | | 0.66 |
|  | Overall mean | | 1.33 (-1.77, 4.44) | | | | -2.80 (-9.85, 4.26) | 0.31 | 0.66 (-6.22, 7.53) | 2.16 (-18.65, 22.97) | | 0.55 |
| Area adjusted bone mineral content (g) | | | | | |  |  |  |  |  | |  |
|  | Total bisphenol | |  | | | |  |  |  |  | |  |
|  | First trimester | | -0.05 (-0.12, 0.03) | | | | 0.06 (-0.12, 0.23) | 0.26 | -0.09 (-0.17, -0.01)* | 0.01 (-0.21, 0.24) | | 0.34 |
|  | Second trimester | | 0.02 (-0.05, 0.08) | | | | -0.01 (-0.18, 0.16) | 0.70 | 0.03 (-0.05, 0.10) | 0.02 (-0.17, 0.22) | | 0.93 |
|  | Third trimester | | 0.05 (-0.03, 0.12) | | | | -0.04 (-0.20, 0.12) | 0.24 | -0.02 (-0.10, 0.06) | 0.00 (-0.20, 0.21) | | 0.90 |
|  | Overall mean | | 0.00 (-0.07, 0.06) | | | | -0.01 (-0.17, 0.15) | 0.73 | -0.04 (-0.11, 0.04) | 0.01 (-0.18, 0.21) | | 0.80 |
|  | BPA | |  | | | |  |  |  |  | |  |
|  | First trimester | | -0.01 (-0.08, 0.06) | | | | 0.00 (-0.18, 0.17) | 0.85 | -0.03 (-0.11, 0.05) | -0.04 (-0.25, 0.17) | | 0.98 |
|  | Second trimester | | 0.02 (-0.05, 0.08) | | | | 0.00 (-0.16, 0.17) | 0.85 | 0.02 (-0.06, 0.10) | 0.04 (-0.15, 0.23) | | 0.70 |
|  | Third trimester | | 0.07 (0.00, 0.14) | | | | -0.01 (-0.18, 0.15) | 0.31 | -0.03 (-0.11, 0.05) | 0.00 (-0.20, 0.20) | | 0.98 |
|  | Overall mean | | 0.02 (-0.04, 0.09) | | | | -0.02 (-0.18 -0.15) | 0.59 | -0.01 (-0.08, 0.06) | -0.01 (-0.21, 0.18) | | 0.86 |
|  | BPS | |  | | | |  |  |  |  | |  |
|  | First trimester | | -0.10 (-0.18, -0.01)* | | | | 0.01 (-0.18, 0.20) | 0.35 | -0.15 (-0.24, -0.06)† | -0.03 (-0.27, 0.21) | | 0.29 |
|  | Second trimester | | 0.01 (-0.06, 0.08) | | | | -0.08 (-0.25, 0.08) | 0.19 | 0.04 (-0.04, 0.12) | -0.08 (-0.30, 0.14) | | 0.22 |
|  | Third trimester | | 0.04 (-0.03, 0.10) | | | | 0.04 (-0.11, 0.18) | 0.71 | 0.02 (-0.05, 0.10) | 0.10 (-0.08, 0.29) | | 0.63 |
|  | Overall mean | | -0.03 (-0.10, 0.05) | | | | 0.02 (-0.14, 0.19) | 0.85 | -0.07 (-0.16, 0.02) | 0.03 (-0.18, 0.23) | | 0.47 |
|  | BPF | |  | | | |  |  |  |  |  |  |
|  | First trimester | | -0.03 (-0.10, 0.05) | | | | 0.02 (-0.17, 0.22) | 0.56 | -0.10 (-0.19, -0.01)* | 0.11 (-0.16, 0.37) | | 0.11 |
|  | Second trimester | | NA | | | | NA | NA | NA | NA | | NA |
|  | Third trimester | | -0.01 (-0.08, 0.07) | | | | -0.03 (-0.22, 0.16) | 0.62 | 0.04 (-0.05, 0.13) | 0.09 (-0.16, 0.35) | | 0.97 |
|  | Overall mean | | -0.03 (-0.10, 0.05) | | | | 0.02 (-0.16, 0.21) | 0.68 | -0.05 (-0.14, 0.04) | 0.14 (-0.10, 0.38) | | 0.18 |
| Bone area (cm^2^) | | |  | | | |  |  |  |  | |  |
|  | Total bisphenol | |  | | | |  |  |  |  | |  |
|  | First trimester | | 0.80 (-2.59, 4.19) | | | | -3.70 (-11.07, 3.67) | 0.30 | 4.58 (0.09, 9.07)* | -5.78 (-17.42, 5.87) | | 0.09 |
|  | Second trimester | | 1.39 (-1.88, 4.66) | | | | -4.34 (-11.87, 3.18) | 0.25 | -1.53 (-5.90, 2.74) | -1.59 (-12.33, 9.15) | | 0.88 |
|  | Third trimester | | -0.21 (-3.56, 3.14) | | | | -1.72 (-8.54, 5.10) | 0.84 | 1.03 (-3.22, 5.28) | 1.19 (-9.23, 11.62) | | 0.63 |
|  | Overall mean | | -0.84 (-4.06, 2.39) | | | | -5.38 (-12.38, 1.62) | 0.36 | 0.97 (-3.15, 5.08) | -5.96 (-16.04, 4.11) | | 0.40 |
|  | BPA | |  | | | |  |  |  |  | |  |
|  | First trimester | | 0.19 (-3.25, 3.64) | | | | -2.91 (-10.67, 4.84) | 0.53 | 3.36 (-1.20, 7.92) | -4.72 (-15.69, 6.26) | | 0.17 |
|  | Second trimester | | 1.66 (-1.56, 4.88) | | | | -4.83 (-12.07, 2.41) | 0.16 | -0.85 (-5.09, 3.40) | -2.68 (-13.25, 7.89) | | 0.82 |
|  | Third trimester | | -1.54 (-4.77, 1.69) | | | | -2.54 (-9.01, 3.92) | 0.77 | 0.85 (-3.26, 4.96) | 1.20 (-9.23, 11.62) | | 0.80 |
|  | Overall mean | | -0.84 (-3.94, 2.26) | | | | -5.59 (-12.88, 1.70) | 0.30 | 0.56 (-3.39, 4.51) | -3.44 (-13.42, 6.54) | | 0.62 |
|  | BPS | |  | | | |  |  |  |  | |  |
|  | First trimester | | 4.24 (0.38, 8.10)* | | | | -3.15 (-10.39, 4.08) | 0.12 | 3.26 (-1.75, 8.27) | -7.55 (-19.69, 4.60) | | 0.08 |
|  | Second trimester | | -2.21 (-5.36, 0.93) | | | | -2.47 (-9.34, 4.40) | 0.94 | -3.93 (-7.98, 0.12) | -3.71 (-14.19, 6.77) | | 0.72 |
|  | Third trimester | | 0.43 (-2.53, 3.39) | | | | -2.47 (-8.99, 4.05) | 0.74 | -1.21 (-5.10, 2.69) | -0.43 (-9.70, 8.85) | | 0.46 |
|  | Overall mean | | 0.87 (-2.82, 4.57) | | | | -4.91 (-11.53, 1.71) | 0.28 | 0.38 (-4.42, 5.19) | -8.60 (-18.81, 1.61) | | 0.18 |
|  | BPF | |  | | | |  |  |  |  | |  |
|  | First trimester | | 2.42 (-1.06, 5.90) | | | | -3.70 (-11.44, 4.05) | 0.12 | 6.93 (2.19, 11.68)† | -4.37 (-16.39, 7.64) | | 0.11 |
|  | Second trimester | | NA | | | | NA | NA | NA | NA | | NA |
|  | Third trimester | | 2.67 (-0.92, 6.25) | | | | -2.33 (-9.92, 5.26) | 0.38 | -1.49 (-6.18, 3.19) | -2.26 (-14.63, 10.11) | | 0.57 |
|  | Overall mean | | 2.89 (-0.72, 6.50) | | | | -4.66 (-12.27, 2.95) | 0.12 | 3.47 (-1.42, 8.36) | -6.53 (-19.30, 6.24) | | 0.52 |
| Values are linear regression coefficients (β, 95% Confidence Interval) that reflect the differences in bone health for an interquartile range increase in each natural log-transformed bisphenol urinary concentrations in µmol/g. Models include child’s age, sex, height and bonefree mass, maternal age, pre-pregnancy BMI, ethnicity and education level, parity, alcohol and smoking habits during each trimester and vitamin D blood concentrations.  *p-value <0.05 †significant after correction for multiple testing (p- value threshold of 0.0098)  mg/cm^2^, milligram per square centimeter; BPA, bisphenol A; BPS, bisphenol S, BPF, bisphenol F; NA, not applicable due to low detection rates; g, grams; cm^2^, square centimeter. | | | | | | | | | | | | |

**Table S10**. Covariate-adjusted associations of maternal phthalate concentrations with childhood bone mass, overall mean values

|  | Age 6 | | | | Age 10 | | | |
| --- | --- | --- | --- | --- | --- | --- | --- | --- |
| Phthalates | BMD (mg/cm^2^) | | aBMC (g) | | BMD (mg/cm^2^) | | aBMC (g) | |
|  | β (95% CI) | p-value | β (95% CI) | p-value | β (95% CI) | p-value | β (95% CI) | p-value |
| Overall mean phthalic acid | -0.07 (-2.34, 2.19 | 0.95 | 0.00 (-0.07, 0.07) | 1.00 | -1.73 (-5.28, 1.82) | 0.34 | -0.03 (-0.10, 0.05) | 0.48 |
| Overall mean LMW phthalate metabolites | 1.20 (-1.26, 3.66) | 0.34 | 0.06 (-0.02, 0.13) | 0.13 | -1.28 (-5.15, 2.58) | 0.52 | 0.01 (-0.07, 0.09) | 0.86 |
| Overall mean HMW phthalate metabolites | -0.15 (-2.15, 1.85) | 0.88 | -0.01 (-0.07, 0.05) | 0.65 | -1.37 (-4.64, 1.90) | 0.41 | -0.03 (-0.10, 0.04) | 0.35 |
| Overall mean DEHP metabolites | -0.15 (-2.13, 1.82) | 0.88 | -0.02 (-0.07, 0.04) | 0.60 | -1.00 (-4.19, 2.20) | 0.54 | -0.03 (-0.09, 0.04) | 0.45 |
| Overall mean DNOP metabolites | -1.14 (-3.18, 0.90) | 0.27 | -0.04 (-0.10, 0.02) | 0.15 | -2.39 (-5.62, 0.85) | 0.15 | -0.05 (-0.12, 0.02) | 0.15 |

Values are linear regression coefficients (β, 95% Confidence Interval) that reflect the differences in bone health for an interquartile range increase in each natural log-transformed phthalate urinary concentrations in µmol/g. Confounder models include child’s age, sex, height and bonefree mass, maternal age, pre-pregnancy BMI, ethnicity and education level, parity, folic acid supplement use during pregnancy, alcohol and smoking habits (specifically during each trimester or during pregnancy) and vitamin D blood concentrations.

BMD (mg/cm^2^), bone mineral density (milligram per square centimeter); aBMC (g), area adjusted bone mineral content (grams); CI, Confidence Interval; LMW phthalate, low molecular weight phthalate; HMW phthalate, high molecular weight phthalate; DEHP, di-2-ethylhexylphthalate; DNOP, di-n-octylphthalate.

**Table S11**. Covariate-adjusted associations of maternal phthalate concentrations with childhood bone mass

|  | Age 6 | | | | | Age 10 | | | |
| --- | --- | --- | --- | --- | --- | --- | --- | --- | --- |
| Phthalates | BMC (g) | | | BA (cm^2^) | | BMC (g) | | BA (cm^2^) | |
|  | β (95% CI) | | p-value | β (95% CI) | p-value | β (95% CI) | p-value | β (95% CI) | p-value |
| Phthalic acid |  | |  |  |  |  |  |  |  |
| First trimester | 0.32 (-2.00, 2.64) | | 0.79 | 0.92 (-1.73, 3.58) | 0.50 | -1.10 (-6.30, 4.10) | 0.68 | -1.42 (-5.06, 2.23) | 0.45 |
| Second trimester | 0.79 (-2.05, 3.63) | | 0.58 | 0.06 (-3.19, 3.31) | 0.33 | -1.17 (-7.48, 5.13) | 0.72 | -0.12 (-4.54, 4.31) | 0.96 |
| Third trimester | -1.94 (-4.49, 0.62) | | 0.14 | -2.19 (-5.11, 0.72) | 0.14 | -5.45 (-11.13, 0.22) | 0.06 | -2.88 (-6.84, 1.09) | 0.16 |
| Overall mean | 0.00 (-2.59, 2.60) | | 0.99 | 0.00 (-2.97, 2.98) | 0.99 | -3.57 (-9.36, 2.23) | 0.23 | -1.56 (-5.63, 2.52) | 0.45 |
| LMW phthalate metabolites |  | |  |  |  |  |  |  |  |
| First trimester | -0.52 (-3.14, 2.09) | | 0.70 | -0.27 (-3.26, 2.73) | 0.86 | -1.88 (-7.83, 4.06) | 0.53 | -2.25 (-6.41, 1.92) | 0.29 |
| Second trimester | 0.52 (-2.19, 3.23) | | 0.71 | -1.04 (-4.14, 2.06) | 0.51 | -2.83 (-8.80, 3.15) | 0.35 | -2.43 (-6.62, 1.75) | 0.25 |
| Third trimester | 0.16 (-2.70, 3.02) | | 0.91 | -3.23 (-6.49, 0.04) | 0.05 | -1.81 (-8.13, 4.52) | 0.58 | -2.58 (-7.00, 1.84) | 0.25 |
| Overall mean | -0.05 (-2.87, 2.77) | | 0.97 | -2.52 (-5.74, 0.71) | 0.13 | -3.31 (-9.62, 3.01) | 0.31 | -3.37 (-7.81, 1.08) | 0.14 |
| HMW phthalate metabolites |  | |  |  |  |  |  |  |  |
| First trimester | 0.01 (-2.36, 2.39) | | 0.99 | 1.52 (-1.19, 4.24) | 0.27 | -2.16 (-7.67, 3.35) | 0.44 | -2.07 (-5.93, 1.79) | 0.29 |
| Second trimester | 0.07 (-2.25, 2.38) | | 0.96 | -0.85 (-3.50, 1.80) | 0.53 | 0.19 (-5.06, 5.44) | 0.94 | 0.98 (-2.70, 4.67) | 0.60 |
| Third trimester | -0.59 (-3.03, 1.85) | | 0.63 | -1.20 (-3.99, 1.59) | 0.40 | 0.79 (-4.64, 6.21) | 0.78 | 1.20 (-2.61, 5.00) | 0.54 |
| Overall mean | 0.22 (-2.07, 2.51) | | 0.85 | 0.88 (-1.74, 3.51) | 0.51 | -1.68 (-7.03, 3.66) | 0.54 | 0.47 (-3.30, 4.23) | 0.81 |
| DEHP metabolites |  | |  |  |  |  |  |  |  |
| First trimester | 0.43 (-1.91, 2.77) | | 0.72 | 1.86 (-0.82, 4.53) | 0.17 | -1.54 (-6.95, 3.87) | 0.58 | -1.82 (-5.61, 1.98) | 0.35 |
| Second trimester | -0.46 (-2.79, 1.88) | | 0.70 | -1.10 (-3.76, 1.57) | 0.42 | -0.69 (-5.95, 4.58) | 0.80 | 0.62 (-3.08, 4.31) | 0.74 |
| Third trimester | -0.04 (-2.47, 2.38) | | 0.97 | -0.52 (-3.29, 2.25) | 0.71 | 2.13 (-3.26, 7.52) | 0.44 | 1.72 (-2.06, 5.50) | 0.37 |
| Overall mean | 0.40 (-1.86, 2.66) | | 0.73 | 1.20 (-1.39, 3.79) | 0.36 | -0.96 (-6.19, 4.26) | 0.72 | 0.69 (-2.99, 4.37) | 0.72 |
| DNOP metabolites |  |  |  |  |  |  |  |  |  |
| First trimester | -0.24 (-2.47, 1.99) | | 0.83 | 2.18 (-0.38, 4.73) | 0.10 | -1.70 (-6.78, 3.37) | 0.51 | -1.41 (-4.97, 2.15) | 0.44 |
| Second trimester | 0.67 (-1.79, 3.13) | | 0.59 | -0.78 (-3.59, 2.04) | 0.59 | 0.61 (-4.86, 6.08) | 0.83 | 0.53 (-3.31, 4.37) | 0.79 |
| Third trimester | -2.11 (-4.47, 0.25) | | 0.08 | -1.14 (-3.85, 1.56) | 0.41 | -4.30 (-9.56, 0.96) | 0.11 | 0.45 (-3.25, 4.14) | 0.81 |
| Overall mean | -1.02 (-3.36, 1.32) | | 0.39 | 0.63 (-2.05, 3.31) | 0.65 | -3.22 (-8.51, 2.06) | 0.23 | 0.15 (-3.57, 3.87) | 0.94 |

Values are linear regression coefficients (β, 95% Confidence Interval) that reflect the differences in bone health for an interquartile range increase in each natural log-transformed phthalate urinary concentrations in µmol/g. Confounder models include child’s age, sex, height and bonefree mass, maternal age, pre-pregnancy BMI, ethnicity and education level, parity, folic acid supplement use during pregnancy, alcohol and smoking habits during each trimester and vitamin D blood concentrations.

BMC (g), bone mineral content (grams); BA (cm^2^), bone area (square centimeter); CI, Confidence Interval; LMW phthalate, low molecular weight phthalate; HMW phthalate, high molecular weight phthalate; DEHP, di-2-ethylhexylphthalate; DNOP, di-n-octylphthalate.

**Table S12**. Associations of maternal phthalate concentrations with childhood bone mass at age 6

| Phthalates | BMD (mg/cm^2^) | | BMC (g) | | aBMC (g) | | BA (cm^2^) | |
| --- | --- | --- | --- | --- | --- | --- | --- | --- |
|  | β (95% CI) | p-value | β (95% CI) | p-value | β (95% CI) | p-value | β (95% CI) | p-value |
| Phthalic acid |  |  |  |  |  |  |  |  |
| First trimester | 0.07 (-2.20, 2.34) | 0.95 | 0.71 (-2.20, 3.62) | 0.63 | 0.00 (-0.07, 0.06) | 0.92 | 1.05 (-1.89, 3.98) | 0.48 |
| Second trimester | 0.83 (-1.94, 3.60) | 0.56 | 0.69 (-2.87, 4.25) | 0.71 | 0.03 (-0.05, 0.10) | 0.49 | -0.33 (-3.91, 3.26) | 0.86 |
| Third trimester | -0.42 (-2.91, 2.07) | 0.74 | -1.29 (-4.49, 1.91) | 0.43 | 0.01 (-0.06, 0.08) | 0.81 | -2.03 (-5.25, 1.19) | 0.22 |
| Overall mean | 0.05 (-2.47, 2.57) | 0.97 | 0.02 (-3.22, 3.25) | 0.99 | 0.01 (-0.06, 0.08) | 0.83 | -0.33 (-3.58, 2.93) | 0.85 |
| LMW phthalate metabolites |  |  |  |  |  |  |  |  |
| First trimester | 0.89 (-1.67, 3.45) | 0.50 | 1.72 (-1.56, 5.00) | 0.30 | 0.03 (-0.04, 0.10) | 0.46 | 1.02 (-2.28, 4.32) | 0.54 |
| Second trimester | 2.19 (-0.44, 4.82) | 0.10 | 2.22 (-1.16, 5.59) | 0.20 | 0.07 (0.00, 0.14) | 0.06 | -0.27 (-3.67, 3.13) | 0.89 |
| Third trimester | 2.38 (-0.41, 5.18) | 0.10 | 0.73 (-2.86, 4.32) | 0.69 | 0.10 (0.02, 0.17)* | 0.02 | -3.32 (-6.92, 0.29) | 0.07 |
| Overall mean | 2.49 (-0.25, 5.23) | 0.08 | 1.80 (-1.72, 5.32) | 0.32 | 0.09 (0.01, 0.16) | 0.02 | -1.61 (-5.15, 1.93) | 0.37 |
| HMW phthalate metabolites |  |  |  |  |  |  |  |  |
| First trimester | -0.44 (-2.77, 1.88) | 0.71 | 0.45 (-2.53, 3.43) | 0.77 | -0.03 (-0.09, 0.04) | 0.39 | 1.82 (-1.17, 4.82) | 0.23 |
| Second trimester | 0.54 (-1.71, 2.79) | 0.64 | 0.08 (-2.81, 2.98) | 0.96 | 0.02 (-0.04, 0.08) | 0.51 | -0.83 (-3.73, 2.09) | 0.58 |
| Third trimester | -0.02 (-2.41, 2.37) | 0.99 | -0.65 (-3.72, 2.42) | 0.68 | 0.01 (-0.06, 0.07) | 0.88 | -1.07 (-4.16, 2.02) | 0.50 |
| Overall mean | -0.40 (-2.63, 1.84) | 0.73 | -0.01 (-2.88, 2.86) | 0.99 | -0.02 (-0.08, 0.04) | 0.56 | 0.80 (-2.09, 3.69) | 0.59 |
| DEHP metabolites |  |  |  |  |  |  |  |  |
| First trimester | -0.47 (-2.76, 1.83) | 0.69 | 0.45 (-2.49, 3.40) | 0.76 | -0.03 (-0.09, 0.03) | 0.35 | 1.93 (-1.03, 4.89) | 0.20 |
| Second trimester | -0.13 (-2.41, 2.15) | 0.91 | -0.78 (-3.70, 2.15) | 0.60 | 0.01 (-0.06, 0.07) | 0.86 | -1.25 (-4.19, 1.70) | 0.41 |
| Third trimester | -0.13 (-2.51, 2.25) | 0.91 | -0.51 (-3.56, 2.54) | 0.75 | 0.00 (-0.07, 0.07) | 1.00 | -0.65 (-3.72, 2.42) | 0.68 |
| Overall mean | -0.67 (-2.89, 1.55) | 0.55 | -0.23 (-3.07, 2.62) | 0.88 | -0.03 (-0.09, 0.03) | 0.39 | 0.91 (-1.95, 3.77) | 0.53 |
| DNOP metabolites |  |  |  |  |  |  |  |  |
| First trimester | -1.82 (-4.00, 0.37) | 0.10 | -0.48 (-3.29, 2.33) | 0.74 | -0.07 (-0.13, -0.01)* | 0.03 | 2.29 (-0.54, 5.11) | 0.11 |
| Second trimester | -0.27 (-2.68, 2.13) | 0.83 | -1.61 (-4.69, 1.48) | 0.31 | 0.01 (-0.06, 0.07) | 0.87 | -2.30 (-5.41, 4.45) | 0.15 |
| Third trimester | -2.72 (-5.04, -0.40)* | 0.02 | -4.09 (-7.07, -1.11)† | <0.01 | -0.07 (-0.13, 0.00)* | 0.05 | -2.33 (-5.33, 0.67) | 0.13 |
| Overall mean | -2.63 (-4.92, -0.35)* | 0.02 | -3.06 (-5.99, -0.12)* | 0.04 | -0.08 (-0.14, -0.02)* | 0.02 | -0.44 (-3.40, 2.52) | 0.77 |

Values are linear regression coefficients (β, 95% Confidence Interval) that reflect the differences in bone health for an interquartile range increase in each natural log-transformed phthalate urinary concentrations in µmol/g. Basic models include child’s age, sex and height.

*p-value <0.05 †significant after correction for multiple testing (p- value threshold of 0.0098)

BMD (mg/cm^2^), bone mineral density (milligram per square centimeter); BMC (g), bone mineral content (grams); aBMC (g), area adjusted bone mineral content (grams); BA (cm^2^), bone area (square centimeter); CI, Confidence Interval; LMW phthalate, low molecular weight phthalate; HMW phthalate, high molecular weight phthalate; DEHP, di-2-ethylhexylphthalate; DNOP, di-n-octylphthalate.

**Table S13**. Associations of maternal phthalate concentrations with childhood bone mass at age 10

| Phthalates | BMD (mg/cm^2^) | | BMC (g) | | aBMC (g) | | BA (cm^2^) | |
| --- | --- | --- | --- | --- | --- | --- | --- | --- |
|  | β (95% CI) | p-value | β (95% CI) | p-value | β (95% CI) | p-value | β (95% CI) | p-value |
| Phthalic acid |  |  |  |  |  |  |  |  |
| First trimester | 2.03 (-1.75, 5.81) | 0.29 | 3.81 (-3.02, 10.65) | 0.27 | 0.04 (-0.03, 0.12) | 0.25 | 0.81 (-3.32, 4.94) | 0.70 |
| Second trimester | 1.67 (-2.93, 6.28) | 0.48 | 3.65 (-4.69, 11.99) | 0.39 | 0.02 (-0.06, 0.11) | 0.60 | 1.79 (-3.24, 6.82) | 0.49 |
| Third trimester | -0.15 (-4.27, 3.98) | 0.95 | 0.07 (-7.38, 7.52) | 0.99 | 0.01 (-0.07, 0.09) | 0.84 | -0.42 (-4.92, 4.08) | 0.86 |
| Overall mean | 1.05 (-3.15, 5.25) | 0.62 | 2.27 (-5.32, 9.87) | 0.56 | 0.02 (-0.06, 0.10) | 0.63 | 0.83 (-3.75, 5.42) | 0.72 |
| LMW phthalate metabolites |  |  |  |  |  |  |  |  |
| First trimester | 3.24 (-1.07, 7.55) | 0.14 | 6.90 (-0.91, 14.70) | 0.08 | 0.08 (-0.01, 0.16) | 0.08 | 1.58 (-3.13, 6.30) | 0.51 |
| Second trimester | 2.62 (-1.74, 6.97) | 0.24 | 4.12 (-3.76, 12.00) | 0.30 | 0.05 (-0.03, 0.14) | 0.22 | 0.47 (-4.29, 5.22) | 0.85 |
| Third trimester | 2.21 (-2.38, 6.80) | 0.34 | 3.70 (-4.61, 12.00) | 0.38 | 0.06 (-0.03, 0.15) | 0.17 | -0.47 (-5.48, 4.54) | 0.85 |
| Overall mean | 3.01 (-1.58, 7.60) | 0.20 | 5.77 (-2.52, 14.06) | 0.17 | 0.08 (-0.01, 0.16) | 0.09 | 0.51 (-4.50, 5.52) | 0.84 |
| HMW phthalate metabolites |  |  |  |  |  |  |  |  |
| First trimester | 2.14 (-1.87, 6.14) | 0.30 | 4.18 (-3.08, 11.43) | 0.26 | 0.04 (-0.03, 0.12) | 0.26 | 1.01 (-3.36, 5.39) | 0.65 |
| Second trimester | 1.74 (-2.07, 5.55) | 0.37 | 4.20 (-2.69, 11.08) | 0.23 | 0.02 (-0.06, 0.09) | 0.69 | 2.82 (-1.34, 6.97) | 0.18 |
| Third trimester | -0.51 (-4.49, 3.46) | 0.80 | 0.31 (-6.89, 7.50) | 0.93 | -0.02 (-0.09, 0.06) | 0.66 | 1.31 (-3.03, 5.65) | 0.55 |
| Overall mean | 0.04 (-3.86, 3.94) | 0.98 | 1.75 (-5.31, 8.80) | 0.63 | -0.01 (-0.09, 0.06) | 0.79 | 2.16 (-2.10, 6.42) | 0.32 |
| DEHP metabolites |  |  |  |  |  |  |  |  |
| First trimester | 2.04 (-1.91, 5.99) | 0.31 | 3.76 (-3.39, 10.90) | 0.30 | 0.04 (-0.34, 0.12) | 0.29 | 0.83 (-3.48, 5.15) | 0.71 |
| Second trimester | 0.66 (-3.18, 4.50) | 0.74 | 2.12 (-4.81, 9.06) | 0.55 | 0.00 (-0.08, 0.07) | 0.97 | 1.98 (-2.21, 6.16) | 0.35 |
| Third trimester | 0.02 (-3.91, 3.96) | 0.99 | 1.42 (-5.70, 8.55) | 0.70 | -0.01 (-0.08, 0.07) | 0.83 | 1.77 (-2.53, 6.06) | 0.42 |
| Overall mean | -0.17 (-4.00, 3.66) | 0.93 | 1.25 (-5.68, 8.18) | 0.72 | -0.01 (-0.09, 0.06) | 0.73 | 1.91 (-2.28, 6.09) | 0.37 |
| DNOP metabolites |  |  |  |  |  |  |  |  |
| First trimester | 0.63 (-3.09, 4.34) | 0.74 | 2.26 (-4.46, 8.97) | 0.51 | 0.02 (-0.05, 0.09) | 0.56 | 0.73 (-3.32, 4.79) | 0.72 |
| Second trimester | 0.15 (-3.87, 4.17) | 0.94 | 0.66 (-6.60, 7.91) | 0.86 | 0.00 (-0.08, 0.08) | 0.97 | 0.67 (-3.72, 5.05) | 0.77 |
| Third trimester | -4.33 (-8.20, -0.46)* | 0.03 | -6.81 (-13.82, 0.20) | 0.06 | -0.09 (-0.17, -0.02)* | 0.02 | -0.43 (-4.67, 3.80) | 0.84 |
| Overall mean | -2.68 (-6.56, 1.21) | 0.18 | -3.21 (-10.24, 3.82) | 0.37 | -0.06 (-0.13, 0.02) | 0.13 | 0.60 (-3.65, 4.84) | 0.78 |

Values are linear regression coefficients (β, 95% Confidence Interval) that reflect the differences in bone health for an interquartile range increase in each natural log-transformed phthalate urinary concentrations in µmol/g. Basic models include child’s age, sex and height.

*p-value <0.05

BMD (mg/cm^2^), bone mineral density (milligram per square centimeter); BMC (g), bone mineral content (grams); aBMC (g), area adjusted bone mineral content (grams); BA (cm^2^), bone area (square centimeter); CI, Confidence Interval; LMW phthalate, low molecular weight phthalate; HMW phthalate, high molecular weight phthalate; DEHP, di-2-ethylhexylphthalate; DNOP, di-n-octylphthalate.

**Table S14**. Sex-specific associations of maternal phthalate concentrations with childhood bone mass

|  | |  | Age 6 | | | |  | Age 10 | |  |
| --- | --- | --- | --- | --- | --- | --- | --- | --- | --- | --- |
| Phthalates | |  | Boys (n = 673) | | | Girls (n = 662) | P-value for interaction | Boys  (n = 489) | Girls  (n = 493) | P-value for interaction |
| Bone mineral density (mg/cm^2^) | | | |  | |  |  |  |  |  |
|  | Phthalic acid | |  | | |  |  |  |  |  |
|  | First trimester | | 0.23 (-2.67, 3.13) | | | -0.69 (-3.55, 2.17) | 0.82 | -1.43 (-6.12, 3.26) | 0.68 (-3.67, 5.03) | 0.55 |
|  | Second trimester | | 0.07 (-3.45, 3.59) | | | 0.77 (-2.78, 4.33) | 0.95 | -2.02 (-7.63, 3.59) | -0.22 (-5.61, 5.18) | 0.72 |
|  | Third trimester | | -0.30 (-3.48, 2.89) | | | -1.16 (-4.34, 2.02) | 0.78 | -1.97 (-6.92, 2.99) | -3.30 (-8.22, 1.62) | 0.73 |
|  | Overall mean | | 0.21 (-3.03, 3.45) | | | -0.67 (-3.88, 2.54) | 0.78 | -3.14 (-8.27, 1.99) | -1.10 (-6.03, 3.84) | 0.54 |
|  | LMW phthalate metabolites | |  | | |  |  |  |  |  |
|  | First trimester | | 0.35 (-2.92, 3.61) | | | -1.54 (-4.80, 1.72) | 0.45 | -1.26 (-6.57, 4.06) | -0.09 (-5.15, 4.97) | 0.99 |
|  | Second trimester | | 0.46 (-2.94, 3.85) | | | 0.90 (-2.43, 4.22) | 0.89 | -2.98 (-8.38, 2.42) | 0.61 (-4.40, 5.62) | 0.52 |
|  | Third trimester | | 2.96 (-0.65, 6.56) | | | 1.15 (-2.35, 4.65) | 0.47 | 0.99 (-4.69, 6.67) | -1.51 (-6.86, 3.83) | 0.50 |
|  | Overall mean | | 2.17 (-1.42, 5.75) | | | 0.31 (-3.12, 3.73) | 0.49 | -1.92 (-7.68, 3.85) | -0.62 (-5.88, 4.64) | 0.87 |
|  | HMW phthalate metabolites | |  | | |  |  |  |  |  |
|  | First trimester | | -0.81 (-3.75, 2.13) | | | -0.46 (-3.42, 2.51) | 0.83 | -1.90 (-6.65, 2.85) | 0.94 (-3.90, 5.79) | 0.48 |
|  | Second trimester | | 1.44 (-1.54, 4.43) | | | -0.33 (-3.09, 2.43) | 0.26 | 0.97 (-3.82, 5.76) | -0.96 (-5.34, 3.43) | 0.55 |
|  | Third trimester | | 1.87 (-1.32, 5.06) | | | -1.15 (-4.03, 1.74) | 0.11 | 0.83 (-4.50, 6.16) | 0.00 (-4.25, 4.24) | 0.69 |
|  | Overall mean | | 0.28 (-2.75, 3.30) | | | -0.52 (-3.19, 2.15) | 0.62 | -2.16 (-7.16, 2.84) | -0.59 (-4.89, 3.71) | 0.75 |
|  | DEHP metabolites | |  | | |  |  |  |  |  |
|  | First trimester | | -0.43 (-3.34, 2.49) | | | -0.39 (-3.29, 2.51) | 0.96 | -1.30 (-6.00, 3.41) | 0.93 (-3.79, 5.65) | 0.57 |
|  | Second trimester | | 1.27 (-1.71, 4.25) | | | -0.99 (-3.80, 1.81) | 0.18 | 0.26 (-4.52, 5.03) | -1.26 (-5.68, 3.16) | 0.65 |
|  | Third trimester | | 2.24 (-0.93, 5.40) | | | -1.27 (-4.14, 1.60) | 0.07 | 1.65 (-3.64, 6.94) | 0.41 (-3.80, 4.62) | 0.58 |
|  | Overall mean | | 0.68 (-2.32, 3.68) | | | -0.86 (-3.49, 1.77) | 0.38 | -1.65 (-6.56, 3.25) | -0.42 (-4.61, 3.78) | 0.82 |
|  | DNOP metabolites | |  | | |  |  |  |  |  |
|  | First trimester | | -1.41 (-4.14, 1.33) | | | -1.43 (-4.24, 1.39) | 0.95 | -1.79 (-6.18, 2.61) | 0.44 (-4.00, 4.88) | 0.47 |
|  | Second trimester | | 2.10 (-0.90, 5.10) | | | -0.08 (-3.19, 3.03) | 0.27 | 1.95 (-2.84, 6.75) | -2.27 (-7.02, 2.49) | 0.30 |
|  | Third trimester | | -2.01 (-5.04, 1.01) | | | -0.49 (-3.36, 2.38) | 0.48 | -5.06 (-9.95, -0.17)* | -0.78 (-5.10, 3.54) | 0.15 |
|  | Overall mean | | -1.10 (-4.02, 1.81) | | | -1.06 (-3.96, 1.85) | 0.94 | -2.28 (-7.01, 2.45) | -2.39 (-6.87, 2.10) | 0.86 |
| Bone mineral content (g) | | |  | | |  |  |  |  |  |
|  | Phthalic acid | |  | | |  |  |  |  |  |
|  | First trimester | | 1.37 (-1.92, 4.66) | | | -0.75 (-4.04, 2.55) | 0.47 | -2.57 (-10.18, 5.03) | -0.07 (-7.23, 7.09) | 0.61 |
|  | Second trimester | | 0.30 (-3.69, 4.30) | | | 0.72 (-3.37, 4.81) | 0.81 | -2.99 (-12.07, 6.09) | -0.25 (-9.13, 8.64) | 0.73 |
|  | Third trimester | | -0.44 (-4.05, 3.18) | | | -2.78 (-6.44, 0.88) | 0.31 | -3.34 (-11.37, 4.70) | -7.42 (-15.53, 0.69) | 0.50 |
|  | Overall mean | | 0.77 (-2.91, 4.45) | | | -0.96 (-4.65, 2.74) | 0.49 | -4.11 (-12.43, 4.20) | -4.23 (-12.35, 3.90) | 0.87 |
|  | LMW phthalate metabolites | |  | | |  |  |  |  |  |
|  | First trimester | | 1.90 (-1.80, 5.60) | | | -2.65 (-6.40, 1.10) | 0.09 | -1.45 (-10.07, 7.17) | -1.36 (-9.69, 6.97) | 0.86 |
|  | Second trimester | | 0.23 (-3.63, 4.08) | | | 0.42 (-3.41, 4.25) | 0.79 | -7.52 (-16.25, 1.22) | 0.46 (-7.80, 8.72) | 0.28 |
|  | Third trimester | | 2.22 (-1.87, 6.31) | | | -1.33 (-5.35, 2.69) | 0.16 | 1.14 (-8.07, 10.36) | -4.05 (-12.86, 4.76) | 0.45 |
|  | Overall mean | | 2.18 (-1.91, 6.26) | | | -1.98 (-5.92, 1.96) | 0.13 | -3.13 (-12.49, 6.23) | -3.28 (-11.93, 5.37) | 0.97 |
|  | HMW phthalate metabolites | |  | | |  |  |  |  |  |
|  | First trimester | | 0.27 (-3.07, 3.61) | | | -0.03 (-3.44, 3.38) | 0.91 | -2.08 (-9.80, 5.64) | -1.87 (-9.85, 6.11) | 0.99 |
|  | Second trimester | | -0.55 (-3.94, 2.84) | | | 0.56 (-2.62, 3.74) | 0.80 | 0.86 (-6.88, 8.60) | 0.01 (-7.21, 7.24) | 0.92 |
|  | Third trimester | | 1.62 (-2.00, 5.24) | | | -2.28 (-5.59, 1.04) | 0.06 | 1.40 (-7.20, 10.00) | 1.71 (-5.28, 8.71) | 0.90 |
|  | Overall mean | | 0.38 (-3.05, 3.81) | | | 0.20 (-2.88, 3.28) | 0.84 | -2.44 (-10.54, 5.67) | -0.45 (-7.54, 6.65) | 0.75 |
|  | DEHP metabolites | |  | | |  |  |  |  |  |
|  | First trimester | | 0.65 (-2.65, 3.96) | | | 0.34 (-2.99, 3.68) | 0.89 | -1.52 (-9.15, 6.12) | -1.26 (-9.04, 6.52) | 0.99 |
|  | Second trimester | | -0.51 (-3.89, 2.87) | | | -0.46 (-3.68, 2.77) | 0.86 | -0.02 (-7.74, 7.69) | -0.77 (-8.05, 6.52) | 0.93 |
|  | Third trimester | | 2.32 (-1.27, 5.91) | | | -1.91 (-5.21, 1.38) | 0.05* | 3.07 (-5.46, 11.60) | 2.70 (-4.24, 9.64) | 0.76 |
|  | Overall mean | | 1.05 (-2.35, 4.45) | | | -0.06 (-3.09, 2.97) | 0.53 | -1.61 (-9.56, 6.34) | 0.13 (-6.79, 7.05) | 0.79 |
|  | DNOP metabolites | |  | | |  |  |  |  |  |
|  | First trimester | | 0.44 (-2.66, 3.55) | | | -0.82 (-4.07, 2.42) | 0.59 | -2.84 (-9.99, 4.30) | 0.48 (-6.83, 7.79) | 0.51 |
|  | Second trimester | | 1.75 (-1.65, 5.15) | | | -0.85 (-4.43, 2.74) | 0.28 | 2.44 (-5.32, 10.20) | -2.32 (-10.16, 5.52) | 0.50 |
|  | Third trimester | | -1.88 (-5.31, 1.54) | | | -1.90 (-5.20, 1.40) | 0.94 | -6.37 (-14.29, 1.54) | -1.63 (-8.77, 5.50) | 0.33 |
|  | Overall mean | | -0.10 (-3.42, 3.21) | | | -1.97 (-5.31, 1.38) | 0.43 | -2.41 (-10.09, 5.27) | -3.91 (-11.30, 3.48) | 0.90 |
| Area adjusted bone mineral content (g) | | | | |  |  |  |  |  |  |
|  | Phthalic acid | |  | | |  |  |  |  |  |
|  | First trimester | | -0.01 (-0.09, 0.08) | | | -0.02 (-0.10, 0.07) | 0.95 | -0.02 (-0.12, 0.08) | 0.03 (-0.06, 0.12) | 0.54 |
|  | Second trimester | | 0.02 (-0.08, 0.12) | | | 0.01 (-0.10, 0.11) | 0.67 | -0.03 (-0.14, 0.09) | -0.03 (-0.14, 0.08) | 0.98 |
|  | Third trimester | | 0.00 (-0.09, 0.10) | | | -0.01 (-0.10, 0.08) | 0.96 | -0.02 (-0.12, 0.09) | -0.05 (-0.15, 0.06) | 0.68 |
|  | Overall mean | | 0.01 (-0.08, 0.11) | | | -0.02 (-0.11, 0.07) | 0.76 | -0.06 (-0.16, 0.05) | -0.02 (-0.12, 0.09) | 0.62 |
|  | LMW phthalate metabolites | |  | | |  |  |  |  |  |
|  | First trimester | | -0.01 (-0.11, 0.09) | | | -0.01 (-0.10, 0.09) | 0.93 | -0.03 (-0.14, 0.08) | 0.06 (-0.05, 0.16) | 0.48 |
|  | Second trimester | | 0.02 (-0.08, 0.12) | | | 0.04 (-0.05, 0.14) | 0.97 | -0.03 (-0.15 -0.08) | 0.01 (-0.09, 0.12) | 0.77 |
|  | Third trimester | | 0.09 (-0.01, 0.20) | | | 0.07 (-0.03, 0.17) | 0.81 | 0.04 (-0.07, 0.16) | -0.01 (-0.12, 0.11) | 0.47 |
|  | Overall mean | | 0.06 (-0.04, 0.17) | | | 0.05 (-0.05, 0.15) | 0.97 | -0.03 (-0.15, 0.09) | 0.04 (-0.07, 0.15) | 0.60 |
|  | HMW phthalate metabolites | |  | | |  |  |  |  |  |
|  | First trimester | | -0.04 (-0.13, 0.05) | | | -0.03 (-0.11, 0.06) | 0.80 | -0.04 (-0.14, 0.06) | 0.05 (-0.05, 0.15) | 0.22 |
|  | Second trimester | | 0.07 (-0.02, 0.16) | | | -0.03 (-0.11, 0.05) | 0.06 | 0.03 (-0.07, 0.13) | -0.06 (-0.15, 0.03) | 0.21 |
|  | Third trimester | | 0.06 (-0.03, 0.15) | | | -0.03 (-0.11, 0.06) | 0.11 | 0.04 (-0.07, 0.15) | -0.03 (-0.12, 0.06) | 0.31 |
|  | Overall mean | | 0.01 (-0.08, 0.10) | | | -0.04 (-0.11, 0.04) | 0.38 | -0.04 (-0.14, 0.07) | -0.03 (-0.12, 0.06) | 0.98 |
|  | DEHP metabolites | |  | | |  |  |  |  |  |
|  | First trimester | | -0.03 (-0.12, 0.06) | | | -0.03 (-0.12, 0.05) | 1.00 | -0.03 (-0.13, 0.07) | 0.04 (-0.06, 0.14) | 0.34 |
|  | Second trimester | | 0.07 (-0.02, 0.15) | | | -0.04 (-0.12, 0.04) | 0.04* | 0.01 (-0.09, 0.11) | -0.06 (-0.15, 0.03) | 0.33 |
|  | Third trimester | | 0.07 (-0.03, 0.16) | | | -0.04 (-0.12, 0.05) | 0.06 | 0.05 (-0.06, 0.16) | -0.02 (-0.11, 0.06) | 0.27 |
|  | Overall mean | | 0.02 (-0.07, 0.11) | | | -0.04 (-0.12, 0.03) | 0.24 | -0.03 (-0.13, 0.07) | -0.03 (-0.12, 0.06) | 0.90 |
|  | DNOP metabolites | |  | | |  |  |  |  |  |
|  | First trimester | | -0.06 (-0.14, 0.02) | | | -0.05 (-0.13, 0.03) | 0.89 | -0.02 (-0.11, 0.07) | 0.02 (-0.07, 0.12) | 0.47 |
|  | Second trimester | | 0.06 (-0.03, 0.15) | | | 0.01 (-0.08, 0.10) | 0.35 | 0.05 (-0.05, 0.15) | -0.08 (-0.17, 0.02) | 0.12 |
|  | Third trimester | | -0.06 (-0.14, 0.03) | | | -0.01 (-0.09, 0.07) | 0.45 | -0.11 (-0.21, -0.01)* | -0.03 (-0.12, 0.06) | 0.21 |
|  | Overall mean | | -0.05 (-0.13, 0.04) | | | -0.03 (-0.12, 0.05) | 0.74 | -0.05 (-0.15, 0.05) | -0.05 (-0.15, 0.04) | 0.84 |
| Bone area (cm^2^) | | |  | | |  |  |  |  |  |
|  | Phthalic acid | |  | | |  |  |  |  |  |
|  | First trimester | | 2.03 (-1.75, 5.81) | | | -0.20 (-3.95, 3.55) | 0.38 | -0.96 (-6.26, 4.34) | -1.83 (-6.93, 3.27) | 0.98 |
|  | Second trimester | | -0.45 (-5.06, 4.16) | | | 0.69 (-3.96, 5.34) | 0.88 | -1.04 (-7.37, 5.28) | 1.48 (-4.85, 7.81) | 0.64 |
|  | Third trimester | | -0.71 (-4.86, 3.44) | | | -3.23 (-7.37, 0.91) | 0.23 | -2.02 (-7.59, 3.56) | -3.77 (-9.51, 1.97) | 0.69 |
|  | Overall mean | | 0.45 (-3.79, 4.69) | | | -0.26 (-4.47, 3.96) | 0.63 | -0.33 (-6.16, 5.49) | -2.86 (-8.64, 2.92) | 0.73 |
|  | LMW phthalate metabolites | |  | | |  |  |  |  |  |
|  | First trimester | | 2.87 (-1.38, 7.12) | | | -3.02 (-7.28, 1.24) | 0.04* | 0.60 (-5.39, 6.60) | -4.64 (-10.56, 1.28) | 0.32 |
|  | Second trimester | | -0.69 (-5.13, 3.75) | | | -1.32 (-5.67, 3.03) | 0.74 | -4.57 (-10.64, 1.49) | -0.36 (-6.24, 5.53) | 0.29 |
|  | Third trimester | | -1.34 (-6.06, 3.37) | | | -4.86 (-9.41, -0.32)* | 0.18 | -1.66 (-8.05, 4.72) | -3.29 (-9.54, 2.95) | 0.86 |
|  | Overall mean | | 0.09 (-4.60, 4.79) | | | -4.75 (-9.22, -0.28)* | 0.10 | -1.09 (-7.65, 5.46) | -5.23 (-11.37, 0.92) | 0.53 |
|  | HMW phthalate metabolites | |  | | |  |  |  |  |  |
|  | First trimester | | 2.17 (-1.65, 6.00) | | | 1.20 (-2.68, 5.07) | 0.71 | 0.78 (-4.59, 6.15) | -4.85 (-10.53, 0.82) | 0.19 |
|  | Second trimester | | -3.86 (-7.77, 0.04) | | | 1.92 (-1.69, 5.53) | 0.03* | -1.00 (-6.41, 4.40) | 3.72 (-1.40, 8.84) | 0.22 |
|  | Third trimester | | -0.66 (-4.84, 3.52) | | | -1.65 (-5.41, 2.11) | 0.64 | -0.96 (-6.95, 5.03) | 3.41 (-1.56, 8.37) | 0.34 |
|  | Overall mean | | 0.06 (-3.90, 4.02) | | | 1.81 (-1.69, 5.30) | 0.53 | 0.17 (-5.51, 5.85) | 1.51 (-3.53, 6.56) | 0.67 |
|  | DEHP metabolites | |  | | |  |  |  |  |  |
|  | First trimester | | 2.17 (-1.62, 5.96) | | | 1.84 (-1.95, 5.63) | 0.88 | 0.47 (-4.85, 5.78) | -3.67 (-9.21, 1.87) | 0.31 |
|  | Second trimester | | -3.56 (-7.46, 0.33) | | | 1.20 (-2.47– 4.87) | 0.07 | -0.85 (-6.23, 4.53) | 2.96 (-2.21, 8.13) | 0.34 |
|  | Third trimester | | -0.03 (-4.17, 4.12) | | | -0.90 (-4.64, 2.84) | 0.68 | -0.21 (-6.14, 5.73) | 3.81 (-1.11, 8.73) | 0.40 |
|  | Overall mean | | 0.58 (-3.35, 4.51) | | | 1.90 (-1.54, 5.35) | 0.65 | 0.28 (-5.30, 5.85) | 1.92 (-3.00, 6.84) | 0.64 |
|  | DNOP metabolites | |  | | |  |  |  |  |  |
|  | First trimester | | 3.10 (-0.46, 6.66) | | | 1.27 (-2.42, 4.96) | 0.45 | -1.33 (-6.30, 3.63) | -0.97 (-6.19, 4.25) | 0.97 |
|  | Second trimester | | -0.49 (-4.43, 3.45) | | | -1.35 (-5.43, 2.72) | 0.77 | -1.12 (-6.52, 4.29) | 2.49 (-3.09, 8.06) | 0.39 |
|  | Third trimester | | 0.03 (-3.92, 3.97) | | | -2.05 (-5.80, 1.70) | 0.41 | 0.78 (-4.74, 6.29) | 0.51 (-4.56, 5.59) | 0.88 |
|  | Overall mean | | 2.04 (-1.76, 5.85) | | | -1.04 (-4.85, 2.77) | 0.23 | 0.77 (-4.59, 6.12) | -0.28 (-5.56, 4.99) | 0.70 |
| Values are linear regression coefficients (β, 95% Confidence Interval) that reflect the differences in bone health for an interquartile range increase in each natural log-transformed phthalate urinary concentrations in µmol/g. Models include child’s age, height and bonefree mass, maternal age, pre-pregnancy BMI, ethnicity and education level, parity, folic acid supplement use during pregnancy, alcohol and smoking habits during each trimester and vitamin D blood concentrations.  *p-value <0.05  mg/cm^2^, milligram per square centimeter; LMW phthalate, low molecular weight phthalate; HMW phthalate, high molecular weight phthalate; DEHP, di-2-ethylhexylphthalate; DNOP, di-n-octylphthalate; g, grams; cm^2^, square centimeter. | | | | | | | | | | |

**Table S15.** Folic acid-dependent associations of maternal phthalate concentrations with childhood bone mass

|  | |  | | Age 6 | | |  | Age 10 | |  |
| --- | --- | --- | --- | --- | --- | --- | --- | --- | --- | --- |
| Phthalates | |  | | Folic acid supplementation  (n = 860) | | No folic acid supplementation  (n = 206) | P-value for interaction | Folic acid supplementation  (n = 655) | No folic acid supplementation  (n = 132) | P-value for interaction |
| Bone mineral density (mg/cm^2^) | | | | |  |  |  |  |  |  |
|  | Phthalic acid | |  | | |  |  |  |  |  |
|  | First trimester | | -0.40 (-2.63, 1.83) | | | 0.49 (-5.19, 6.18) | 0.71 | -0.16 (-3.69, 3.36) | -1.09 (-11.91, 9.73) | 0.83 |
|  | Second trimester | | 0.12 (-2.62, 2.86) | | | 3.11 (-3.07, 9.29) | 0.31 | -0.62 (-4.82, 3.58) | -0.41 (-11.60, 10.78) | 1.00 |
|  | Third trimester | | -2.53 (-4.94, -0.12)* | | | 6.33 (0.11, 12.55)* | 0.01† | -4.43 (-8.17, -0.68)* | 6.99 (-3.20, 17.17) | 0.09 |
|  | Overall mean | | -1.36 (-3.85, 1.14) | | | 5.31 (-0.93, 11.54) | 0.04* | -2.50 (-6.29, 1.28) | 2.92 (-9.40, 15.25) | 0.55 |
|  | LMW phthalate metabolites | |  | | |  |  |  |  |  |
|  | First trimester | | -1.22 (-3.78, 1.34) | | | 1.63 (-4.72, 7.98) | 0.32 | -1.80 (-5.96, 2.37) | 3.32 (-8.64, 15.28) | 0.55 |
|  | Second trimester | | -0.17 (-2.76, 2.43) | | | 5.05 (-1.03, 11.13) | 0.07 | -1.67 (-5.66, 2.33) | 4.94 (-6.21, 16.09) | 0.34 |
|  | Third trimester | | -0.38 (-3.09, 2.34) | | | 10.64 (3.53, 17.74)† | <0.01† | -2.59 (-6.79, 1.60) | 10.80 (-0.55, 22.15) | 0.06 |
|  | Overall mean | | -0.77 (-3.50, 1.96) | | | 9.22 (2.26, 16.17)† | <0.01† | -3.05 (-7.30, 1.19) | 9.26 (-3.73, 22.25) | 0.14 |
|  | HMW phthalate metabolites | |  | | |  |  |  |  |  |
|  | First trimester | | -0.55 (-2.87, 1.77) | | | -1.17 (-6.23, 3.90) | 0.88 | -0.97 (-4.75, 2.80) | 0.59 (-9.43, 10.61) | 0.81 |
|  | Second trimester | | 0.15 (-2.05, 2.35) | | | 2.28 (-2.86, 7.43) | 0.42 | -0.88 (-4.36, 2.60) | 5.39 (-4.13, 14.92) | 0.20 |
|  | Third trimester | | -0.79 (-3.15, 1.58) | | | 3.18 (-2.16, 8.52) | 0.16 | -1.45 (-5.07, 2.17) | 6.32 (-2.75, 15.39) | 0.10 |
|  | Overall mean | | -0.76 (-2.97, 1.46) | | | 1.91 (-3.10, 6.92) | 0.29 | -2.93 (-6.49, 0.62) | 6.15 (-3.78, 16.07) | 0.09 |
|  | DEHP metabolites | |  | | |  |  |  |  |  |
|  | First trimester | | -0.19 (-2.49, 2.10) | | | -1.29 (-6.29, 3.72) | 0.74 | -0.37 (-4.09, 3.35) | -0.04 (-9.93, 9.86) | 0.97 |
|  | Second trimester | | -0.05 (-2.28, 2.19) | | | 0.77 (-4.28, 5.82) | 0.75 | -1.04 (-4.56, 2.48) | 2.77 (-6.80, 12.34) | 0.41 |
|  | Third trimester | | -0.57 (-2.92, 1.79) | | | 3.08 (-2.15, 8.30) | 0.20 | -0.91 (-4.50, 2.69) | 7.38 (-1.62, 16.37) | 0.08 |
|  | Overall mean | | -0.52 (-2.73, 1.69) | | | 1.00 (-3.93, 5.94) | 0.55 | -2.33 (-5.82, 1.17) | 5.02 (-4.75, 14.79) | 0.16 |
|  | DNOP metabolites | |  | | |  |  |  |  |  |
|  | First trimester | | -1.57 (-3.72, 0.59) | | | -1.37 (-6.48, 3.74) | 0.92 | -1.57 (-5.07, 1.93) | 1.16 (-8.38, 10.71) | 0.57 |
|  | Second trimester | | 0.26 (-2.08, 2.60) | | | 4.98 (-0.67, 10.64) | 0.10 | -0.59 (-4.18, 3.01) | 6.73 (-3.45, 16.91) | 0.15 |
|  | Third trimester | | -2.41 (-4.68, -0.14)* | | | 2.51 (-2.97, 7.99) | 0.10 | -3.49 (-6.99, 0.00) | -0.04 (-10.11, 10.04) | 0.49 |
|  | Overall mean | | -2.13 (-4.39, 0.13) | | | 2.11 (-2.96, 7.18) | 0.11 | -3.30 (-6.87, 0.27) | 1.95 (-7.66, 11.56) | 0.27 |
| Bone mineral content (g) | | |  | | |  |  |  |  |  |
|  | Phthalic acid | |  | | |  |  |  |  |  |
|  | First trimester | | -0.15 (-2.76, 2.47) | | | 2.65 (-3.75, 9.05) | 0.39 | -1.85 (-7.66, 3.96) | 2.41 (-16.30, 21.12) | 0.78 |
|  | Second trimester | | -0.23 (-3.45, 2.99) | | | 4.06 (-2.84, 10.96) | 0.23 | -0.94 (-7.73, 5.85) | -1.18 (-19.99, 17.63) | 0.80 |
|  | Third trimester | | -3.44 (-6.25, -0.64)* | | | 5.21 (-1.84, 12.25) | 0.02* | -8.34 (-14.42, -2.26)† | 11.74 (-6.11, 29.59) | 0.10 |
|  | Overall mean | | -1.36 (-4.27, 1.54) | | | 5.57 (-1.31, 12.45) | 0.06 | -4.76 (-10.91, 1.40) | 3.32 (-17.75, 24.38) | 0.68 |
|  | LMW phthalate metabolites | |  | | |  |  |  |  |  |
|  | First trimester | | -1.58 (-4.67, 1.51) | | | 4.14 (-3.40, 11.68) | 0.21 | -3.86 (-10.84, 3.11) | 7.22 (-14.29, 28.74) | 0.52 |
|  | Second trimester | | -1.29 (-4.31, 1.73) | | | 7.16 (0.62, 13.69)* | 0.02* | -4.93 (-11.31, 1.45) | 8.04 (-9.99, 26.08) | 0.27 |
|  | Third trimester | | -1.66 (-4.83, 1.51) | | | 8.46 (0.44, 16.47)* | 0.02* | -5.75 (-12.50, 1.01) | 20.47 (0.77, 40.16)* | 0.04* |
|  | Overall mean | | -2.21 (-5.41, 1.00) | | | 9.41 (1.45, 17.38)* | 0.01† | -6.55 (-13.57, 0.46) | 15.59 (-7.71, 38.89) | 0.19 |
|  | HMW phthalate metabolites | |  | | |  |  |  |  |  |
|  | First trimester | | 0.21 (-2.48, 2.89) | | | -0.37 (-5.87, 5.13) | 0.79 | -2.45 (-8.50, 3.60) | -1.12 (-17.89, 15.65) | 0.92 |
|  | Second trimester | | -0.31 (-2.91, 2.29) | | | 1.89 (-3.76, 7.54) | 0.56 | -0.42 (-6.03, 5.19) | 6.62 (-9.34, 22.58) | 0.58 |
|  | Third trimester | | -1.41 (-4.21, 1.38) | | | 1.92 (-4.18, 8.02) | 0.35 | -1.39 (-7.25, 4.47) | 10.36 (-5.37, 26.09) | 0.12 |
|  | Overall mean | | -0.22 (-2.80, 2.35) | | | 1.97 (-3.38, 7.32) | 0.52 | -3.36 (-9.08, 2.37) | 7.04 (-9.54, 23.61) | 0.32 |
|  | DEHP metabolites | |  | | |  |  |  |  |  |
|  | First trimester | | 0.66 (-1.99, 3.32) | | | -0.23 (-5.65, 5.20) | 0.73 | -1.76 (-7.70, 4.18) | -0.85 (-17.26, 15.56) | 0.94 |
|  | Second trimester | | -0.68 (-3.33, 1.97) | | | 0.63 (-4.98, 6.24) | 0.75 | -0.66 (-6.31, 5.00) | 1.87 (-14.10, 17.85) | 0.90 |
|  | Third trimester | | -0.94 (-3.71, 1.83) | | | 2.72 (-3.20, 8.63) | 0.29 | -0.27 (-6.07, 5.54) | 12.43 (-3.16, 28.01) | 0.08 |
|  | Overall mean | | 0.09 (-2.47, 2.65) | | | 1.54 (-3.68, 6.77) | 0.67 | -2.42 (-8.03, 3.19) | 6.29 (-9.84, 22.42) | 0.36 |
|  | DNOP metabolites | |  | | |  |  |  |  |  |
|  | First trimester | | -0.22 (-2.72, 2.29) | | | -0.32 (-5.89, 5.25) | 0.91 | -2.60 (-8.28, 3.08) | 1.44 (-14.41, 17.28) | 0.68 |
|  | Second trimester | | -0.39 (-3.16, 2.37) | | | 5.00 (-1.32, 11.33) | 0.10 | -0.33 (-6.13, 5.48) | 8.48 (-8.75, 25.72) | 0.32 |
|  | Third trimester | | -2.64 (-5.32, 0.05) | | | -0.24 (-6.43, 5.95) | 0.43 | -5.23 (-10.85, 0.39) | 2.98 (-13.72, 19.68) | 0.35 |
|  | Overall mean | | -1.65 (-4.29, 0.99) | | | 0.79 (-4.75, 6.33) | 0.36 | -4.37 (-10.10, 1.36) | 2.51 (-13.16, 18.17) | 0.38 |
| Area adjusted bone mineral content (g) | | | | |  |  |  |  |  |  |
|  | Phthalic acid | |  | | |  |  |  |  |  |
|  | First trimester | | -0.02 (-0.08, 0.05) | | | -0.01 (-0.17, 0.15) | 0.78 | 0.01 (-0.06, 0.08) | -0.04 (-0.26, 0.19) | 0.85 |
|  | Second trimester | | 0.01 (-0.07, 0.09) | | | 0.05 (-0.13, 0.23) | 0.60 | -0.01 (-0.10, 0.08) | -0.04 (-0.28, 0.19) | 0.94 |
|  | Third trimester | | -0.05 (-0.12, 0.02) | | | 0.22 (0.04, 0.40)* | <0.01† | -0.06 (-0.14, 0.02) | 0.12 (-0.08, 0.33) | 0.11 |
|  | Overall mean | | -0.03 (-0.10, 0.04) | | | 0.13 (-0.05, 0.31) | 0.08 | -0.04 (-0.12, 0.04) | 0.03 (-0.24, 0.30) | 0.57 |
|  | LMW phthalate metabolites | |  | | |  |  |  |  |  |
|  | First trimester | | -0.03 (-0.10, 0.05) | | | 0.04 (-0.13, 0.22) | 0.26 | -0.02 (-0.10, 0.07) | 0.10 (-0.13, 0.33) | 0.28 |
|  | Second trimester | | 0.01 (-0.06, 0.09) | | | 0.14 (-0.03, 0.32) | 0.10 | -0.02 (-0.11, 0.06) | 0.10 (-0.13, 0.33) | 0.23 |
|  | Third trimester | | 0.00 (-0.08, 0.08) | | | 0.36 (0.16, 0.56)† | <0.01† | -0.02 (-0.11, 0.06) | 0.19 (-0.04, 0.41) | 0.07 |
|  | Overall mean | | 0.00 (-0.08, 0.08) | | | 0.28 (0.08, 0.47)† | <0.01† | -0.04 (-0.12, 0.05) | 0.21 (-0.04, 0.46) | 0.05 |
|  | HMW phthalate metabolites | |  | | |  |  |  |  |  |
|  | First trimester | | -0.03 (-0.09, 0.04) | | | -0.07 (-0.22, 0.08) | 0.77 | -0.01 (-0.08, 0.07) | 0.02 (-0.19, 0.23) | 0.66 |
|  | Second trimester | | 0.02 (-0.05, 0.08) | | | 0.03 (-0.12, 0.18) | 0.70 | -0.03 (-0.10, 0.05) | 0.04 (-0.15, 0.24) | 0.27 |
|  | Third trimester | | -0.01 (-0.08, 0.06) | | | 0.07 (-0.09, 0.22) | 0.30 | -0.03 (-0.10, 0.05) | 0.08 (-0.10, 0.27) | 0.29 |
|  | Overall mean | | -0.02 (-0.09, 0.04) | | | 0.01 (-0.14, 0.15) | 0.56 | -0.06 (-0.14, 0.01) | 0.09 (-0.11, 0.30) | 0.11 |
|  | DEHP metabolites | |  | | |  |  |  |  |  |
|  | First trimester | | -0.02 (-0.08, 0.05) | | | -0.08 (-0.22, 0.07) | 0.57 | 0.01 (-0.07, 0.08) | 0.00 (-0.21, 0.20) | 0.91 |
|  | Second trimester | | 0.01 (-0.05, 0.08) | | | -0.01 (-0.16, 0.14) | 0.91 | -0.03 (-0.10, 0.05) | 0.00 (-0.20, 0.20) | 0.51 |
|  | Third trimester | | 0.00 (-0.07, 0.06) | | | 0.06 (-0.10, 0.21) | 0.43 | -0.02 (-0.09, 0.06) | 0.10 (-0.08, 0.28) | 0.27 |
|  | Overall mean | | -0.02 (-0.08, 0.05) | | | -0.02 (-0.16, 0.12) | 0.94 | -0.05 (-0.12, 0.02) | 0.07 (-0.14, 0.27) | 0.25 |
|  | DNOP metabolites | |  | | |  |  |  |  |  |
|  | First trimester | | -0.06 (-0.12, 0.00) | | | -0.06 (-0.20, 0.09) | 0.84 | -0.02 (-0.09, 0.06) | 0.05 (-0.14, 0.23) | 0.42 |
|  | Second trimester | | 0.02 (-0.05, 0.09) | | | 0.12 (-0.05, 0.28) | 0.26 | -0.01 (-0.09, 0.06) | 0.10 (-0.11, 0.31) | 0.26 |
|  | Third trimester | | -0.06 (-0.13, 0.00) | | | 0.07 (-0.09, 0.23) | 0.15 | -0.07 (-0.15, 0.00) | -0.07 (-0.27, 0.14) | 0.96 |
|  | Overall mean | | -0.07 (-0.13, 0.00)* | | | 0.04 (-0.11, 0.18) | 0.18 | -0.07 (-0.14, 0.01) | 0.02 (-0.18, 0.21) | 0.36 |
| Bone area (cm^2^) | | |  | | |  |  |  |  |  |
|  | Phthalic acid | |  | | |  |  |  |  |  |
|  | First trimester | | 0.47 (-2.56, 3.50) | | | 3.75 (-2.93, 10.44) | 0.47 | -2.31 (-6.39, 1.77) | 4.32 (-7.01, 15.65) | 0.54 |
|  | Second trimester | | -0.94 (-4.71, 2.83) | | | 2.88 (-4.47, 10.22) | 0.40 | -0.24 (-5.08, 4.60) | 1.64 (-10.09, 13.36) | 0.80 |
|  | Third trimester | | -2.00 (-5.27, 1.27) | | | -3.06 (-10.48, 4.36) | 0.85 | -3.72 (-8.05, 0.61) | 2.98 (-8.62, 14.57) | 0.66 |
|  | Overall mean | | -0.35 (-3.73, 3.03) | | | 1.38 (-5.95, 8.71) | 0.70 | -1.89 (-6.26, 2.50) | 0.96 (-12.54, 14.46) | 0.93 |
|  | LMW phthalate metabolites | |  | | |  |  |  |  |  |
|  | First trimester | | -0.75 (-4.35, 2.85) | | | 3.35 (-4.34, 11.05) | 0.66 | -2.54 (-7.40, 2.31) | 0.29 (-12.30, 12.88) | 0.86 |
|  | Second trimester | | -2.14 (-5.66, 1.37) | | | 2.73 (-4.45, 9.92) | 0.30 | -3.01 (-7.56, 1.54) | 0.94 (-10.51, 12.39) | 0.99 |
|  | Third trimester | | -2.29 (-5.98, 1.39) | | | -5.19 (-13.19, 2.80) | 0.33 | -3.71 (-8.54, 1.11) | 6.82 (-5.94, 19.58) | 0.44 |
|  | Overall mean | | -2.66 (-6.41, 1.09) | | | -0.39 (-8.73, 7.95) | 0.93 | -3.70 (-8.74, 1.34) | 1.04 (-13.58, 15.66) | 0.86 |
|  | HMW phthalate metabolites | |  | | |  |  |  |  |  |
|  | First trimester | | 1.46 (-1.67, 4.59) | | | 2.58 (-3.36, 8.51) | 0.99 | -1.83 (-6.12, 2.46) | -2.31 (-13.09, 8.47) | 0.52 |
|  | Second trimester | | -1.13 (-4.19, 1.94) | | | 1.01 (-5.19, 7.21) | 0.79 | 1.25 (-2.76, 5.26) | 3.20 (-6.80, 13.21) | 0.61 |
|  | Third trimester | | -1.40 (-4.66, 1.87) | | | -0.51 (-7.44, 6.43) | 0.95 | 0.50 (-3.69, 4.68) | 4.09 (-6.07, 14.25) | 0.41 |
|  | Overall mean | | 0.68 (-2.33, 3.68) | | | 2.15 (-3.56, 7.87) | 0.90 | 0.74 (-3.37, 4.85) | 0.58 (-10.00, 11.15) | 0.63 |
|  | DEHP metabolites | |  | | |  |  |  |  |  |
|  | First trimester | | 1.65 (-1.45, 4.74) | | | 3.21 (-2.60, 9.01) | 0.86 | -1.90 (-6.11, 2.30) | -0.60 (-11.11, 9.90) | 0.82 |
|  | Second trimester | | -1.49 (-4.62, 1.65) | | | 1.24 (-5.02, 7.49) | 0.66 | 1.12 (-2.91, 5.16) | 1.52 (-8.34, 11.39) | 0.55 |
|  | Third trimester | | -1.01 (-4.25, 2.24) | | | 1.02 (-5.73, 7.78) | 0.69 | 0.89 (-3.27, 5.04) | 4.81 (-5.22, 14.83) | 0.31 |
|  | Overall mean | | 0.79 (-2.19, 3.78) | | | 2.97 (-2.57, 8.52) | 0.69 | 0.80 (-3.21, 4.82) | 1.64 (-8.56, 11.83) | 0.88 |
|  | DNOP metabolites | |  | | |  |  |  |  |  |
|  | First trimester | | 2.33 (-0.60, 5.27) | | | 2.12 (-3.82, 8.06) | 0.75 | -1.31 (-5.29, 2.67) | -1.48 (-11.05, 8.10) | 0.73 |
|  | Second trimester | | -1.29 (-4.53, 1.96) | | | 1.27 (-5.49, 8.03) | 0.44 | 0.40 (-3.72, 4.53) | 1.61 (-9.33, 12.55) | 0.97 |
|  | Third trimester | | -0.57 (-3.70, 2.56) | | | -3.46 (-10.12, 3.20) | 0.63 | -0.21 (-4.26, 3.83) | 6.60 (-3.76, 16.94) | 0.25 |
|  | Overall mean | | 0.93 (-2.19, 4.05) | | | -0.57 (-6.64, 5.51) | 0.77 | 0.11 (-3.99, 4.21) | 1.32 (-8.61, 11.25) | 0.93 |
| Values are linear regression coefficients (β, 95% Confidence Interval) that reflect the differences in bone health for an interquartile range increase in each natural log-transformed phthalate urinary concentrations in µmol/g. Models include child’s age, sex, height and bonefree mass, maternal age, pre-pregnancy BMI, ethnicity and education level, parity, alcohol and smoking habits during each trimester and vitamin D blood concentrations.  *p-value <0.05 †significant after correction for multiple testing (p- value threshold of 0.0098)  mg/cm^2^, milligram per square centimeter; LMW phthalate, low molecular weight phthalate; HMW phthalate, high molecular weight phthalate; DEHP, di-2-ethylhexylphthalate; DNOP, di-n-octylphthalate; g, grams; cm^2^, square centimeter. | | | | | | | | | | |

**Table S16**. Associations of maternal bisphenol concentrations during first, second and third trimester with childhood bone mass at age 6 (sensitivity analysis, mutually adjusted model)

| Bisphenols | BMD (mg/cm^2^) | | BMC (g) | | aBMC (g) | | BA (cm^2^) | |
| --- | --- | --- | --- | --- | --- | --- | --- | --- |
|  | β (95% CI) | p-value | β (95% CI) | p-value | β (95% CI) | p-value | β (95% CI) | p-value |
| Total bisphenol |  |  |  |  |  |  |  |  |
| First trimester | -1.03 (-3.30, 1.23) | 0.37 | -1.03 (-3.62, 1.57) | 0.44 | -0.02 (-0.09, 0.04) | 0.52 | -0.35 (-3.32, 2.63) | 0.82 |
| Second trimester | 0.26 (-1.87, 2.38) | 0.81 | 0.66 (-1.77, 3.10) | 0.60 | 0.01 (-0.05, 0.07) | 0.78 | 0.45 (-2.34, 3.23) | 0.75 |
| Third trimester | 0.80 (-1.35, 2.95) | 0.47 | 0.49 (-1.97, 2.95) | 0.70 | 0.03 (-0.03, 0.09) | 0.38 | -0.63 (-3.46, 2.19) | 0.66 |
| BPA |  |  |  |  |  |  |  |  |
| First trimester | -0.51 (-2.81, 1.78) | 0.66 | -0.62 (-3.26, 2.01) | 0.64 | -0.01 (-0.07, 0.06) | 0.87 | -0.54 (-3.56, 2.47) | 0.72 |
| Second trimester | 0.27 (-1.83, 2.38) | 0.80 | 0.78 (-1.63, 3.19) | 0.53 | 0.01 (-0.05, 0.07) | 0.79 | 0.63 (-2.13, 3.39) | 0.66 |
| Third trimester | 1.22 (-0.87, 3.31) | 0.25 | 0.35 (-2.05, 2.74) | 0.78 | 0.05 (-0.01, 0.11) | 0.10 | -1.86 (-4.61, 0.88) | 0.18 |
| BPS |  |  |  |  |  |  |  |  |
| First trimester | -1.94 (-4.44, 0.57) | 0.13 | -0.58 (-3.45, 2.29) | 0.69 | -0.07 (-0.14, 0.01) | 0.07 | 2.21 (-1.08, 5.50) | 0.19 |
| Second trimester | -1.01 (-3.12, 1.10) | 0.35 | -2.00 (-4.42, 0.42) | 0.11 | -0.01 (-0.07, 0.06) | 0.83 | -2.27 (-5.04, 0.50) | 0.11 |
| Third trimester | 1.19 (-0.73, 3.10) | 0.23 | 0.96 (-1.24, 3.16) | 0.39 | 0.03 (-0.02, 0.09) | 0.24 | -0.28 (-2.80, 2.24) | 0.83 |
| BPF |  |  |  |  |  |  |  |  |
| First trimester | -0.47 (-2.86, 1.91) | 0.70 | 0.19 (-2.54, 2.93) | 0.89 | -0.01 (-0.08, 0.06) | 0.72 | 0.81 (-2.33, 3.95) | 0.61 |
| Second trimester | NA |  | NA |  | NA |  | NA |  |
| Third trimester | -0.06 (-2.44, 2.33) | 0.96 | 0.75 (-1.98, 3.48) | 0.59 | -0.01 (-0.08, 0.06) | 0.79 | 1.39 (-1.74, 4.52) | 0.38 |

Values are linear regression coefficients (β, 95% Confidence Interval) that reflect the differences in bone health for an interquartile range increase in each natural log-transformed bisphenol urinary concentrations in µmol/g. Mutually adjusted model includes bisphenol concentrations in first, second and third trimester and child’s age, height and bonefree mass, maternal age, pre-pregnancy BMI, ethnicity and education level, parity, folic acid supplement use during pregnancy, alcohol and smoking habits during each trimester and vitamin D blood concentrations.

BMD (mg/cm^2^), bone mineral density (milligram per square centimeter); BMC (g), bone mineral content (grams); aBMC (g), area adjusted bone mineral content (grams); BA (cm^2^), bone area (square centimeter); CI, Confidence Interval; BPA, bisphenol A; BPS, bisphenol S, BPF, bisphenol F; NA, not applicable due to low detection rates.

**Table S17.** Associations of maternal bisphenol concentrations during first, second and third trimester with childhood bone mass at age 10 (sensitivity analysis, mutually adjusted model)

| Bisphenols | BMD (mg/cm^2^) | | BMC (g) | | aBMC (g) | | BA (cm^2^) | |
| --- | --- | --- | --- | --- | --- | --- | --- | --- |
|  | β (95% CI) | p-value | β (95% CI) | p-value | β (95% CI) | p-value | β (95% CI) | p-value |
| Total bisphenol |  |  |  |  |  |  |  |  |
| First trimester | -2.80 (-6.30, 0.71) | 0.12 | -1.35 (-7.08, 4.38) | 0.64 | -0.07 (-0.14, 0.01) | 0.08 | 2.73 (-1.31, 6.77) | 0.19 |
| Second trimester | 0.91 (-2.46, 4.28) | 0.60 | 0.35 (-5.16, 5.87) | 0.90 | 0.03 (-0.05, 0.10) | 0.49 | -1.19 (-5.07, 2.68) | 0.55 |
| Third trimester | -0.77 (-4.11, 2.58) | 0.65 | -0.41 (-5.89, 5.07) | 0.88 | -0.02 (-0.09, 0.05) | 0.60 | 0.78 (-3.07, 4.63) | 0.69 |
| BPA |  |  |  |  |  |  |  |  |
| First trimester | -1.31 (-4.87, 2.25) | 0.47 | 0.39 (-5.43, 6.21) | 0.90 | -0.03 (-0.10, 0.05) | 0.45 | 2.09 (-2.01, 6.19) | 0.32 |
| Second trimester | 0.85 (-2.50, 4.20) | 0.62 | 0.48 (-5.00, 5.96) | 0.86 | 0.02 (-0.05, 0.09) | 0.53 | -0.91 (-4.76, 2.94) | 0.64 |
| Third trimester | -1.11 (-4.37, 2.15) | 0.51 | -0.90 (-6.23, 4.42) | 0.74 | -0.03 (-0.09, 0.04) | 0.44 | 0.81 (-2.94, 4.55) | 0.67 |
| BPS |  |  |  |  |  |  |  |  |
| First trimester | -5.88 (-9.78, -1.98)† | <0.01 | -7.11 (-13.49, -0.73)* | 0.03 | -0.12 (-0.20, -0.04)† | <0.01 | 0.96 (-3.52, 5.45) | 0.67 |
| Second trimester | -0.12 (-3.37, 3.13) | 0.94 | -3.18 (-8.51, 2.15) | 0.24 | 0.02 (-0.05, 0.09) | 0.55 | -4.07 (-7.82, -0.32)* | 0.03 |
| Third trimester | 1.14 (-1.96, 4.25) | 0.47 | 0.98 (-4.11, 6.05) | 0.71 | 0.04 (-0.03, 0.10) | 0.27 | -1.38 (-4.91, 2.24) | 0.46 |
| BPF |  |  |  |  |  |  |  |  |
| First trimester | -1.45 (-5.24, 2.35) | 0.46 | 1.62 (-4.60, 7.83) | 0.61 | -0.06 (-0.14, 0.02) | 0.14 | 5.01 (0.65, 9.37)* | 0.02 |
| Second trimester | NA |  | NA |  | NA |  | NA |  |
| Third trimester | 1.13 (-2.64, 4.90) | 0.56 | 0.81 (-5.35, 6.96) | 0.80 | 0.05 (-0.03, 0.12) | 0.26 | -2.03 (-6.35, 2.29) | 0.36 |

Values are linear regression coefficients (β, 95% Confidence Interval) that reflect the differences in bone health for an interquartile range increase in each natural log-transformed bisphenol urinary concentrations in µmol/g. Mutually adjusted model includes bisphenol concentrations in first, second and third trimester and child’s age, height and bonefree mass, maternal age, pre-pregnancy BMI, ethnicity and education level, parity, folic acid supplement use during pregnancy, alcohol and smoking habits during each trimester and vitamin D blood concentrations.

*p-value <0.05 †significant after correction for multiple testing (p- value threshold of 0.0098)

BMD (mg/cm^2^), bone mineral density (milligram per square centimeter); BMC (g), bone mineral content (grams); aBMC (g), area adjusted bone mineral content (grams); BA (cm^2^), bone area (square centimeter); CI, Confidence Interval; BPA, bisphenol A; BPS, bisphenol S, BPF, bisphenol F; NA, not applicable due to low detection rates.

**Table S18**. Associations of maternal phthalate concentrations during first, second and third trimester with childhood bone mass at age 6 (sensitivity analysis, mutually adjusted model)

| Phthalates | BMD (mg/cm^2^) | | BMC (g) | | aBMC (g) | | BA (cm^2^) | |
| --- | --- | --- | --- | --- | --- | --- | --- | --- |
|  | β (95% CI) | p-value | β (95% CI) | p-value | β (95% CI) | p-value | β (95% CI) | p-value |
| Phthalic acid |  |  |  |  |  |  |  |  |
| First trimester | -0.07 (-2.12, 1.99) | 0.95 | 0.61 (-1.75, 2.96) | 0.61 | -0.01 (-0.07, 0.05) | 0.75 | 1.20 (-1.50, 3.90) | 0.38 |
| Second trimester | 0.92 (-1.60, 3.43) | 0.48 | 0.91 (-1.98, 3.79) | 0.54 | 0.02 (-0.05, 0.10) | 0.54 | 0.13 (-3.17, 3.44) | 0.94 |
| Third trimester | -0.95 (-3.20, 1.30) | 0.41 | -2.02 (-4.61, 0.56) | 0.13 | -0.01 (-0.07, 0.06) | 0.87 | -2.35 (-5.31, 0.61) | 0.12 |
| LMW phthalate metabolites |  |  |  |  |  |  |  |  |
| First trimester | -1.25 (-3.68, 1.20) | 0.32 | -0.66 (-3.47, 2.15) | 0.65 | -0.04 (-0.11, 0.04) | 0.33 | 0.72 (-2.49, 3.94) | 0.66 |
| Second trimester | 0.72 (-1.82, 3.26) | 0.58 | 0.61 (-2.30, 3.53) | 0.68 | 0.03 (-0.05, 0.10) | 0.51 | -0.31 (-3.64, 3.02) | 0.86 |
| Third trimester | 1.98 (-0.69, 4.65) | 0.15 | 0.17 (-2.89, 3.24) | 0.91 | 0.08 (0.00, 0.16)* | 0.04 | -3.36 (-6.87, 0.14) | 0.06 |
| HMW phthalate metabolites |  |  |  |  |  |  |  |  |
| First trimester | -0.66 (-2.76, 1.45) | 0.54 | 0.13 (-2.28, 2.53) | 0.92 | -0.04 (-0.10, 0.03) | 0.27 | 1.70 (-1.05, 4.46) | 0.23 |
| Second trimester | 0.70 (-1.35, 2.74) | 0.51 | 0.15 (-2.19, 2.50) | 0.90 | 0.03 (-0.03, 0.09) | 0.40 | -0.95 (-3.64, 1.74) | 0.49 |
| Third trimester | 0.09 (-2.05, 2.24) | 0.93 | -0.66 (-3.12, 1.80) | 0.60 | 0.01 (-0.05, 0.07) | 0.77 | -1.27 (-4.08, 1.55) | 0.38 |
| DEHP metabolites |  |  |  |  |  |  |  |  |
| First trimester | -0.35 (-2.42, 1.71) | 0.74 | 0.57 (-1.80, 2.93) | 0.64 | -0.03 (-0.09, 0.03) | 0.35 | 2.01 (-0.70, 4.72) | 0.15 |
| Second trimester | 0.21 (-1.86, 2.27) | 0.85 | -0.49 (-2.85, 1.88) | 0.69 | 0.02 (-0.05, 0.08) | 0.62 | -1.30 (-4.01, 1.40) | 0.35 |
| Third trimester | 0.27 (-1.87, 2.40) | 0.81 | -0.07 (-2.52, 2.37) | 0.95 | 0.01 (-0.05, 0.07) | 0.74 | -0.57 (-3.37, 2.23) | 0.69 |
| DNOP metabolites |  |  |  |  |  |  |  |  |
| First trimester | -1.46 (-3.46, 0.55) | 0.15 | 0.02 (-2.28, 2.32) | 0.99 | -0.06 (-0.12, 0.00) | 0.06 | 2.54 (-0.09, 5.18) | 0.06 |
| Second trimester | 1.68 (-0.51, 3.87) | 0.13 | 0.91 (-1.60, 3.42) | 0.48 | 0.05 (-0.01, 0.12) | 0.11 | -1.17 (-4.05, 1.71) | 0.43 |
| Third trimester | -1.31 (-3.40, 0.79) | 0.22 | -2.21 (-4.62, 0.19) | 0.07 | -0.03 (-0.09, 0.03) | 0.33 | -1.48 (-4.23, 1.28) | 0.29 |

Values are linear regression coefficients (β, 95% Confidence Interval) that reflect the differences in bone health for an interquartile range increase in each natural log-transformed phthalate urinary concentrations in µmol/g. Mutually adjusted model includes phthalate concentrations in first, second and third trimester and child’s age, height and bonefree mass, maternal age, pre-pregnancy BMI, ethnicity and education level, parity, folic acid supplement use during pregnancy, alcohol and smoking habits during each trimester and vitamin D blood concentrations.

*p-value <0.05

BMD (mg/cm^2^), bone mineral density (milligram per square centimeter); BMC (g), bone mineral content (grams); aBMC (g), area adjusted bone mineral content (grams); BA (cm^2^), bone area (square centimeter); CI, Confidence Interval; LMW phthalate, low molecular weight phthalate; HMW phthalate, high molecular weight phthalate; DEHP, di-2-ethylhexylphthalate; DNOP, di-n-octylphthalate.

**Table S19.** Associations of maternal phthalate concentrations during first, second and third trimester with childhood bone mass at age 10 (sensitivity analysis, mutually adjusted model)

| Phthalates | BMD (mg/cm^2^) | | BMC (g) | | aBMC (g) | | BA (cm^2^) | |
| --- | --- | --- | --- | --- | --- | --- | --- | --- |
|  | β (95% CI) | p-value | β (95% CI) | p-value | β (95% CI) | p-value | β (95% CI) | p-value |
| Phthalic acid |  |  |  |  |  |  |  |  |
| First trimester | 0.32 (-2.89, 3.53) | 0.85 | -0.12 (-5.36, 5.13) | 0.97 | 0.02 (-0.05, 0.08) | 0.63 | -1.08 (-4.78, 2.61) | 0.57 |
| Second trimester | -0.50 (-4.41, 3.42) | 0.81 | -0.79 (-7.19, 5.61) | 0.81 | -0.02 (-0.10, 0.06) | 0.68 | 0.34 (-4.16, 4.84) | 0.88 |
| Third trimester | -2.66 (-6.13, 0.82) | 0.13 | -5.07 (-10.74, 0.61) | 0.08 | -0.03 (-0.10, 0.04) | 0.38 | -2.52 (-6.52, 1.48) | 0.22 |
| LMW phthalate metabolites |  |  |  |  |  |  |  |  |
| First trimester | -0.52 (-4.40, 3.36) | 0.79 | -0.69 (-7.03, 5.64) | 0.83 | 0.01 (-0.07, 0.09) | 0.77 | -1.33 (-5.79, 3.13) | 0.56 |
| Second trimester | -0.69 (-4.62, 3.24) | 0.73 | -2.65 (-9.06, 3.77) | 0.42 | -0.01 (-0.10, 0.07) | 0.75 | -1.54 (-6.05, 2.96) | 0.50 |
| Third trimester | 0.00 (-4.10, 4.10) | 1.00 | -0.53 (-7.22, 6.17) | 0.88 | 0.02 (-0.07, 0.10) | 0.66 | -1.61 (-6.32, 3.10) | 0.50 |
| HMW phthalate metabolites |  |  |  |  |  |  |  |  |
| First trimester | -0.49 (-3.87, 2.90) | 0.78 | -2.03 (-7.56, 3.50) | 0.47 | 0.01 (-0.06, 0.08) | 0.84 | -2.24 (-6.12, 1.65) | 0.26 |
| Second trimester | -0.06 (-3.30, 3.17) | 0.97 | 0.20 (-5.09, 5.48) | 0.94 | -0.02 (-0.08, 0.05) | 0.64 | 1.13 (-2.58, 4.85) | 0.55 |
| Third trimester | -0.18 (-3.53, 3.16) | 0.91 | 0.61 (-4.86, 6.07) | 0.83 | -0.01 (-0.08, 0.06) | 0.78 | 1.14 (-2.71, 4.98) | 0.56 |
| DEHP metabolites |  |  |  |  |  |  |  |  |
| First trimester | -0.13 (-3.45, 3.19) | 0.94 | -1.51 (-6.94, 3.92) | 0.59 | 0.00 (-0.06, 0.08) | 0.75 | -2.00 (-5.82, 1.81) | 0.30 |
| Second trimester | -0.59 (-3.85, 2.66) | 0.72 | -0.80 (-6.11, 4.52) | 0.77 | -0.02 (-0.09, 0.05) | 0.51 | 0.67 (-3.07, 4.41) | 0.73 |
| Third trimester | 0.54 (-2.79, 3.87) | 0.75 | 2.09 (-3.35, 7.53) | 0.45 | 0.01 (-0.07, 0.07) | 0.94 | 1.68 (-2.15, 5.51) | 0.39 |
| DNOP metabolites |  |  |  |  |  |  |  |  |
| First trimester | -0.48 (-3.66, 2.69) | 0.77 | -1.03 (-6.22, 4.15) | 0.70 | 0.01 (-0.05, 0.08) | 0.73 | -1.62 (-5.28, 2.04) | 0.39 |
| Second trimester | 0.60 (-2.80, 3.99) | 0.73 | 1.18 (-4.38, 6.73) | 0.68 | 0.00 (-0.07, 0.07) | 0.92 | 0.81 (-3.10, 4.72) | 0.69 |
| Third trimester | -2.99 (-6.26, 0.28) | 0.07 | -4.30 (-9.64, 1.05) | 0.12 | -0.07 (-0.14, 0.00)* | 0.04 | 0.56 (-3.21, 4.33) | 0.77 |

Values are linear regression coefficients (β, 95% Confidence Interval) that reflect the differences in bone health for an interquartile range increase in each natural log-transformed phthalate urinary concentrations in µmol/g. Mutually adjusted model includes phthalate concentrations in first, second and third trimester and child’s age, height and bonefree mass, maternal age, pre-pregnancy BMI, ethnicity and education level, parity, folic acid supplement use during pregnancy, alcohol and smoking habits during each trimester and vitamin D blood concentrations.

*p-value <0.05

BMD (mg/cm^2^), bone mineral density (milligram per square centimeter); BMC (g), bone mineral content (grams); aBMC (g), area adjusted bone mineral content (grams); BA (cm^2^), bone area (square centimeter); CI, Confidence Interval; LMW phthalate, low molecular weight phthalate; HMW phthalate, high molecular weight phthalate; DEHP, di-2-ethylhexylphthalate; DNOP, di-n-octylphthalate.

**Table S20**. Associations of maternal bisphenol concentrations with childhood bone mass (sensitivity analysis by adding creatinine as a covariate in the model)

|  | Age 6 | | | | Age 10 | | | |  |  |
| --- | --- | --- | --- | --- | --- | --- | --- | --- | --- | --- |
| Bisphenols | BMD (mg/cm^2^) | | aBMC (g) | | BMD (mg/cm^2^) | | aBMC (g) | |  |  |
|  | β (95% CI) | p-value | β (95% CI) | p-value | β (95% CI) | p-value | β (95% CI) | p-value |  |  |
| Total bisphenol |  |  |  |  |  |  |  |  |  |  |
| First trimester | -0.31 (-2.85, 2.23) | 0.81 | 0.01 (-0.07, 0.08) | 0.84 | -1.68 (-5.63, 2.26) | 0.40 | -0.05 (-0.13, 0.03) | 0.26 |  |  |
| Second trimester | 0.65 (-1.66, 2.96) | 0.58 | 0.01 (-0.05, 0.08) | 0.71 | 1.77 (-1.90, 5.44) | 0.34 | 0.04 (-0.03, 0.12) | 0.27 |  |  |
| Third trimester | 0.94 (-1.48, 3.36) | 0.45 | 0.03 (-0.04, 0.10) | 0.42 | -0.60 (-4.39, 3.20) | 0.76 | -0.03 (-0.11, 0.05) | 0.48 |  |  |
| BPA |  |  |  |  |  |  |  |  |  |  |
| First trimester | 0.07 (-2.74, 2.89) | 0.96 | 0.02 (-0.06, 0.10) | 0.64 | -0.33 (-4.68, 4.01) | 0.88 | -0.01 (-0.10, 0.08) | 0.80 |  |  |
| Second trimester | 0.68 (-1.57, 2.93) | 0.55 | 0.01 (-0.05, 0.08) | 0.67 | 1.64 (-1.95, 5.22) | 0.37 | 0.04 (-0.04, 0.11) | 0.32 |  |  |
| Third trimester | 1.20 (-1.10, 3.51) | 0.31 | 0.05 (-0.02, 0.12) | 0.14 | -0.85 (-4.50, 2.80) | 0.65 | -0.03 (-0.10, 0.05) | 0.46 |  |  |
| BPS |  |  |  |  |  |  |  |  |  |  |
| First trimester | -1.54 (-4.62, 1.54) | 0.33 | -0.05 (-0.14, 0.04) | 0.26 | -5.98 (-10.81, -1.15)* | 0.02 | -0.12 (-0.22, -0.02)* | 0.02 |  |  |
| Second trimester | -0.44 (-2.32, 1.43) | 0.65 | 0.00 (-0.05, 0.06) | 0.99 | 0.67 (-2.22, 3.56) | 0.65 | 0.03 (-0.03, 0.09) | 0.31 |  |  |
| Third trimester | NA |  | NA |  | NA |  | NA |  |  |  |
| BPF |  |  |  |  |  |  |  |  |  |  |
| First trimester | 0.68 (-1.29, 2.64) | 0.50 | 0.03 (-0.03, 0.08) | 0.38 | 0.75 (-2.42, 3.91) | 0.64 | -0.02 (-0.09, 0.04) | 0.52 |  |  |
| Second trimester | NA |  | NA |  | NA |  | NA |  |  |  |
| Third trimester | 0.37 (-1.79, 2.53) | 0.74 | 0.00 (-0.07, 0.06) | 0.90 | 1.28 (-2.12, 4.68) | 0.46 | 0.03 (-0.04, 0.10) | 0.37 |  |  |

Values are linear regression coefficients (β, 95% Confidence Interval) that reflect the differences in bone health for an interquartile range increase in each natural log-transformed bisphenol urinary concentrations in nmol/L urine. Models include maternal urinary creatinine concentration, child’s age, sex, height and bonefree mass, maternal age, pre-pregnancy BMI, ethnicity and education level, parity, folic acid supplement use during pregnancy, alcohol and smoking habits during each trimester and vitamin D blood concentrations.

*p-value <0.05

BMD (mg/cm^2^), bone mineral density (milligram per square centimeter); BMC (g), bone mineral content (grams); aBMC (g), area adjusted bone mineral content (grams); BA (cm^2^), bone area (square centimeter); CI, Confidence Interval; BPA, bisphenol A; BPS, bisphenol S, BPF, bisphenol F; NA, not applicable due to low detection rates.

**Table S21**. Associations of maternal phthalate concentrations with childhood bone mass (sensitivity analysis by adding creatinine as a covariate in the model)

|  |  | Age 6 | |  |  | Age 10 | |  |
| --- | --- | --- | --- | --- | --- | --- | --- | --- |
| Phthalates | BMD (mg/cm^2^) | | aBMC (g) | | BMD (mg/cm^2^) | | aBMC (g) | |
|  | β (95% CI) | p-value | β (95% CI) | p-value | β (95% CI) | p-value | β (95% CI) | p-value |
| Phthalic acid |  |  |  |  |  |  |  |  |
| First trimester | -0.66 (-1.58, 2.91) | 0.56 | 0.02 (-0.04, 0.09) | 0.53 | 1.39 (-2.14, 4.91) | 0.44 | 0.04 (-0.03, 0.11) | 0.30 |
| Second trimester | 1.88 (-0.86, 4.63) | 0.18 | 0.04 (-0.04, 0.12) | 0.34 | 0.86 (-3.42, 5.13 ) | 0.69 | 0.01 (-0.08, 0.10) | 0.87 |
| Third trimester | -1.16 (-3.86, 1.55) | 0.40 | -0.01 (-0.09, 0.07) | 0.74 | -2.47 (-6.69, 1.75) | 0.25 | -0.03 (-0.12, 0.05) | 0.45 |
| LMW phthalate metabolites |  |  |  |  |  |  |  |  |
| First trimester | 0.09 (-2.44, 2.61) | 0.95 | 0.02 (-0.05, 0.09) | 0.61 | 0.38 (-3.60, 4.35) | 0.85 | 0.04 (-0.05, 0.12) | 0.40 |
| Second trimester | 1.44 (-1.21, 4.09) | 0.29 | 0.05 (-0.03, 0.12) | 0.24 | -0.02 (-4.13, 4.09) | 0.99 | 0.02 (-0.07, 0.10) | 0.74 |
| Third trimester | 1.89 (-0.83, 4.61) | 0.17 | 0.08 (0.00, 0.16) | 0.06 | -0.03 (-4.26, 4.21) | 0.99 | 0.02 (-0.07, 0.11) | 0.67 |
| HMW phthalate metabolites |  |  |  |  |  |  |  |  |
| First trimester | 0.39 (-1.99, 2.77) | 0.75 | 0.00 (-0.07, 0.07) | 0.95 | 1.48 (-2.34, 5.31) | 0.45 | 0.04 (-0.04, 0.12) | 0.28 |
| Second trimester | 1.32 (-0.92, 3.56) | 0.25 | 0.03 (-0.03, 0.10) | 0.32 | 1.16 (-2.38, 4.70) | 0.52 | 0.01 (-0.07, 0.08) | 0.89 |
| Third trimester | 0.29 (-2.48, 3.06) | 0.84 | 0.01 (-0.07, 0.09) | 0.83 | 0.67 (-3.68, 5.02) | 0.76 | -0.01 (-0.10, 0.08) | 0.76 |
| DEHP metabolites |  |  |  |  |  |  |  |  |
| First trimester | 0.63 (-1.68, 2.94) | 0.59 | 0.01 (-0.06, 0.07) | 0.87 | 1.76 (-1.92, 5.44) | 0.35 | 0.05 (-0.03, 0.12) | 0.24 |
| Second trimester | 0.81 (-1.48, 3.10) | 0.49 | 0.02 (-0.05, 0.09) | 0.53 | 0.53 (-3.07, 4.13) | 0.77 | 0.00 (-0.08, 0.07) | 0.93 |
| Third trimester | 0.46 (-2.27, 3.19) | 0.74 | 0.01 (-0.07, 0.09) | 0.81 | 1.48 (-2.82, 5.78) | 0.50 | 0.00 (-0.09, 0.09) | 0.98 |
| DNOP metabolites |  |  |  |  |  |  |  |  |
| First trimester | -0.73 (-2.96, 1.50) | 0.52 | -0.03 (-0.09, 0.04) | 0.39 | 0.82 (-2.75, 4.38) | 0.65 | 0.03 (-0.04, 0.11) | 0.37 |
| Second trimester | 1.99 (-0.32, 4.30) | 0.09 | 0.05 (-0.02, 0.12) | 0.16 | 1.52 (-2.09, 5.13) | 0.41 | 0.02 (-0.05, 0.10) | 0.57 |
| Third trimester | -1.69 (-4.31, 0.93) | 0.21 | -0.05 (-0.12, 0.03) | 0.22 | -2.93 (-6.99, 1.13) | 0.16 | -0.08 (-0.17, 0.00) | 0.05 |

Values are linear regression coefficients (β, 95% Confidence Interval) that reflect the differences in bone health for an interquartile range increase in each natural log-transformed phthalate urinary concentrations in nmol/L urine. Models include maternal urinary creatinine concentration, child’s age, sex, height and bonefree mass, maternal age, pre-pregnancy BMI, ethnicity and education level, parity, folic acid supplement use during pregnancy, alcohol and smoking habits during each trimester and vitamin D blood concentrations.

BMD (mg/cm^2^), bone mineral density (milligram per square centimeter); BMC (g), bone mineral content (grams); aBMC (g), area adjusted bone mineral content (grams); BA (cm^2^), bone area (square centimeter); CI, Confidence Interval; LMW phthalate, low molecular weight phthalate; HMW phthalate, high molecular weight phthalate; DEHP, di-2-ethylhexylphthalate; DNOP, di-n-octylphthalate.

**Table S22**. Associations of maternal bisphenol concentrations with childhood bone mass (sensitivity analysis by using bisphenol concentrations categorized as detected and undetected)

|  | Age 6 | | | | Age 10 | | | |
| --- | --- | --- | --- | --- | --- | --- | --- | --- |
| Bisphenols | BMD (mg/cm^2^) | | aBMC (g) | | BMD (mg/cm^2^) | | aBMC (g) | |
|  | β (95% CI) | p-value | β (95% CI) | p-value | β (95% CI) | p-value | β (95% CI) | p-value |
| BPA |  |  |  |  |  |  |  |  |
| First trimester | 1.76 (-2.51, 6.04) | 0.42 | 0.09 (-0.04, 0.21) | 0.16 | 2.29 (-4.26, 8.83) | 0.49 | 0.08 (-0.06, 0.21) | 0.26 |
| Second trimester | -1.13 (-7.93, 5.68) | 0.75 | -0.04 (-0.24, 0.16) | 0.71 | 1.59 (-9.32, 12.50) | 0.78 | 0.03 (-0.20, 0.26) | 0.80 |
| Third trimester | -0.78 (-6.64, 5.07) | 0.79 | 0.02 (-0.15, 0.19) | 0.84 | -5.30 (-14.69, 4.09) | 0.27 | -0.12 (-0.31, 0.08) | 0.24 |
| BPS |  |  |  |  |  |  |  |  |
| First trimester | -0.78 (-4.50, 2.94) | 0.68 | -0.07 (-0.17, 0.04) | 0.23 | -4.67 (-10.50, 1.16) | 0.12 | -0.13 (-0.25, -0.01)* | 0.03 |
| Second trimester | -0.60 (-4.39, 3.20) | 0.76 | 0.00 (-0.11, 0.11) | 0.99 | 1.19 (-4.75, 7.13) | 0.70 | 0.04 (-0.08, 0.16) | 0.52 |
| Third trimester | 3.87 (-0.49, 8.24) | 0.08 | 0.09 (-0.04, 0.22) | 0.17 | 2.29 (-4.66, 9.24) | 0.52 | 0.02 (-0.12, 0.17) | 0.76 |
| BPF |  |  |  |  |  |  |  |  |
| First trimester | 1.08 (-2.46, 4.62) | 0.55 | 0.02 (-0.08, 0.13) | 0.65 | -0.72 (-6.26, 4.82) | 0.80 | -0.08 (-0.19, 0.04) | 0.19 |
| Second trimester | NA |  | NA |  | NA |  | NA |  |
| Third trimester | 1.17 (-2.63, 4.98) | 0.55 | 0.02 (-0.09, 0.13) | 0.72 | 2.50 (-3.55, 8.56) | 0.42 | 0.07 (-0.06, 0.19) | 0.29 |

Values are linear regression coefficients (β, 95% Confidence Interval) that reflect the differences in bone health for detected bisphenol concentrations compared with the reference group (undetected concentrations). Models include child’s age, sex, height and bonefree mass, maternal age, pre-pregnancy BMI, ethnicity and education level, parity, folic acid supplement use during pregnancy, alcohol and smoking habits during each trimester and vitamin D blood concentrations.

*p-value <0.05

BMD (mg/cm^2^), bone mineral density (milligram per square centimeter); BMC (g), bone mineral content (grams); aBMC (g), area adjusted bone mineral content (grams); BA (cm^2^), bone area (square centimeter); CI, Confidence Interval; BPA, bisphenol A; BPS, bisphenol S, BPF, bisphenol F; NA, not applicable due to low detection rates.
